# c-di-GMP phosphodiesterase ProE interacts with quorum sensing protein PqsE to promote pyocyanin production in *Pseudomonas aeruginosa*

**DOI:** 10.1128/msphere.01026-24

**Published:** 2025-01-28

**Authors:** Qishun Feng, Xin Dai, Qiulan Wu, Lianhui Zhang, Liang Yang, Yang Fu

**Affiliations:** 1 National Clinical Research Center for Infectious Disease, Shenzhen Third People's Hospital, The Second Affiliated Hospital of Southern University of Science and Technology 255310 , Shenzhen, China; 2 School of Medicine, Southern University of Science and Technology, Shenzhen, China; 3 Guangdong Province Key Laboratory of Microbial Signals and Disease Control, Integrative Microbiology Research Centre, South China Agricultural University, Guangzhou, China; 4 Guangdong Laboratory for Lingnan Modern Agriculture, South China Agricultural University, Guangzhou, China; 5 Institute for Biological Electron Microscopy, Southern University of Science and Technology, Shenzhen, China; The University of Texas Medical Branch at Galveston, Galveston, Texas, USA

**Keywords:** *Pseudomonas aeruginosa*, c-di-GMP phosphodiesterase, quorum sensing, protein-protein interaction, pyocyanin

## Abstract

The universal bacterial second messenger bis-(3′-5′)-cyclic dimeric guanosine monophosphate (c-di-GMP) plays critical roles in regulating a variety of bacterial functions such as biofilm formation and virulence. The metabolism of c-di-GMP is inversely controlled by diguanylate cyclases (DGCs) and phosphodiesterases (PDEs). Recently, increasing studies suggested that the protein-protein interactions between DGCs/PDEs and their partners appear to be a common way to achieve specific regulation. In this work, we showed that the PDE ProE can interact with PQS quorum sensing protein PqsE to regulate pyocyanin production in *Pseudomonas aeruginosa*. Our bacterial two-hybrid assay demonstrated that ProE directly interacts with PqsE, and isothermal titration calorimetry and surface plasmon resonance assay further confirmed that the binding affinity of ProE with PqsE is at micromolar level. Both ProE and PqsE negatively regulate intracellular c-di-GMP levels. Furthermore, our transcriptomic study showed that co-expression of ProE and PqsE significantly changes the gene expression profiles in *P. aeruginosa*, especially with increased expression of pyocyanin genes, and the qPCR and phenotypic results confirmed the transcriptome data. Taken together, our study suggested that the interaction between ProE and PqsE plays a critical role in regulation of pyocyanin production and highlights the importance of protein-protein interaction mediated c-di-GMP signaling in *P. aeruginosa*.

**IMPORTANCE:**

c-di-GMP is pivotal in orchestrating various bacterial functions. In P*seudomonas aeruginosa*, the nuanced balance of intracellular c-di-GMP is maintained by approximately 41 diguanylate cyclases (DGCs) and phosphodiesterases (PDEs). Emerging studies indicate that the c-di-GMP metabolic DGCs and PDEs may be involved in the signal transduction process by directly binding to the target protein, thus influencing downstream function. Despite their known importance, the precise functions of these proteins, especially their interacting partners, remain unclear. In this study, we identified that PQS quorum sensing system protein PqsE is a binding partner of c-di-GMP phosphodiesterase ProE; further analysis suggested that the ProE specifically interacts with PqsE to promote pyocyanin production. Our study extended the regulatory mechanism of the c-di-GMP signal transduction and quorum sensing in governing bacterial physiology.

## INTRODUCTION

The opportunistic *Pseudomonas aeruginosa* is a notorious human pathogen, which can severely infect patients with burn wounds, ventilator-associated pneumonia, catheter-associated infections, or cystic fibrosis (1, 2). The ability of *P. aeruginosa* to cause acute or chronic infections in the host is attributed to its complicated virulence mechanisms, such as bis-(3′-5′)-cyclic dimeric guanosine monophosphate (c-di-GMP) signaling (3), quorum sensing (QS) (4, 5), and epigenetic control (6).

c-di-GMP is an intracellular second messenger, which regulates many important biological functions in bacteria, such as biofilm formation, secretion systems, antibiotic resistance, and virulence (7, 8). Intracellular c-di-GMP levels are inversely controlled by diguanylate cyclases (DGCs) and phosphodiesterases (PDEs), which can synthesize or degrade c-di-GMP, respectively (9). The c-di-GMP functions by binding to various effectors and controlling downstream phenotypes at transcriptional, translational, and posttranslational levels (8).

Quorum sensing is another regulation system that plays a key role in modulating bacterial group behaviors (10). It is an intercellular communication system that depends on cell density and is essential for controlling the pathogenicity and biofilms in bacteria including *P. aeruginosa* (4). The *P. aeruginosa* QS network consists of two LuxIR homologs of AHL-dependent signaling systems (*las* and *rhl*), as well as an additional *pqs* system based on the signal 2-heptyl-3-hydroxy-4-(1H)-quinolone (PQS) (10). The *las* and *rhl* transcriptionally controlled the production of extracellular virulence factors, such as elastase, alkaline protease, lasA protease, exotoxin A, rhamnolipids, pyocyanin, hydrogen cyanide, cytotoxic lectins PA-IL and PA-IIL, and bacterial motility (11). The PQS system regulates AHL-associated virulence factors (11). Among these virulence factors, pyocyanin is one of the most important, owing to its vital role in the pathogenesis of this bacteria (12). The above three QS systems constitute a hierarchical regulatory network, and the expression of the *pqs* and *rhl* systems is governed by *las* system (13, 14).

Previous studies showed that the c-di-GMP and QS are closely related in *P. aeruginosa*, and the QS receptor LasR can regulate the expression of tyrosine phosphatase encoding gene *tpbA*, thus regulating the activity of DGC TpbB (also called YfiN) (15). The null mutation of *lasI* or *lasR* can decrease the intracellular c-di-GMP levels, while mutation of *rhlI* increased c-di-GMP levels (16). However, it is not fully understood how QS and c-di-GMP are connected to regulate the biological functions of *P. aeruginosa*.

Emerging studies show that c-di-GMP metabolic DGCs and PDEs could participate in the signal transduction process by directly binding to the target protein, thus influencing downstream function (17–19). For example, a recent study reported that a PDE RpfR can interact with a transcription regulator GtrR to enhance its binding to target gene promoters (19). The wildly used laboratory *P. aeruginosa* PAO1 strain has a total of 41 putative DGCs and PDEs, and about one-fourth of them were reported to function by interacting with their partners in recent years, including ImcA, SiaD, RoeA, YfiN, HsbD, SadC, GcbA, NicD, RmcA, RocR, and MorA (17, 18, 20–30). Therefore, we hypothesized that the rest DGCs or PDEs could also function similarly. Elucidating the binding proteins of these DGCs and PDEs as well as the underlying mechanism will provide new insights into the c-di-GMP signaling.

In our previous work, we functionally characterized a PDE ProE, which specifically regulates exopolysaccharide (EPS) production and possesses high PDE activity (31). However, we still lack a comprehensive understanding of the role that ProE plays in signal transduction. We assumed that ProE may function by binding to its partner protein; hence, we aimed to identify the potential binding protein of ProE and study the underlying mechanism. In this study, we identified the PQS quorum sensing system protein PqsE as a binding partner of ProE, and the null mutations in either *proE* or *pqsE* resulted in increased intracellular c-di-GMP levels. Our findings indicate that the interaction between ProE and PqsE is unique among the PDEs and QS proteins in *P. aeruginosa*. Additionally, transcriptomic analysis revealed that the co-expression of *proE* and *pqsE* significantly stimulates the transcription of pyocyanin genes. Our qPCR analysis and pyocyanin quantification assay confirmed the results. Finally, our findings demonstrate that the presence of both *pqsE* and *rhlR* is essential for the promotion of *proE* to pyocyanin production. Hence, our present study suggested that ProE interacts with PqsE to specifically regulate pyocyanin production.

## MATERIALS AND METHODS

### Bacterial strains and growth conditions

The bacterial strains used in this study are detailed in Table S1. *P. aeruginosa* was routinely cultured at 37°C in either Luria–Bertani (LB) medium or *Pseudomonas* isolate agar (PIA) at 37°C. Antibiotics were used at the following concentrations when necessary: gentamicin at 50 µg/mL, carbenicillin at 200 µg/mL for *P. aeruginosa*, ampicillin at 100 µg/mL, gentamicin at 25 µg/mL, and kanamycin at 50 µg/mL for *Escherichia coli*.

### Construction of in-frame deletion and complementary strains

The plasmids and primers used in this study are detailed in Tables S1 and S2. To generate the *pqsE* and *rhlR* deletion mutant of *P. aeruginosa*, two PCR fragments flanking the *pqsE* gene were amplified. The two PCR products were subsequently ligated with the linear vector pK18 which had been digested with EcoRI and BamHI, using the One Step Cloning Kit (Vazyme Biotech, Nanjing, China). The resulting construct was transformed into *E. coli* DH5α competent cells by heat shock at 42°C and then introduced into strain PAO1 through triparental mating. In-frame deletion was performed as described previously (31). The generated *pqsE* deletion mutant was confirmed by PCR and DNA sequencing.

For *in trans* complementation or overexpression, the coding region was amplified using the primers listed in Table S2; the PCR product was cloned into the plasmid pBBR1-MCS5 or pUCP18, which were digested with EcoRI and BamH I. The resultant construct was mobilized into *E. coli* DH5α and sequenced prior to being introduced into the corresponding mutants, followed by confirmation through PCR analysis.

### Immunoprecipitation and mass spectrometry

Immunoprecipitation and mass spectrometry were conducted as previously described with modifications (32). The bacterial cells grown in LB medium overnight were collected and resuspended in lysis buffer (10 mM Tris-Hcl, 150 mM Nacl, 0.5 mM EDTA, 0.5% NP-40, 1 mM PMSF, 1 tablet/20 mL protease inhibitor cocktail tablets [Roche], pH 7.5). The sample was then sonicated and centrifuged at 13,000 × *g* for 20 minutes at 4°C, with the supernatant representing the whole cell lysate. The lysate was incubated with prewashed GFP-Trap (Chromo Tek, gta-20) overnight at 4°C while gently shaking. The resin-protein complex was washed with lysis buffer through three rounds of low-speed centrifugation. For each wash, the resin was gently agitated in the lysis buffer and centrifuged at 3,000 × *g* for 1 minute, and the remaining wash volume was decanted without losing any resin.

The immunoprecipitated proteins were subsequently digested in-solution using trypsin (Sigma-Aldrich, T7409) for 14 hours at 37°C, and the digested peptides were subject to Eksigent nanoLC-Ultra 2D Nano-HPLC/ESI-ion trap-MS/MS analysis with TripleTOF 5600 (AB SCIEX). Raw MS data files were processed and analyzed using PEAKS Studio 8.5 (Bioinformatics Solutions, Inc., Waterloo, Canada). The database search was conducted with the following parameters: Database: UniProt; Taxonomy: *P. aeruginosa*; Enzyme: Trypsin; mass tolerance for primary mass spectrometry: 20 ppm; secondary mass spectrometry: 0.1 Da; false-positive rate of polypeptide: 5% FDR; and a filter by score of >= 20. The sass spectrometry analysis was carried out by Wininnovate Bio Co., Ltd. (China, Shenzhen).

### Bacterial two-hybrid assays

Protein-protein interaction was detected using the BacterioMatch II two-hybrid system (Stratagene) following the manufacturer’s protocol. The constructed plasmids were cotransformed into the reporter strain XL1-Blue MRF’ Kan via electroporation. The positive interaction between ProE and PqsE was confirmed by culturing the cotransformants on an M9^+^ His-deficient plate containing 2.5 mM 3-aminotriazole (3-AT) and incubating at 37°C overnight. Normal growth on the selective screening medium indicates a positive protein-protein interaction.

### Protein cloning, expression, and purification

The experiments were conducted as previously described (31). The DNA fragment encoding *pqsE* and *rhlR* were amplified with the primers listed in Table S2 and subsequently cloned into the expression vector pET28b (+) (Novagen). The resulting plasmid was then transformed into *E. coli* BL21(DE3). For the purification of PqsE, bacteria were grown overnight at 37°C in LB medium until an optical density OD600 of 0.6 was reached. The temperature was then lowered to 18°C, and the culture was continued for an additional 16 hours in the presence of 0.5 mM IPTG. RhlR was purified in the presence of 100 µM mBTL as previously reported (33). The harvested cells were disrupted by sonication, and the proteins were purified using a Ni-NTA column, following the manufacturer’s instructions. The samples were then concentrated and stored at −80°C until use.

### Surface plasmon resonance assay

The binding kinetics of protein-protein interactions were estimated using the surface plasmon resonance (SPR) assay, conducted with a Biacore T200 (GE Healthcare) at 25°C. The running buffer was PBST (0.27 g/L KH_2_PO_4_, 1.42 g/L Na_2_HPO_4_, 8 g/L NaCl, 0.2 g/L KCl, pH 7.4, and 0.005% Tween-20). Following the manufacturer’s protocol, one flow cell of a CM5 sensor chip was activated with a mixture of 0.2 M N-(3-dimethylaminopropyl)-N′-ethylcarbodiimide hydrochloride and 0.05 M N-hydroxysuccinimide in ddH_2_O. The PqsE, prepared in 10 mM sodium acetate (pH 4.5), was injected over the flow cell for 10 minutes at a flow rate of 10 µL/minutes. The remaining binding sites were blocked using 1 M ethanolamine (pH 8.5). A total of 8,000 response units of the PqsE were immobilized. ProE at different concentrations (double dilution) with or without c-di-GMP and metal ion was injected into the immobilized protein and blank flow cells for 5 minutes at a flow rate of 30 µL/minutes. The bound protein was removed with glycine-HCl (pH 4.5). The resulting curves were fitted to either the 1:1 Langmuir binding model or the steady-state affinity model (BIA evaluation 4.1 software) to obtain the equilibrium and kinetic constants.

### Isothermal titration calorimetry assay

Isothermal titration calorimetry (ITC) assay was performed at 25°C using a MicroCal VP-ITC instrument (Malvern Panalytical). Thirty micrometers of ProE protein was titrated with 300 µM PqsE until saturation was reached. Both ProE and PqsE were diluted in the same buffer: PBS supplemented with 5 mM β-mercaptoethanol (BME). There are a total of 20 injections, discarding the first injection when analyzing the data. The data were processed with the MicroCal VP-ITC Analysis software (Malvern Panalytical) and fitted with the “single set of identical sites” model.

### Enzymatic activity assay

Enzyme activity analyses were conducted according to previously described methods (31). In brief, 0.08 µM ProE or PqsE was added to a reaction buffer containing 50 µM c-di-GMP, 100 mM Tris-HCl (pH 8.0), 20 mM KCl, and 5 mM MgCl_2_, resulting in a final volume of 50 µL. The reaction mixture was incubated at 37°C for 20 minutes and then stopped by heating at 95°C for 10 minutes. The PDE activity was evaluated by monitoring the formation of the product 5′-pGpG from the hydrolysis of c-di-GMP using LC-mass spectrometry.

The samples in the glass vials were analyzed using a BEH C18 column (1.7 µm; 2.1 × 50 mm) with an injection volume of 5 µL at a flow rate of 0.3 mL minute^−1^, resulting in a total runtime of 6 minutes. The mobile phase A comprised 10 mM ammonium acetate in water, and mobile phase B comprised 10 mM ammonium acetate in methanol. For the MS portion, the samples were then analyzed by Xevo TQ-S, Waters mass spectrometer, under the ESI-positive ion mode (capillary voltage, 3.8 kV; desolvation temperature, 400°C). The pGpG compound was detected by monitoring the ion transition from 709.31 m/z to 152.26 m/z at a collision energy of 38 eV.

### RNA extraction, sequencing, and transcriptomic analysis

The *P. aeruginosa* strains were cultured in LB broth at 37°C until reaching an OD600 of 2.0. RNA extraction was conducted using the miRNeasy kit (Qiagen) following the manufacturer’s instructions. RNA samples were submitted to the HaploX Genomics Center (Shenzhen, China) for ribosomal RNA depletion and sequencing. The extracted RNA samples were sequenced on an Illumina Hiseq Xten platform, and 150 bp paired-end reads were generated.

The quality of the raw sequence data was assessed using FastQC (Babraham Bioinformatics). Adaptor sequences were removed using the adaptor trimming function in CLC Genomics Workbench 20 (CLC Bio, Aarhus, Denmark). RNA sequence analysis was done using the “RNA-seq analysis” module in CLC using *P. aeruginosa* PAO1 reference genome downloaded from the NCBI database. Differential gene expression was analyzed using the DESeq2 package in R software. Transcripts were stringently determined as diﬀerentially expressed when having a fold change larger than two and an adjusted *P* value smaller than 0.05.

### Quantitative real-time PCR

Quantitative real-time PCR (qRT-PCR) was conducted using a LightCycler 96 Real-Time PCR System (Roche) with ChamQ Universal SYBR qPCR Master Mix (Vazyme Biotech, Nanjing, China) with the following PCR procedure: 95°C for 30 s (95℃, 10 s; 60℃, 30 s) × 40 cycles, 95℃ for 10 s, 60℃ for 60 s, 95℃, 15 s. The experiment was repeated three times, each time with triplicates. The primers used are listed in Table S2. The relative expression levels of the target genes were normalized to the housekeeping gene *rplU,* and the gene expression levels were calculated using the 2^−△△CT^ method (34).

### Reporter assay

For the *P_cdrA_-gfp* and *P_pqsA_-gfp* reporter assays, *P. aeruginosa* strains were cultivated in low phosphate medium (20 g/L pancreatic digest of gelatin [Difco], 1.4 g/L MgCl_2_ [Sigma], and 10 g/L K_2_SO_4_ [Sigma]) (35). The cell culture was diluted into OD600 about 0.01 by corresponding fresh medium and then transferred into each well of a 96-well plate. The OD600 and GFP fluorescence (excitation 485 nm/emission 535 nm) were measured using the Tecan Infinite 200 microplate reader (Tecan, Austria). The relative fluorescence intensity count was calculated by GFP/OD600. Three independent experiments were performed in triplicate, and the results were shown as the mean ± s.d.

### Pyocyanin production

The protocol for this assay was adapted from the previous study (33). Briefly, overnight cultures of *P. aeruginosa* strains grown in LB medium were diluted 1:100 into 5 mL of LB medium and agitated for 16 hours at 37℃. One milliliter of aliquots were removed from the cultures, and cell density (OD600) was measured. The aliquots were then subjected to centrifugation at 16,100 × *g* for 10 minutes, after which the clarified supernatants were removed and filtered through 2 µm filters. The OD695 of each supernatant was measured, and normalized pyocyanin production was determined by calculating OD695/OD600 for each strain. Three independent experiments were performed in triplicate, and the results were shown as the mean ± s.d.

### Biofilm formation

Biofilm formation was performed in 96-well polypropylene microtiter plates according to the previously described method (36). Briefly, the overnight bacterial culture was diluted to OD600nm of 0.001 using fresh LB medium. Two hundred microliters of diluted culture was transferred into 96-well polypropylene microtiter plates and then incubated at 37℃ for 20 hours. The OD600nm of bacterial cultures was determined by a microplate reader (BioTek, United States). The bacterial cells were carefully removed and washed three times with sterile water. The biofilm cells were stained with 100 µL 0.1% crystal violet for 15 min at room temperature. After washing three times, the plates were air-dried at room temperature. For quantification, the bound crystal violet was resuspended in 100 µL of 95% ethanol and measured at 580 nm with a microplate reader (BioTek, United States).

### Swimming motility

The swimming motility assay was carried out as described previously (37). The swimming motility plate contains 10 g/L tryptone, 5 g/L NaCl, and 0.3% agar. Plates were carefully spotted with 2 mL of overnight cultures and incubated at room temperature for 16 hours.

## RESULTS

### Identification of putative ProE binding protein by immunoprecipitation and mass spectrometry

**Fig 1:**
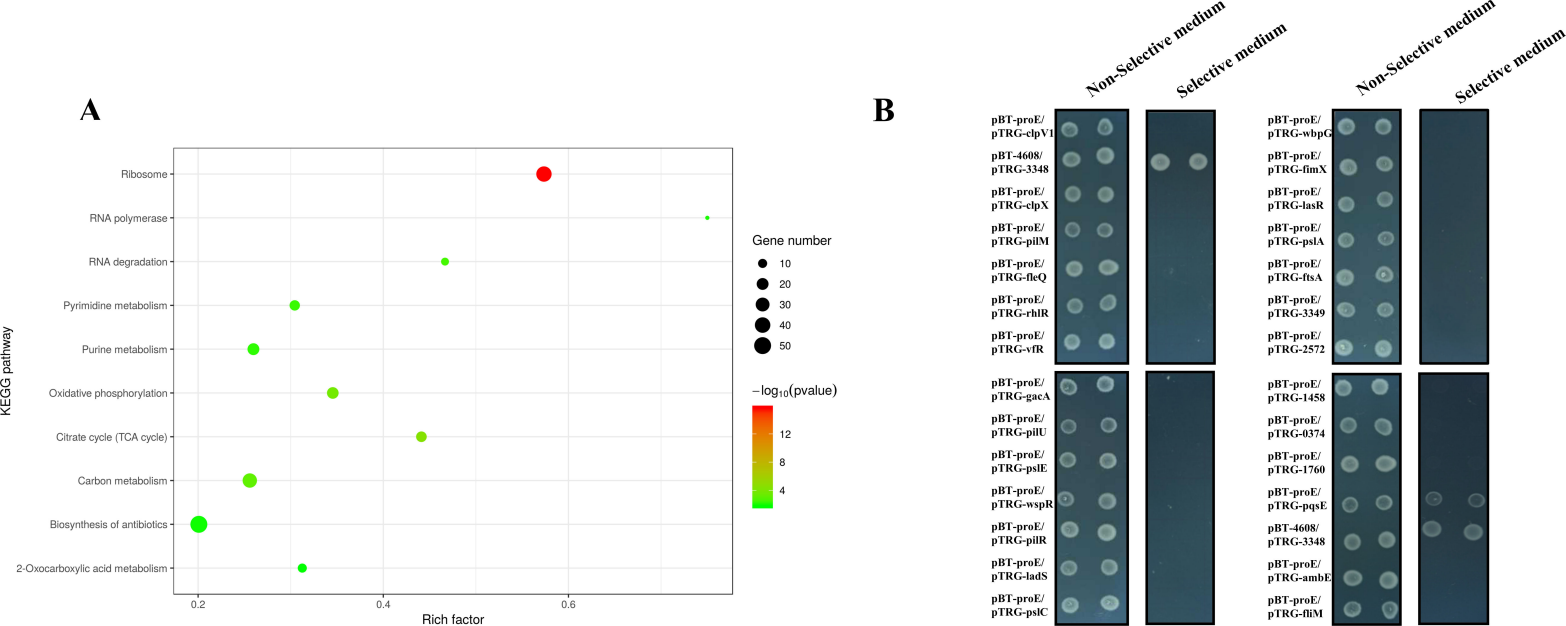
Screening the ProE-interacting proteins by immunoprecipitation-mass spectrometry and bacterial two-hybrid assay. (**A**) KEGG enrichment of putative ProE binding proteins identified by immunoprecipitation-mass spectrometry. (**B**) Bacterial two-hybrid analysis of the protein-protein interaction between ProE and the putative binding proteins.

To identify potential protein partners of ProE in *P. aeruginosa*, we first constructed the pBBR1-MCS5-*proE-gfp* vector, which fuses GFP to the C terminus of ProE, and introduced it into the *proE* deletion mutant; the congo red plate assay indicated that the ProE-GFP fusion protein is functional, as it can restore the colony morphology of the *proE* deletion mutant to that of the wild type (Fig. S1A). The growth curve analysis also suggested that the *proE* deletion mutant harboring pBBR1-MCS5-*proE-gfp* vector exhibited a growth curve similar to that of the wild type (Fig. S1B). Then, we enriched ProE’s interacting proteins according to immunoprecipitation assay via GFP-Trap and identified these proteins by mass spectrometry analysis. The liquid chromatography-tandem mass spectrometry analysis identified a total of 502 proteins, with 1–56 unipeptides per protein (Table S3). The top identified protein was ProE itself, which served as the bait for this affinity assay, with the unipeptide counts of 56 (Table S3). The KEGG analysis indicated that the ProE‐interacting proteins were significantly enriched in various biosynthetic categories (including the ribosome, RNA polymerase, and RNA degradation pyrimidine metabolism) (Fig. 1A). We performed a bacterial two-hybrid assay to verify the direct interaction of ProE with the selected total of 26 putative interacting proteins including EPS/biofilm-associated proteins, QS proteins, and several other types of proteins such as flagellar proteins, type 4 fimbrial biogenesis proteins, and two-component system proteins. Notably, we found that ProE could directly interact with PqsE, a protein that belongs to the PQS quorum sensing system (Fig. 1B).

### PQS quorum sensing protein PqsE can bind to ProE

**Fig 2:**
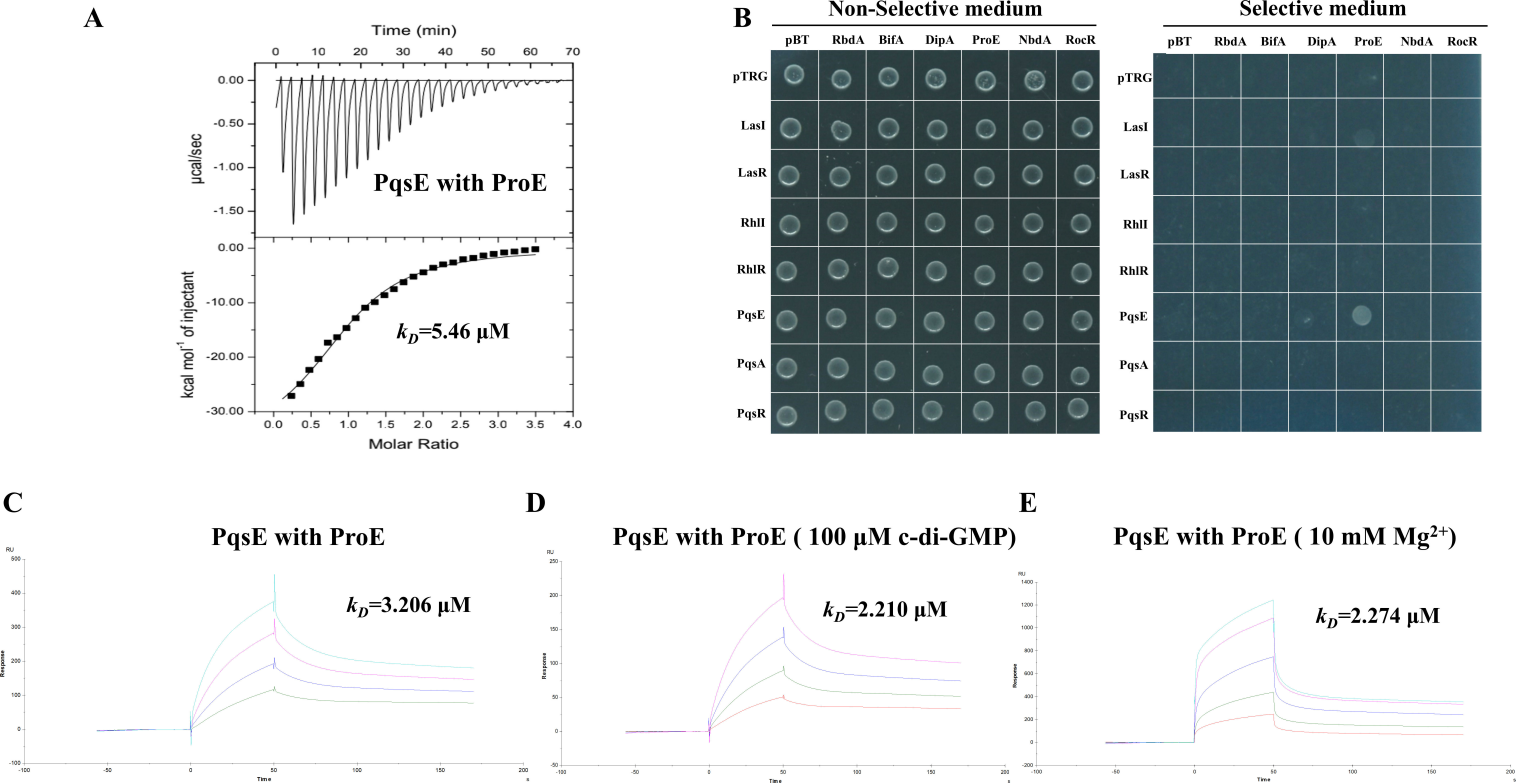
Validation of the ProE-PqsE interaction. (**A**) ITC analysis of the binding of ProE and PqsE interaction. (**B**) Bacterial two-hybrid analysis of the protein-protein interaction between selected PDEs and quorum sensing proteins. (**C to E**) SPR measurements of PqsE binding to ProE and with c-di-GMP or Mg^2+^. Plots are representative of three experiments with similar results. RU, response units; *k_D_*, dissociation constant.

We first confirmed that there is no self-activation effect in our bacterial two-hybrid assay (Fig. S2). Subsequently, we purified ProE and PqsE using His tag affinity chromatography (Fig. S3) and conducted an isothermal titration calorimetry assay; the result showed that ProE bound to PqsE with a dissociation constant (*K_D_*) of 5.46 µM (Fig. 2A), and surface plasmon resonance assay further confirmed the result; the *K_D_* was 3.206 µM (Fig. 2C), which was similar to ITC result. Given that ProE is a functional PDE that can bind c-di-GMP and metal ions, we were curious to know whether c-di-GMP and metal ions can affect the ProE-PqsE interaction. With the addition of 100 µM c-di-GMP or 10 mM Mg^2+^ in ProE-PqsE binding assay by SPR, the measured *K_D_* values are 2.210 µM and 2.274 µM, respectively (Fig. 2D and E), which are comparable to the *K_D_* values observed for the ProE-PqsE interaction. Next, we explored the potential interactions between other PDEs and QS proteins in *P. aeruginosa*; we randomly selected other five previously characterized PDEs including RbdA, BifA, DipA, NbdA, and RocR and six QS proteins including LasI, LasR, RhlI, RhlR, PqsA, and PqsR. By using bacterial two-hybrid assay, we tested the potential interaction between these PDEs and QS proteins. The results indicated that, among the proteins we examined, ProE exhibited a strong interaction with PqsE (Fig. 2B). Although LasI also showed an interaction with ProE, the interaction is too weak to be considered physiologically relevant (Fig. 2B).

### Both *proE* and *pqsE* negatively regulate intracellular c-di-GMP levels

**Fig 3:**
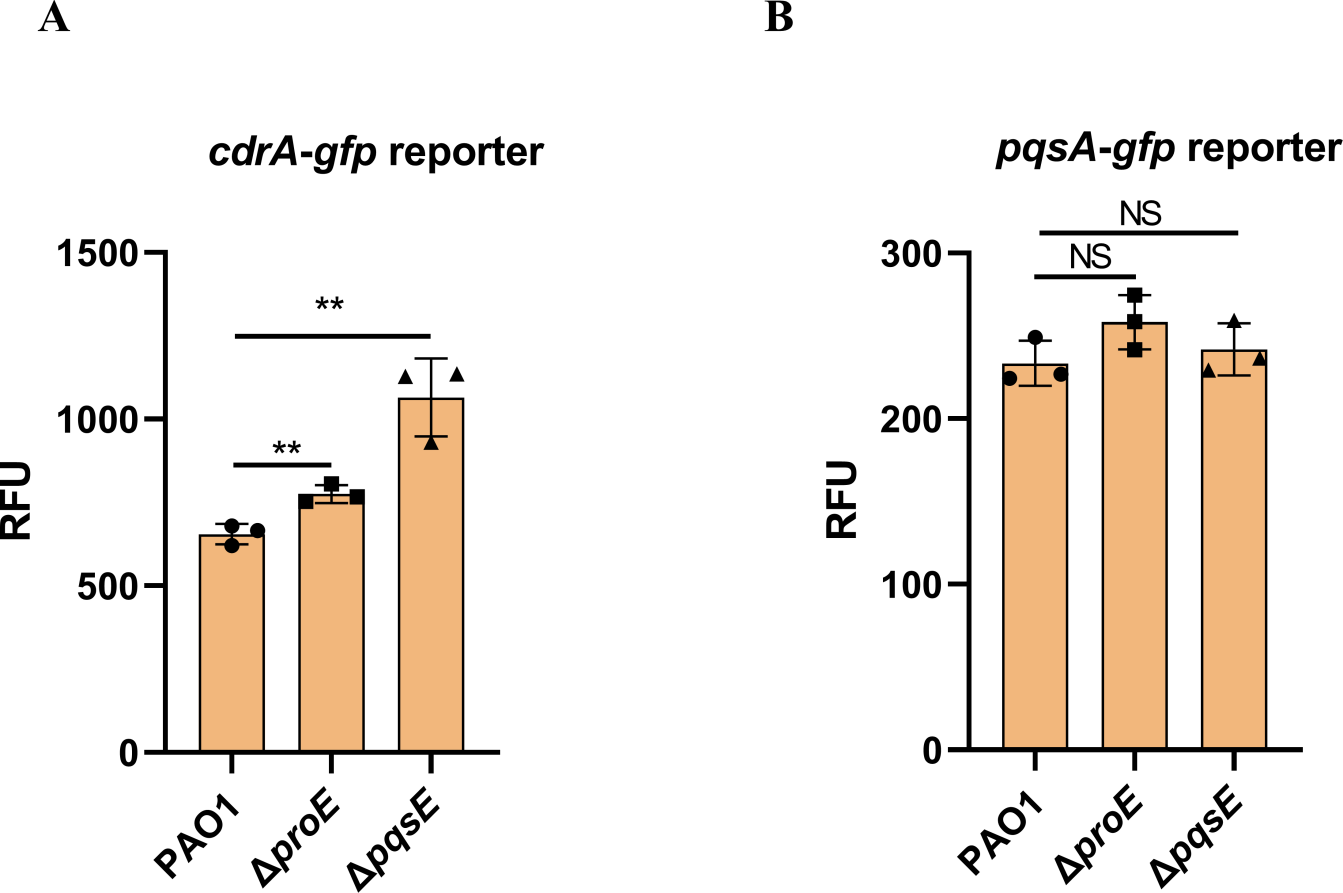
Deletion of either *proE* or *pqsE* showed increased intracellular c-di-GMP levels. Expression of P*_cdrA-_gfp* reporter fusions (**A**) and P*_pqsA-_gfp* reporter fusions (**B**) in wild-type PAO1, Δ*proE* and Δ*pqsE* mutants. Relative fluorescence intensity (reflected as GFP/OD600) was measured in representative strains containing the P*_cdrA_-gfp* or P*_pqsA-_gfp* reporter fusions. Each experiment was performed at least three times in triplicate. ^**^P < 0.01; ns, not significant.

Given that ProE exhibits PDE activity, it can be inferred that the ProE-PqsE interaction might influence intracellular c-di-GMP levels. To evaluate the intracellular c-di-GMP levels, we introduced c-di-GMP reporter systems *P_cdrA_-gfp* plasmid into the wild-type PAO1, Δ*proE* and Δ*pqsE* strains, respectively. Expectedly, the *proE* deletion mutant showed increased intracellular c-di-GMP levels compared to wild-type strain (Fig. 3A). Notably, we also observed that the *pqsE* deletion mutant exhibited increased intracellular c-di-GMP levels (Fig. 3A). We investigated whether PqsE can degrade c-di-GMP, and the result showed that PqsE is unable to hydrolyze c-di-GMP (Fig. S4), which aligns with findings from a previous study (38). The SPR result indicated that PqsE cannot bind to c-di-GMP; in contrast, ProE can bind to c-di-GMP with a *K_D_* value of 2.721 µM (Fig. S5). Furthermore, we confirmed that the deletion of *pqsE* does not affect the transcriptional expression of c-di-GMP metabolic genes, both in our study (data not shown) and other researcher’s studies (39). Collectively, these results suggest that the increased levels of c-di-GMP regulated by PqsE are likely mediated through ProE.

And by using *P_pqsA_-gfp* plasmid, we measured the PQS levels of wild-type PAO1, Δ*proE* and Δ*pqsE* strains; the results indicated that neither *proE* nor *pqsE* influenced the PQS levels (Fig. 3B).

### Comparing the transcriptomes of PAO1, PAO1 harboring either *proE*, *pqsE,* or both

**Fig 4:**
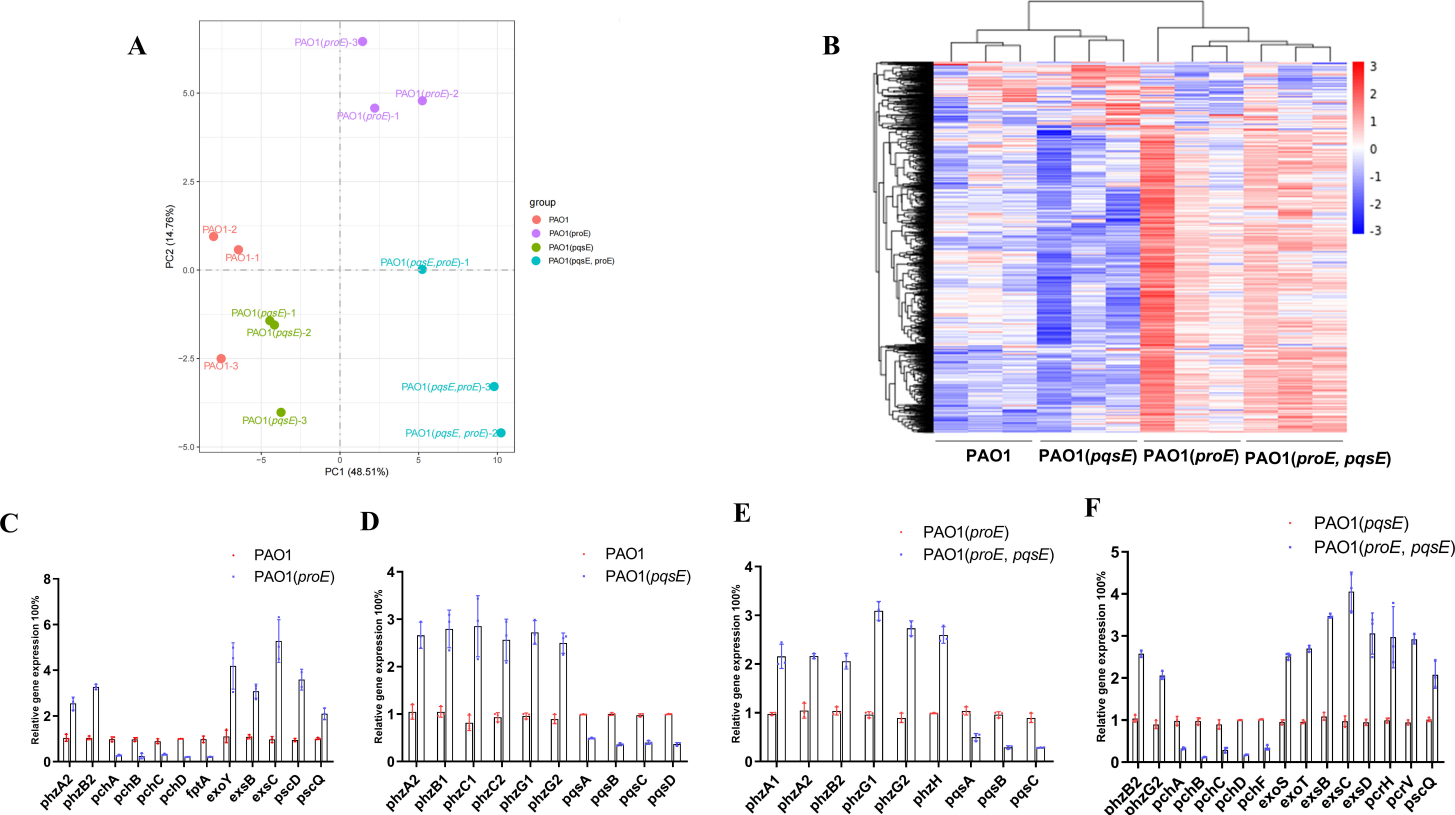
Transcriptomic analysis of *P. aeruginosa* strains harboring either *proE*, *pqsE,* or both. (**A**) PCA shows the expression profiles. Each dot indicates one biological replicate. (**B**) Hierarchical clustering analysis and a heatmap of the differentially expressed genes. (**C**) qPCR validation of the genes differentially expressed in *proE-OE* strain compared to wild-type PAO1, (**D**) genes differentially expressed in *pqsE-OE* strain compared to wild-type PAO1, (**E**) genes differentially expressed in *proE-pqsE-OE* strain compared to *proE-OE* strain, (**F**) genes differentially expressed in *proE-pqsE-OE* strain compared to *pqsE-OE* strain. The experiment was performed at least three times in triplicate. The data presented are the means of replicates, and error bars represent the standard deviation.

In order to explore the biological function regulated by the ProE-PqsE interaction, we constructed *P. aeruginosa* strains expressing either *proE* (designated as *proE-OE*) or *pqsE* (designated as *pqsE-OE*) as well as a strain that simultaneously expressing *proE* and *pqsE* (designated as *proE-pqsE-OE*). This approach mimics the condition in which where *pqsE* and *proE* are constitutively co-expressed and by transcriptomic studies to gain further insights into the ProE-PqsE interaction. Through RNA sequencing, we observed that the four strains (PAO1, *proE-OE*, *pqsE-OE,* and *proE-pqsE-OE*) exhibited distinct gene expression profiles as indicated by the principal component analysis (PCA) and heat map diagram (Fig. 4A and B). Notably, the *proE-OE* strain showed increased expression of pyocyanin genes (*phzA2*, *phzB2*, *phzG1,* and *phzG2*, with log_2_ fold changes ranging from 1.16 to 1.61) and T3SS genes (*exoY*, *exsB*, *exsC*, *pcrG*, *popB*, *popD*, *pscD*, *pscK*, *pscP,* and *pscQ*, with log_2_ fold changes ranging from 1.16 to 1.71) (Table 1; Table S4). In contrast, this strain exhibited decreased expression of siderophore genes (*pchA*, *pchB*, *pchC*, *pchD*, *pchE,* and *fptA*, with log_2_ fold changes ranging from −2.53 to −3.32) when compared to the PAO1 strain (Table 1; Table S4). The *pqsE-OE* strain showed increased expression of pyocyanin genes (*phzA2*, *phzB1*, *phzC1*, *phzC2*, *phzG1*, *phzG2*, *phzH*, and *phzS*, with log_2_ fold changes ranging from 1.12 to 2.01) and with decreased expression of PQS and siderophore genes (*pqsA*, *pqsB*, *pqsC*, *pqsD*, and *pvdP*, with log_2_ fold changes ranging from −1.11 to −1.24) compared to the PAO1 strain (Table 1; Table S5). Notably, the *proE-pqsE*-OE strain demonstrated even greater expression of pyocyanin genes compared to the *proE-OE* strain (*phzA2*, *phzB1*, *phzC1*, *phzC2*, *phzG1*, *phzG2*, *phzH*, and *phzS*, with log_2_ fold changes ranging from 1.16 to 1.98 ) (Table 1; Table S6) or *pqsE-OE* strain (*phzB2*, *phzC2,* and *phzG2*, with log_2_ fold changes ranging from 1.59 to 1.91) (Table 1; Table S7). The coexistence of ProE and PqsE specifically enhances the gene expression of pyocyanin compared to strains harboring either *proE* or *pqsE* alone. To validate the transcriptomic results, we performed qRT-PCR analysis, which confirmed identical gene expression patterns consistent with the transcriptomic data (Fig. 4C through F).

**TABLE 1:** List of selected genes identified in the transcriptomic analyses^*a*^

Gene family	Gene name	PAO1(*proE*) vs PAO1	PAO1(*pqsE*) vs PAO1	PAO1(*proE*, *pqsE*) vs PAO1(*proE*)	PAO1(*proE*, *pqsE*) vs PAO1(*pqsE*)
Pyocyanin	*phzA1*			1.51	
	*phzA2*	1.26	1.76	1.23	
	*phzB1*		1.64	1.27	
	*phzB2*	1.21		1.81	1.82
	*phzC1*		1.24	1.55	
	*phzC2*		1.37	1.98	1.59
	*phzG1*	1.16	2.01	1.68	
	*phzG2*	1.61	1.23	1.52	1.91
	*phzH*		1.12	1.35	
	*phzS*		1.36	1.16	
PQS	*pqsA*		−1.11	−1.56	
	*pqsB*		−1.20	−1.13	
	*pqsC*		−1.36	−1.13	
	*pqsD*		−1.24		
Siderophore	*pchA*	−2.53			−3.44
	*pchB*	−2.68			−3.76
	*pchC*	−2.65			−3.80
	*pchD*	−2.78			−3.82
	*pchE*	−2.88			−3.81
	*pchF*				−3.55
	*pchR*				−1.30
	*fptA*	−3.32			
	*pvdP*		−1.12	−1.04	
T3SS T3SS	*exoS*				1.48
	*exoT*				1.12
	*exoY*	1.23			1.18
	*exsB*	1.16			1.40
	*exsC*	1.25			1.18
	*exsD*				1.32
	*pcrG*	1.57			1.35
	*pcrH*				1.35
	*pcrV*				1.16
	*popB*	1.34			
	*popD*	1.23			
	*pscB*				1.13
	*pscD*	1.20			1.00
	*pscG*				1.73
	*pscI*				1.51
	*pscJ*				1.33
	*pscK*	1.71			
	*pscP*	1.29			
	*pscQ*	1.27			1.03

^*a*^ The numbers indicate the log2 fold change of gene expression.

### Co-expression of *proE* and *pqsE* can enhance the pyocyanin production

**Fig 5:**
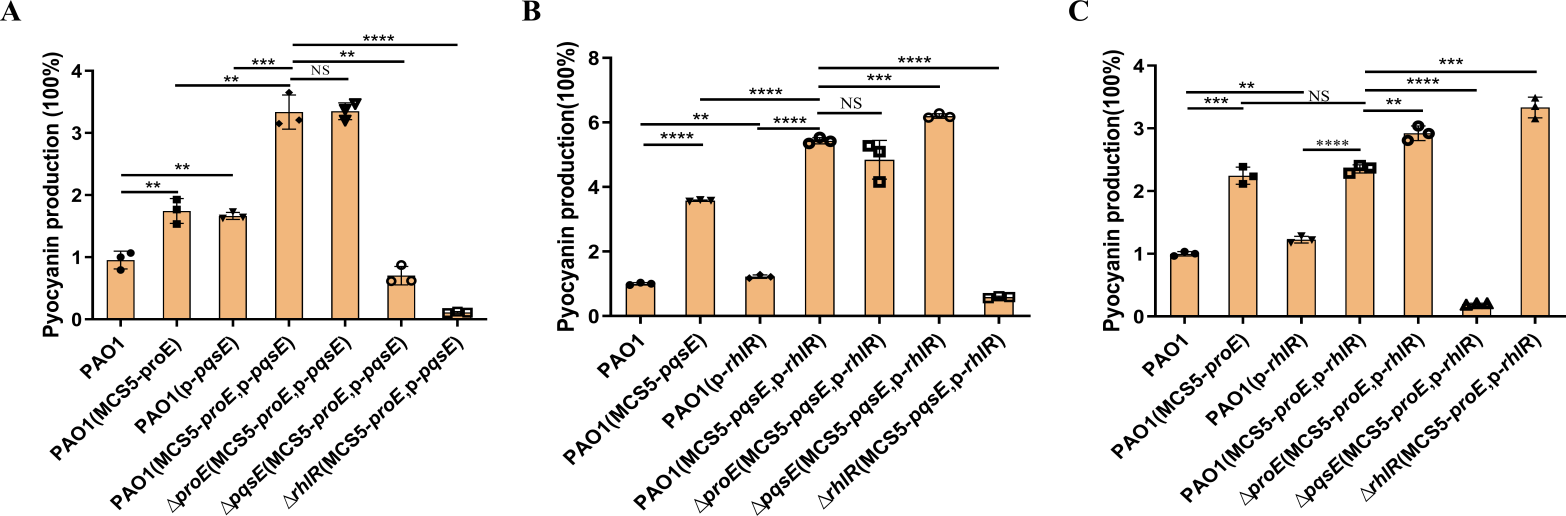
Pyocyanin production measurement by gene co-expression in *P. aeruginosa*. Analysis of the pyocyanin production of *proE-pqsE* co-expression (**A**), *pqsE-rhlR* co-expression (**B**), and *proE-rhlR* co-expression (**C**). Pyocyanin production was measured in the designated *P. aeruginosa* strains and quantified as pigment production (OD695) divided by cell density (OD600). Pyocyanin production of wild-type *P. aeruginosa* was set to 100% to make comparisons. Each experiment was performed at least three times in triplicate. The data presented are the means of replicates, and error bars represent the standard deviation. ^**^P < 0.01, ^***^P < 0.001, ^****^P < 0.0001; ns, not significant.

We first quantitatively assessed the pyocyanin production in the four strains (PAO1, *proE-OE*, *pqsE-OE,* and *proE-pqsE-OE*). The pyocyanin production levels of the *proE-OE* and *pqsE-OE* were found to be 1.7-fold and 1.6-fold higher than that of the PAO1 strain, respectively (Fig. 5A). Significantly, the pyocyanin production of the *proE-pqsE-OE* strain is 1.9-fold and 2.0-fold higher than those of the *proE-OE* or *pqsE-OE* strain, respectively (Fig. 5A), which are consistent with the RNA-seq and qPCR data. And *proE-OE*, *pqsE-OE,* or *proE-pqsE-OE* did not influence the growth of bacteria (Fig. S6). Recently, PqsE was demonstrated to interact with RhlR to regulate pyocyanin production (40, 41). Consistent with these studies (40, 41), we found that the simultaneous overexpression of *pqsE-rhlR* can significantly further increase the pyocyanin production by 1.5 folds and 4.4 folds compared to *pqsE-OE* or *rhlR-OE* alone, respectively (Fig. 5B). We also explore whether the overexpression of *proE-rhlR* could further increase the pyocyanin production; however, the result showed that the *proE-rhlR-OE* strain did not exhibit higher pyocyanin production than *proE-OE* or *rhlR-OE* strains (Fig. 5C). Our findings confirm that *pqsE* and *rhlR* are indispensable for pyocyanin enhancement (Fig. 5). We also explored the influence of ProE-PqsE interaction on bacterial motility and biofilm formation. Unexpectedly, we found that overexpression of both *proE* and *pqsE* increased the biofilm formation. The biofilm formation in the *proE-pqsE-OE* strain is between *proE-OE* and *pqsE-OE* strains. Additionally, RhlR is essential for ProE-PqsE-mediated biofilm formation (Fig. S7A). In contrast, we did not observe a synergistic effect of ProE-PqsE interaction (Fig. S7B).

## DISCUSSION

c-di-GMP plays vital roles in regulating multiple biological functions in bacteria, and the intracellular c-di-GMP levels are controlled by around 41 DGCs and PDEs in *P. aeruginosa*. However, the mechanisms by which these proteins perform their specific functions remain poorly understood. Increasing evidence suggests that protein-protein interaction-mediated regulations play critical role to the specific c-di-GMP signaling processes (17, 18, 21, 30).

In this study, we performed a screening to identify the potential partner of ProE using immunoprecipitation-mass spectrometry and bacterial two-hybrid assay. Our findings indicate that PqsE is a potential partner of ProE (Fig. 1 and 2). The binding affinity of ProE for PqsE was determined to be in the range of *K_D_* 3–5 μM (Fig. 2B); our results showed that c-di-GMP or Mg^2+^ did not change the binding of ProE to PqsE. Additionally, a recent study revealed that the quorum sensing receptor RpfR, which also functions as a PDE, directly interacts with transcription regulator GtrR (*K_D_* of 2.92 µM) to regulate virulence, and the authors found that even high concentration of c-di-GMP (1 mM) was unable to change the binding affinity of RpfR with GtrR by microscale thermophoresis analysis (19). It seems that c-di-GMP plays a dispensable effect when PDE binds with other proteins. Our findings indicate that both ProE and PqsE negatively regulate intracellular c-di-GMP levels (Fig. 3). Biochemical analyses revealed that PqsE neither degrades nor binds to c-di-GMP (Fig. S4 and S5) and does not regulate the expression of c-di-GMP metabolic genes. The increased c-di-GMP levels in *pqsE* deletion mutant are likely according to *proE* (Fig. 3). ProE and PqsE might bind in a certain ratio, and an excess of PqsE will not further enhance ProE activity. Expression of *pqsE* alone did not influence c-di-GMP-regulated swimming motility (Fig. S7B), and previous study has shown that PqsE is a positive regulator of biofilm formation (Fig. S7A) (42). Though *in vitro* enzymatic assay did not show altered activity of ProE by the addition of PqsE or PqsE and RhlR together (Fig. S4), we hypothesize that this discrepancy may be attributed to the difference between *in vivo* and *in vitro* conditions; it is possible that other proteins are involved. Additionally, through reporter assays, we measured the intracellular levels of PQS in the wild-type strain, Δ*proE* and Δ*pqsE* deletion mutants. As expected, both Δ*proE* and Δ*pqsE* exhibited similar PQS levels to those of wild-type PAO1, since *pqsE* was not required for PQS biosynthesis (Fig. 2) (43).

**Fig 6:**
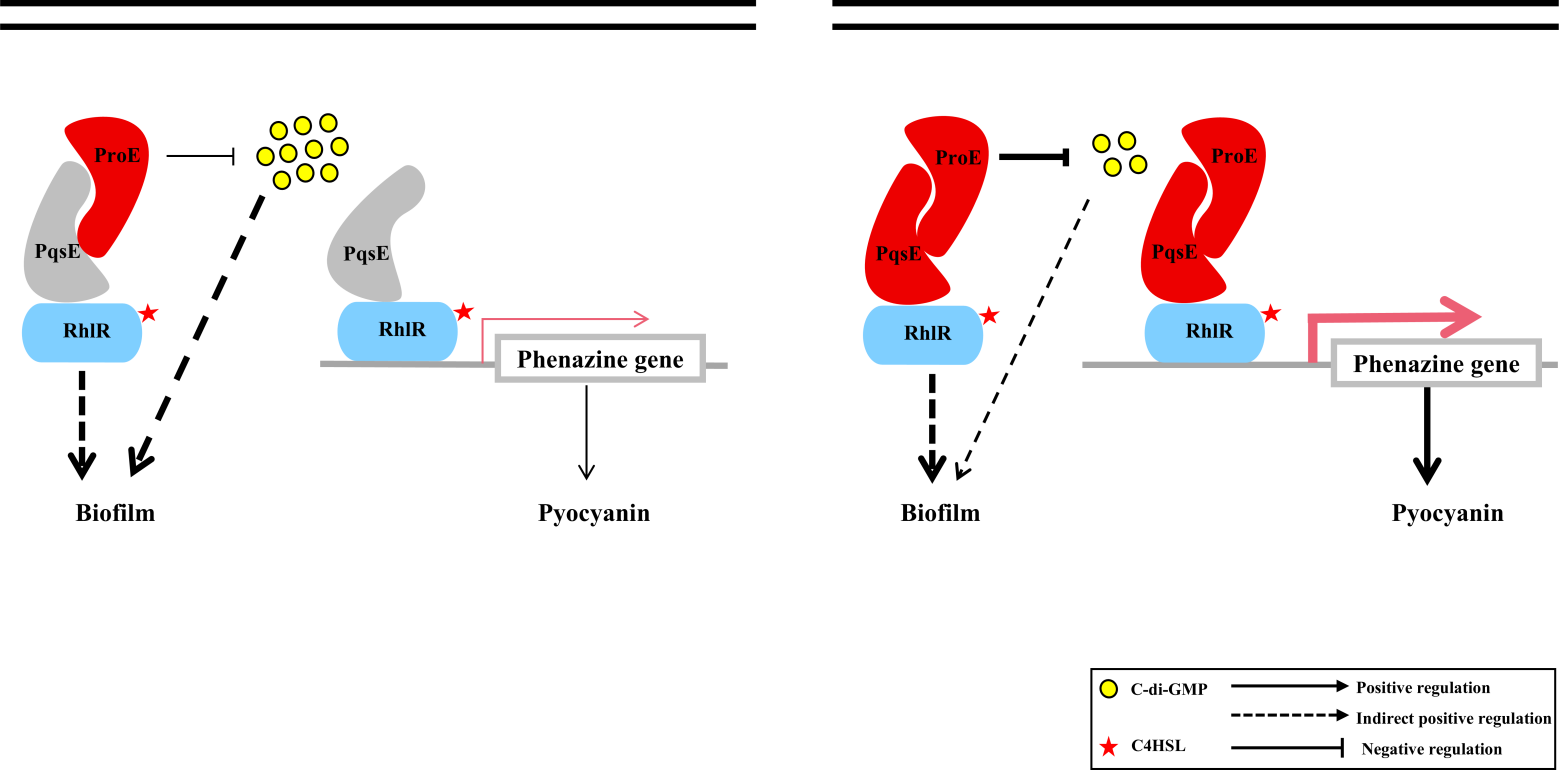
The diagram of ProE-PqsE interaction illustrates the interaction between ProE and PqsE in regulating pyocyanin production and biofilm formation. Under specific conditions, ProE is co-activated for expression with PqsE, which enhances the transcriptional regulation of RhlR and increases pyocyanin production through its interaction with PqsE. When overexpressed alone, ProE serves dual functions: as a PDE and an interaction partner for PqsE, with the latter predominating. Moreover, when ProE and PqsE were co-expressed, the PDE activity of ProE is enhanced by PqsE, and the ability of biofilm formation was between overexpressed ProE and overexpressed PqsE. The thickness of the arrowheads represents the degree of regulation, and the red color represents the overexpression state.

According to our transcriptomic study, we found that the co-expression of *proE* and *pqsE* significantly increases the transcription of pyocyanin genes compared to strain expressing only *proE* or *pqsE* (Table 1), and qPCR and pyocyanin quantification assay confirmed the transcriptome data (Fig. 4 and 5). Recent studies have demonstrated that PqsE functions as a chaperone to interact with RhlR to promote the production of virulence factors (40, 41, 44). Our results confirmed that co-expression of *pqsE* and *rhlR* can significantly enhance pyocyanin production, and when we attempted to co-express *proE* and *rhlR*, we did not observe further increased pyocyanin production. We speculate it might be because ProE cannot directly interact with RhlR (Fig. 2B) and thus unable to regulate its activity. We have also explored the effect of ProE-PqsE interaction on bacterial motility and biofilm formation. Intriguingly, the overexpression of either *proE* or *pqsE* significantly increases biofilm formation (Fig. S7A). ProE may serve two roles when overexpressed alone: functioning as a PDE and acting as a PqsE-interacting partner, which exerts opposing effects on biofilm formation. We propose that the second role predominates when *proE* is overexpressed alone. Our transcriptome analysis did not reveal upregulation of biofilm-associated genes in the *proE*-OE strain compared to the wild-type strain, suggesting that the increase in biofilm formation may occur through post-transcriptional mechanisms. Furthermore, when *proE* and *pqsE* are overexpressed simultaneously, it is possible that ProE’s PDE activity is enhanced by PqsE, which diminishes the biofilm-promoting effect. Consequently, the biofilm formation ability of the *proE-pqsE* overexpression (*proE-pqsE*-OE) strain is intermediate between that of the *proE*-OE and *pqsE*-OE strains (Fig. 6; Fig. S7A). We did not observe the synergistic effect of this interaction on bacterial motility (Fig. S7B).

In our work, we explored the physiological functions regulated by the constitutive co-expression of the ProE-PqsE complex in *P. aeruginosa*. Further investigation is required to identify the signals that promote the expression and formation of the ProE-PqsE protein complex. Elucidating the structural basis of the ProE-PqsE interaction will be beneficial for a deeper understanding of this signaling pathway; however, our attempts to address this issue were unsuccessful due to our failure to obtain a stable ProE-PqsE complex *in vitro*. The role of c-di-GMP is particularly intriguing, as it regulates essential biological functions and is well-conserved in many Gram-negative bacterial species (7). Understanding the signaling mechanisms of c-di-GMP enhances our comprehension of the complex bacterial signal transduction processes. Our study has several implications for the investigation of c-di-GMP signaling. Firstly, we demonstrated that the combination of immunoprecipitation-mass spectrometry and bacterial two-hybrid approaches can effectively identify the potential physiological partner of c-di-GMP-associated DGCs and PDEs. Secondly, our findings underscore the significance of protein-protein interactions mediated by c-di-GMP signaling in regulating specific aspects of bacterial physiology. This mode of action is likely conserved in other bacterial species, and the functional partners of additional DGCs and PDEs, along with the underlying mechanisms, warrant further study.

## Data Availability

The RNA-seq data have been deposited in NCBI under BioProject number PRJNA1162239.

## SUPPLEMENTAL MATERIAL

Supplemental material msphere.01026-24-s0001.docx Fig. S1-S7; Tables S1-S7.

ASM does not own the copyrights to Supplemental Material that may be linked to, or accessed through, an article. The authors have granted ASM a non-exclusive, world-wide license to publish the Supplemental Material files. Please contact the corresponding author directly for reuse.
