# Supplementary material for: c-di-GMP phosphodiesterase ProE interacts with quorum sensing protein PqsE to promote pyocyanin production in Pseudomonas aeruginosa
Source: mSphere. 2025 Jan 28;10(2):e01026-24. doi: 10.1128/msphere.01026-24 (PMC11852716; doi:10.1128/msphere.01026-24)
Supplement: Supplemental material — Fig. S1-S7; Tables S1-S7. [file msphere.01026-24-s0001.docx]

**Supplementary Information**

**Content:**

**Supplementary Table S1**. Bacterial strains and plasmids used in this study.

**Supplementary Table S2.** Primers used in this study.

**Supplementary Table S3.** Proteins identified by liquid chromatography–tandem mass spectrometry (LC-MS/MS).

**Supplementary** **Table S4**. Differential expressed gene in PAO1(*proE*) vs PAO1.

**Supplementary** **Table S5.** Differential expressed gene in PAO1(*pqsE*) vs PAO1

**Supplementary** **Table S6.** Differential expressed genes in PAO1(*pqsE*, *proE*) vs PAO1(*proE*)

**Supplementary** **Table S7**. Differential expressed genes in PAO1(*pqsE*, *proE*) vs PAO1(*pqsE*)

**Supplementary Figure S1.** Congo-red colony morphology and growth assay.

**Supplementary Figure S2.** Testing self-activation by recombinant pBT or pTRG.

**Supplementary Figure S3.** Purification of ProE, PqsE and RhlR:mBTL.

**Supplementary Figure S4.** Measurement of the c-di-GMP hydrolysis activity in vitro.

**Supplementary Figure S5. Measurement of the c-di-GMP binding by SPR**.

**Supplementary Figure S6.** Growth curves of different strains. The data are means of three replicates and error bars indicate standard deviation.

**Supplementary Figure S7. Influence of ProE-PqsE on biofilm formation and swimming motility**.

| **Table S1**. Bacterial strains and plasmids used in this study | | |  |
| --- | --- | --- | --- |
| Strain or plasmid | Relevant characteristics | Reference or source | |
| ***P. aeruginosa* strains** |  |  | |
| PAO1 | Prototrophic laboratory strain | Lab collection | |
| ∆*proE* | *proE* in-frame deletion mutant | (1) | |
| ∆*pqsE* | *pqsE* in-frame deletion mutant | This study | |
| ∆*rhlR* |  | This study | |
| PAO1(MCS5-*proE*) | PAO1 harboring the pBBR1-MCS5-*proE* | This study | |
| PAO1(MCS5-*pqsE*) | PAO1 harboring the pBBR1-MCS5-*pqsE* | This study | |
| PAO1(p-*pqsE*) | PAO1 harboring the pUCP18-*pqsE* | This study | |
| PAO1(p-*rhlR*) | PAO1 harboring the pUCP18-*rhlR* | This study | |
| PAO1(MCS5-*proE*, p-*pqsE*) | PAO1 harboring the pBBR1-MCS5-*proE* and pUCP18-*pqsE* | This study | |
| PAO1 (MCS5-*pqsE*, p-*rhlR*) | PAO1 harboring the pBBR1-MCS5-*pqsE* and pUCP18-*rhlR* | This study | |
| PAO1 (MCS5-*proE*, p-*rhlR*) | PAO1 harboring the pBBR1-MCS5-*proE* and pUCP18-*rhlR* | This study | |
| ∆*proE* (MCS5-*proE*, p-*pqsE*) | ∆*proE* harboring the pBBR1-MCS5-*proE* and pUCP18-*pqsE* | This study | |
| ∆*proE* (MCS5-*proE*, p-*rhlR*) | ∆*proE* harboring the pBBR1-MCS5-*proE* and pUCP18-*rhlR* | This study | |
| ∆*proE* (MCS5-*pqsE*, p-*rhlR*) | ∆*proE* harboring the pBBR1-MCS5-*pqsE* and pUCP18-*rhlR* | This study | |
| ∆*pqsE* (MCS5-*proE*, p-*pqsE*) | ∆*pqsE* harboring the pBBR1-MCS5-*proE* and pUCP18-*pqsE* | This study | |
| ∆*pqsE* (MCS5-*proE*, p-*rhlR*) | ∆*pqsE* harboring the pBBR1-MCS5-*proE* and pUCP18-*rhlR* | This study | |
| ∆*pqsE* (MCS5-*pqsE*, p-*rhlR*) | ∆*pqsE* harboring the pBBR1-MCS5-*pqsE* and pUCP18-*rhlR* | This study | |
| ∆*rhlR* (MCS5-*proE*, p-*pqsE*) | ∆*rhlR* harboring the pBBR1-MCS5-*proE* and pUCP18-*pqsE* | This study | |
| ∆*rhlR* (MCS5-*proE*, p-*rhlR*) | ∆*rhlR* harboring the pBBR1-MCS5-*proE* and pUCP18-*rhlR* | This study | |
| ∆*rhlR* (MCS5-*pqsE*, p-*rhlR*) | ∆*rhlR* harboring the pBBR1-MCS5-*pqsE* and pUCP18-*rhlR* | This study | |
| PAO1(*proE-gfp*) | PAO1 harboring the pBBR1-MCS5-*proE*-*gfp* | This study | |
| PAO1(*cdrA-gfp*) | PAO1 harboring the pUCP22-P*_cdrA_*-*gfp* | (2) | |
| ∆*proE* (*cdrA-gfp*) | ∆*proE* harboring the pUCP22-P*_cdrA_*-*gfp* | This study | |
| ∆*pqsE* (*cdrA-gfp*) | ∆*pqsE* harboring the pUCP22-P*_cdrA_*-*gfp* | This study | |
| PAO1 (*pqsA-gfp*) | PAO1 harboring the pUCP22-P*_pqsA_*-*gfp* | (3) | |
| ∆*proE* (*pqsA-gfp*) | ∆*proE* harboring the pUCP22-P*_pqsA_*-*gfp* | This study | |
| ∆*pqsE* (*pqsA-gfp*) | ∆*pqsE* harboring the pUCP22-P*_pqsA_*-*gfp* | This study | |
| ***E. coli* strains** |  |  | |
| DH5α | F-φ80d lacZΔM15 Δ(lacZYA-argF)U169 endA1 recA1 hsdR17(rk-, mk+) supE44λ-thi -1 gyrA96 relA1 phoA | Lab collection | |
| BL21(DE3) | F- ompT hsdS(rB-mB-) gal dcm(DE3) | TransGen Biotech | |
| XL1-Blue MRF' | Reporter Strain, *Δ(mcrA)183 Δ(mcrCB-hsdSMR-mrr)173*  *endA1 hisB supE44 thi-1 recA1 gyrA96relA1 lac* [F´ lacIq HIS3  aadA Kan^r^] | Stratagene | |
| XL1-Blue MRF'/pBT  /pTRG | XL1-Blue MRF' harboring plasmids pBT and pTRG | This study | |
| XL1-Blue MRF'/pBT-*proE*/pTRG-*pqsE* | XL1-Blue MRF' harboring plasmids pBT-*proE* and pTRG-*pqsE* | This study | |
| XL1-Blue MRF'/pBT  /pTRG-*pqsE* | XL1-Blue MRF' harboring plasmids pBT and pTRG-*pqsE* | This study | |
| XL1-Blue MRF'/pBT-*proE*/pTRG | XL1-Blue MRF' harboring plasmids pBT-*proE* and pTRG | This study | |
| XL1-Blue MRF'/pBT-  *4608*/pTRG-*3348* | XL1-Blue MRF' harboring plasmids pBT-*4608* and pTRG-*3348* | Lab collection | |
| XL1-Blue MRF'/pBT-*rbdA*/pTRG-*lasI* | XL1-Blue MRF' harboring plasmids pBT-*rbdA* and pTRG-*lasI* | This study | |
| XL1-Blue MRF'/pBT-*rbdA*/pTRG-*lasR* | XL1-Blue MRF' harboring plasmids pBT-*rbdA* and pTRG-*lasR* | This study | |
| XL1-Blue MRF'/pBT-*rbdA*/pTRG-*rhlI* | XL1-Blue MRF' harboring plasmids pBT-*rbdA* and pTRG-*rhlI* | This study | |
| XL1-Blue MRF'/pBT-*rbdA*/pTRG-*rhlR* | XL1-Blue MRF' harboring plasmids pBT-*rbdA* and pTRG-*rhlR* | This study | |
| XL1-Blue MRF'/pBT-*rbdA*/pTRG-*pqsA* | XL1-Blue MRF' harboring plasmids pBT-*rbdA* and pTRG-*pqsA* | This study | |
| XL1-Blue MRF'/pBT-*rbdA*/pTRG-*pqsE* | XL1-Blue MRF' harboring plasmids pBT-*rbdA* and pTRG-*pqsE* | This study | |
| XL1-Blue MRF'/pBT-*rbdA*/pTRG-*pqsR* | XL1-Blue MRF' harboring plasmids pBT- *rbdA* and pTRG-*pqsR* | This study | |
| XL1-Blue MRF'/pBT-*bifA*/pTRG-*lasI* | XL1-Blue MRF' harboring plasmids pBT-*bifA* and pTRG-*lasI* | This study | |
| XL1-Blue MRF'/pBT-*bifA* /pTRG-*lasR* | XL1-Blue MRF' harboring plasmids pBT-bifA and pTRG- lasR | This study | |
| XL1-Blue MRF'/pBT-*bifA* /pTRG-*rhlI* | XL1-Blue MRF' harboring plasmids pBT-*bifA* and pTRG-*rhlI* | This study | |
| XL1-Blue MRF'/pBT-*bifA* /pTRG-*rhlR* | XL1-Blue MRF' harboring plasmids pBT-*bifA* and pTRG-*rhlR* | This study | |
| XL1-Blue MRF'/pBT-*bifA* /pTRG-*pqsA* | XL1-Blue MRF' harboring plasmids pBT-*bifA* and pTRG-*pqsA* | This study | |
| XL1-Blue MRF'/pBT-*bifA* /pTRG-*pqsE* | XL1-Blue MRF' harboring plasmids pBT-*bifA* and pTRG-*pqsE* | This study | |
| XL1-Blue MRF'/pBT-*bifA* /pTRG-*pqsR* | XL1-Blue MRF' harboring plasmids pBT-bifA and pTRG-*pqsR* | This study | |
| XL1-Blue MRF'/pBT-*dipA*/pTRG-*lasI* | XL1-Blue MRF' harboring plasmids pBT-*dipA* and pTRG-*lasI* | This study | |
| XL1-Blue MRF'/pBT-*dipA*/pTRG-*lasR* | XL1-Blue MRF' harboring plasmids pBT-dipA and pTRG-*lasR* | This study | |
| XL1-Blue MRF'/pBT-*dipA*/pTRG-*rhlI* | XL1-Blue MRF' harboring plasmids pBT-*dipA* and pTRG-*rhlI* | This study | |
| XL1-Blue MRF'/pBT-*dipA* /pTRG-*rhlR* | XL1-Blue MRF' harboring plasmids pBT-*dipA* and pTRG-*rhlR* | This study | |
| XL1-Blue MRF'/pBT-*dipA*/pTRG-*pqsA* | XL1-Blue MRF' harboring plasmids pBT-*dipA* and pTRG-*pqsA* | This study | |
| XL1-Blue MRF'/pBT-*dipA*/pTRG-*pqsE* | XL1-Blue MRF' harboring plasmids pBT-*dipA* and pTRG-*pqsE* | This study | |
| XL1-Blue MRF'/pBT-*dipA*/pTRG-*pqsR* | XL1-Blue MRF' harboring plasmids pBT-dipA and pTRG- pqsR | This study | |
| XL1-Blue MRF'/pBT-proE/pTRG-lasI | XL1-Blue MRF' harboring plasmids pBT-*proE* and pTRG-*lasI* | This study | |
| XL1-Blue MRF'/pBT-*proE* /pTRG-*lasR* | XL1-Blue MRF' harboring plasmids pBT-*proE* and pTRG-*lasR* | This study | |
| XL1-Blue MRF'/pBT-*proE* /pTRG-*rhlI* | XL1-Blue MRF' harboring plasmids pBT-*proE* and pTRG-*rhlI* | This study | |
| XL1-Blue MRF'/pBT-*proE* /pTRG-*rhlR* | XL1-Blue MRF' harboring plasmids pBT-*proE* and pTRG-*rhlR* | This study | |
| XL1-Blue MRF'/pBT-*proE*/pTRG-*pqsA* | XL1-Blue MRF' harboring plasmids pBT-*proE* and pTRG-*pqsA* | This study | |
| XL1-Blue MRF'/pBT-*proE*/pTRG-*pqsR* | XL1-Blue MRF' harboring plasmids pBT-*proE* and pTRG-*pqsR* | This study | |
| XL1-Blue MRF'/pBT-*nbdA*/pTRG-*lasI* | XL1-Blue MRF' harboring plasmids pBT-*nbdA* and pTRG-*lasI* | This study | |
| XL1-Blue MRF'/pBT-*nbdA*/pTRG-*lasR* | XL1-Blue MRF' harboring plasmids pBT-*nbdA* and pTRG-*lasR* | This study | |
| XL1-Blue MRF'/pBT-*nbdA*/pTRG-*rhlI* | XL1-Blue MRF' harboring plasmids pBT-*nbdA* and pTRG-*rhlI* | This study | |
| XL1-Blue MRF'/pBT-*nbdA*/pTRG-*rhlR* | XL1-Blue MRF' harboring plasmids pBT-*nbdA* and pTRG-*rhlR* | This study | |
| XL1-Blue MRF'/pBT-*nbdA*/pTRG-*pqsA* | XL1-Blue MRF' harboring plasmids pBT-*nbdA* and pTRG-*pqsA* | This study | |
| XL1-Blue MRF'/pBT- *nbdA* /pTRG-*pqsE* | XL1-Blue MRF' harboring plasmids pBT-*nbdA* and pTRG-*pqsE* | This study | |
| XL1-Blue MRF'/pBT-*nbdA*/pTRG-*pqsR* | XL1-Blue MRF' harboring plasmids pBT-*nbdA* and pTRG-*pqsR* | This study | |
| XL1-Blue MRF'/pBT-*rocR*/pTRG-*lasI* | XL1-Blue MRF' harboring plasmids pBT-*rocR* and pTRG-*lasI* | This study | |
| XL1-Blue MRF'/pBT-*rocR*/pTRG-*lasR* | XL1-Blue MRF' harboring plasmids pBT-*rocR* and pTRG-*lasR* | This study | |
| XL1-Blue MRF'/pBT-*rocR*/pTRG-*rhlI* | XL1-Blue MRF' harboring plasmids pBT-*rocR* and pTRG-*rhlI* | This study | |
| XL1-Blue MRF'/pBT-*rocR*/pTRG-*rhlR* | XL1-Blue MRF' harboring plasmids pBT-*rocR* and pTRG-*rhlR* | This study | |
| XL1-Blue MRF'/pBT-*rocR*/pTRG-*pqsA* | XL1-Blue MRF' harboring plasmids pBT-*rocR* and pTRG-*pqsA* | This study | |
| XL1-Blue MRF'/pBT-*rocR*/pTRG-*pqsE* | XL1-Blue MRF' harboring plasmids pBT-*rocR* and pTRG-*pqsE* | This study | |
| XL1-Blue MRF'/pBT-*rocR*/pTRG-*pqsR* | XL1-Blue MRF' harboring plasmids pBT-*rocR* and pTRG-*pqsR* | This study | |
| **Plasmid** |  |  | |
| pK18mobsacB | Broad-host-range gene replacement vector, sacB, Gm^r^ | Lab collection | |
| pK18-*pqsE* | pK18 containing fragments flanking *pqsE* coding sequence fragment; Gm^r^ | This study | |
| pK18-*rhlR* | pK18 containing fragments flanking *rhlR* coding sequence fragment; Gm^r^ | This study | |
| pBBR1-MCS5 | Broad-host-range expression vector, Gm^r^ | (4) | |
| pBBR1-MCS5-*proE* | *proE* cloned in pBBR1MCS under its native promoter, Gm^r^ | (1) | |
| pBBR1-MCS5-*proE*-gfp | *proE-gfp* cloned in pBBR1MCS under its native promoter, Gm^r^ | This study | |
| pBBR1-MCS5-*pqsE* | *pqsE* cloned in pBBR1MCS under its native promoter, Gm^r^ | This study | |
| pUCP18-*pqsE* | *pqsE* cloned in pUCP18, Gm^r^; Carb^r^ | This study | |
| pUCP18-*rhlR* | *rhlR* cloned in pUCP18, Gm^r^; Carb^r^ | This study | |
| pUCP22-P*cdrA*-*gfp* | pUCP22 carrying the *cdrA*-*gfp* transcriptional fusion, Gm^r^; Carb^r^ | (5) | |
| pUCP22-P*pqsA*-*gfp* | pUCP22 carrying the *pqsA*-*gfp* transcriptional fusion, Gm^r^; Carb^r^ | (3) | |
| pRK2013 | RK2 derivative, *mob*^+^ *tra*^+^ *ori* ColE1; Kan^r^ | (6) | |
| pBT | Two-hybrid system bait plasmid containing the *cat* gene, p15A origin of replication and λ cI ORF, Cml^r^ | Stratagene | |
| pTRG | Two-hybrid system target plasmid containing the tet gene,  ColE1 origin of replication, and RNA polymerase α subunit  ORF, Tc^r^ | Stratagene | |
| pBT-*proE* | pBT containing *proE* | This study | |
| pET28b | His-tag protein expression vector, Kan^r^ | Novagen | |
| pET28b-*proE* | pET28b containing *proE* | This study | |
| pET28b-*pqsE* | pET28b containing *pqsE* | This study | |
| pET28b-*rhlR* | pET28b containing *rhlR* | This study | |
| pBT-*rbdA* | pBT containing *rbdA* | This study | |
| pBT-*bifA* | pBT containing *bifA* | This study | |
| pBT-*dipA* | pBT containing *dipA* | This study | |
| pBT-*nbdA* | pBT containing *nbdA* | This study | |
| pBT-*rocR* | pBT containing *rocR* | This study | |
| pTRG-*lasI* | pTRG containing *lasI* | This study | |
| pTRG-*lasR* | pTRG containing *lasR* | This study | |
| pTRG-*rhlI* | pTRG containing *rhlI* | This study | |
| pTRG-*rhlR* | pTRG containing *rhlR* | This study | |
| pTRG-*pqsA* | pTRG containing *pqsA* | This study | |
| pTRG-*pqsR* | pTRG containing *pqsR* | This study | |
| pTRG-*pqsE* | pTRG containing *pqsE* | This study | |

*Symbol: Gm^r^, gentamicin resistant; Carbr , carbenicillin resistant ; Cml^r^, chloramphenicol resistant; Tc^r^, tetracycline resistant; Kan^r^, kanamycin resistant.

**Table S2.** PCR primers used in this study

| Primer | Sequence (5’-3’) |
| --- | --- |
| For in-frame deletion |  |
| *pqsE*-Up-F | ctatgacatgattacgaattcAGCGCCGGCGAGAGTCTC |
| *pqsE*-Up-R | atgtcccgtcGGCCGGTTCACCTCCTCA |
| *pqsE*-Down-F | tgaaccggccGACGGGACATCCATTGCGG |
| *pqsE*-Down-R | caggtcgactctagaggatccAGGCTGGACAGGCCATGC |
| *rhlR*-Up-F | ctatgacatgattacgaattcGCGGTGCGCCGCAAGGTC |
| *rhlR* -Up-R | cgcttcagatgagATTCCTCATTGCAGTAAGCCCTG |
| *rhlR*-Down-F | gaggaatCTCATCTGAAGCGCAGGGC |
| *rhlR*-Down-R | caggtcgactctagaggatccACGGCTGACGACCTCACACC |
| For *in trans* complementation |  |
| pBBR1-MCS5-*pqsE*-F | gataagcttgatatcgaattcATGTTGAGGCTTTCGGCTCC |
| pBBR1-MCS5-*pqsE*-R | cgctctagaactagtggatccTCAGTCCAGAGGCAGCGC |
| pUCP18-*pqsE*-F | tatgaccatgattacgaattcATGTTGAGGCTTTCGGCTCC |
| pUCP18-*pqsE*-R | caggtcgactctagaggatccTCAGTCCAGAGGCAGCGC |
| pUCP18-*rhlR*-F | tatgaccatgattacgaattcATGAGGAATGACGGAGGCTTT |
| pUCP18-*rhlR*-R | caggtcgactctagaggatccTCAGATGAGACCCAGCGCC |
| For protein expression |  |
| pET28b-*pqsE*-F | gtgccgcgcggcagccatatgATGTTGAGGCTTTCGGCTCC |
| pET28b-*pqsE*-R | gtggtggtggtggtgctcgagTCAGTCCAGAGGCAGCGC |
| pET28b-*rhlR*-F | gtgccgcgcggcagccatatgATGAGGAATGACGGAGGCTTT |
| pET28b-*rhlR*-R | gtggtggtggtggtgctcgagTCAGATGAGACCCAGCGCC |
| For bacterial two-hybrid |  |
| pBT-*rbdA*-F | gaagagacgtttggcgcggccgcAATGAGGCAGAACCGGACTCT |
| pBT-*rbdA*-R | aattaattaactcgaggatccCTACCGGAGGTTCTGTCCCAG |
| pBT-*bifA*-F | gaagagacgtttggcgcggccgcATTGAAACTGGACTCCCGACAC |
| pBT-*bifA*-R | aattaattaactcgaggatccTCAGGGCCGTTCGCTGCT |
| pBT-*dipA*-F | gaagagacgtttggcgcggccgcAATGAAAAGTCATCCCGATGCC |
| pBT-*dipA*-R | aattaattaactcgaggatccTCAGTGCAGGGTGCGGCA |
| pBT-*nbdA*-F | gaagagacgtttggcgcggccgcAATGCCTTTTCTCCCCGGG |
| pBT-*nbdA*-R | aattaattaactcgaggatccTCAGGCCTGGTTCAGGCTG |
| pBT-*rocR*-F | gaagagacgtttggcgcggccgcAATGATGAAAACCCATCCGCT |
| pBT-*rocR*-R | aattaattaactcgaggatccTCAGAAGTAGTAGCCGATGTTCAGG |
| pTRG-*lasI*-F | aaaccagaggcggccggatccATGATCGTACAAATTGGTCGGC |
| pTRG-*lasI*-R | gcgccagctcagactgaattcTCATGAAACCGCCAGTCGC |
| pTRG-*lasR*-F | aaaccagaggcggccggatccATGGCCTTGGTTGACGGTTT |
| pTRG-*lasR*-R | gcgccagctcagactgaattcTCAGAGAGTAATAAGACCCAAATTAACG |
| pTRG-*rhlI*-F | aaaccagaggcggccggatccATGATCGAATTGCTCTCTGAATCG |
| pTRG-*rhlI*-R | gcgccagctcagactgaattcTCACACCGCCATCGACAGC |
| pTRG-*rhlR*-F | aaaccagaggcggccggatccATGAGGAATGACGGAGGCTTT |
| pTRG-*rhlR*-R | gcgccagctcagactgaattcTCAGATGAGACCCAGCGCC |
| pTRG-*pqsA*-F | aaaccagaggcggccggatccATGTCCACATTGGCCAACCTG |
| pTRG-*pqsA*-R | gcgccagctcagactgaattcTCAACATGCCCGTTCCTCC |
| pTRG-*pqsR*-F | aaaccagaggcggccggatccATGCCTATTCATAACCTGAATCACG |
| pTRG-*pqsR*-R | gcgccagctcagactgaattcCTACTCTGGTGCGGCGCG |
| pTRG-*pqsE*-F | aaaccagaggcggccggatccATGTTGAGGCTTTCGGCTCC |
| pTRG-*pqsE*-R | gcgccagctcagactgaattcTCAGTCCAGAGGCAGCGC |
| pTRG-*clpX*-F | aaaccagaggcggccggatccATGACTGATACCCGCAACGG |
| pTRG-*clpX*-R | gcgccagctcagactgaattcTCATGCCTCAGGCGCAGC |
| pTRG-*pilM*-F | aaaccagaggcggccggatccGTGCTAGGGCTCATAAAGAAGAAAG |
| pTRG-*pilM*-R | gcgccagctcagactgaattcTCAGTCGAAACTCCTCAACGC |
| pTRG-*fleQ*-F | aaaccagaggcggccggatccATGTGGCGCGAAACCAAA |
| pTRG-*fleQ*-R | gcgccagctcagactgaattcTCAATCATCCGACAGGTCGTC |
| pTRG-*vfR*-F | aaaccagaggcggccggatccATGGTAGCTATTACCCACACACCC |
| pTRG-*vfR*-R | gcgccagctcagactgaattcTCAGCGGGTGCCGAAGAC |
| pTRG-*gacA*-F | aaaccagaggcggccggatccGTGATTAAGGTGCTGGTGGTCG |
| pTRG-*gacA*-R | gcgccagctcagactgaattcCTAGCTGGCGGCATCGACC |
| pTRG-*pilU*-F | aaaccagaggcggccggatccATGGAATTCGAAAAGCTGCTGC |
| pTRG-*pilU*-R | gcgccagctcagactgaattcTCAGCGGAAGCGCCGGCC |
| pTRG-*pslE*-F | aaaccagaggcggccggatccATGATAGAAATTCGTTCCTTGCG |
| pTRG-*pslE*-R | gcgccagctcagactgaattcTCAGAACGCGCTCCGGTA |
| pTRG-*wspR*-F | aaaccagaggcggccggatccATGCACAACCCTCATGAGAGCA |
| pTRG-*wspR*-R | gcgccagctcagactgaattcTCAGCCCGCCGGGGCCGG |
| pTRG-*pilR*-F | aaaccagaggcggccggatccATGAGCCGACAAAAAGCCC |
| pTRG-*pilR*-R | gcgccagctcagactgaattcTCAGTCGATGCCCAGCTTTT |
| pTRG-*ladS*-F | aaaccagaggcggccggatccATGCGGCACTGGCTGATTC |
| pTRG-*ladS*-R | gcgccagctcagactgaattcTCAGGCGGACTTGGTGACG |
| pTRG-*pslC*-F | aaaccagaggcggccggatccATGCGCTGCGCCCTGGTC |
| pTRG-*pslC*-R | gcgccagctcagactgaattcTCACTTCCAGTAGCCTGGAAACA |
| pTRG-*wbpG*-F | aaaccagaggcggccggatccGTGATGAAGATCTGTTCGCGC |
| pTRG-*wbpG*-R | gcgccagctcagactgaattcTCACGAGTAGACATTGATGCGAC |
| pTRG-*fimX*-F | aaaccagaggcggccggatccATGGCCATCGAAAAGAAAACC |
| pTRG-*fimX*-R | gcgccagctcagactgaattcTCATTCGTCTCCCGAGGAGA |
| pTRG-*pslA*-F | aaaccagaggcggccggatccATGCATTCGAAGTCGGTAGATAGC |
| pTRG-*pslA*-R | gcgccagctcagactgaattcTCAGTAGACTTCCTTGGTCAGGAGT |
| pTRG-*ftsA*-F | aaaccagaggcggccggatccATGGCAAGCGTGCAGAGC |
| pTRG-*ftsA*-R | gcgccagctcagactgaattcTCAGAAATTGCCCTGGACCC |
| pTRG-*3349*-F | aaaccagaggcggccggatccATGGCCGGTGTTTTGGATTC |
| pTRG-*3349*-R | gcgccagctcagactgaattcTCAGCCCGCGTCCACCGC |
| pTRG-*2572*-F | aaaccagaggcggccggatccATGAACGATAGCGCACCTCC |
| pTRG-*2572*-R | gcgccagctcagactgaattcCTAGGTCGTCGACTCCGGG |
| pTRG-*1458*-F | aaaccagaggcggccggatccATGAGCTTCGACGCCGATG |
| pTRG-*1458*-R | gcgccagctcagactgaattcTCAGATGCGCCGTGCGTA |
| pTRG-*0374*-F | aaaccagaggcggccggatccATGATCCGCTTCGAGCAGG |
| pTRG-*0374*-R | gcgccagctcagactgaattcTCAGGCCTCATCCTCACGG |
| pTRG-*1760*-F | aaaccagaggcggccggatccATGGGCCATCGCGACGGT |
| pTRG-*1760*-R | gcgccagctcagactgaattcTCACGCCTCGCTCAGCAG |
| pTRG-*ambE*-F | aaaccagaggcggccggatccATGAGTGCGTCAGAAGACCTGC |
| pTRG-*ambE*-R | gcgccagctcagactgaattcTCAGGTTGCCAGGTTCGCC |
| pTRG-*fliM*-F | aaaccagaggcggccggatccATGGCCGTGCAAGATCTGC |
| pTRG-*fliM*-R | gcgccagctcagactgaattcTCAGCGCGAGCGCTCGAC |
| For qRT-PCR |  |
| *phzA1*-F | TCAGCGGTACAGGGAAACAC |
| *phzA1*-R | GAAGTGGTTCGGATCCTCGG |
| *phzA2*-F | GACAACCTGGAATTGCGTCG |
| *phzA2*-R | GAAGTGGTTCGGATCCTCGG |
| *phzB1*-F | TCCCGATTGGGAGTGGTACA |
| *phzB1*-R | TTCATGAATTCGCGGTTGCG |
| *phzB2*-F | GCGAGACGGTGGTCAAGTAT |
| *phzB2*-R | GCTTGTCCTTGCCACGAATG |
| *phzC1*-F | GGATCCTCAAGGGCTATGCG |
| *phzC1*-R | CAGCATCGACAGCTCGTAGT |
| *phzC2*-F | GGATCCTCAAGGGCTATGCG |
| *phzC2*-R | CAGCATCGACAGCTCGTAGT |
| *phzG1*-F | AGCCAGCAGATCATCCTCAAC |
| *phzG1*-R | GATCGACATCGGGTGGGTC |
| *phzG2*-F | AGCCAGCAGATCATCCTCAAC |
| *phzG2*-R | GATCGACATCGGGTGGGTC |
| *phzH*-F | TCTTCGCCATGACCGATACG |
| *phzH*-R | TAGACGAGGGTGACCTCCTG |
| *pqsA*-F | CTCGACGATTTCTCGCTGGA |
| *pqsA*-R | TGGAACCCGAGGTGTATTGC |
| *pqsB*-F | ACGTCAAGGGACACCTCAAC |
| *pqsB*-R | CAGCAGTTCATCCAGACGGT |
| *pqsC*-F | TCCATCCCGTACACCCTGAT |
| *pqsC*-R | CTCACCCAATTGCCAGACCT |
| *pqsD*-F | GAGTCTCGAAGACGGACTGC |
| *pqsD-*R | TCGGTTGATGGCAGATCACA |
| *pchA*-F | CGAGATCGACCCATTGGACC |
| *pchA*-R | TGCCAGTTTTCCTCGATCCG |
| *pchB*-F | AAACTCCCGAAGACTGCACC |
| *pchB*-R | AGTACTTGATCTGCTCGGCG |
| *pchC*-F | CGACTACCAGGCGATCGAG |
| *pchC*-R | AAAGGGCGTCGGGATGTG |
| *pchD*-F | CTTCGTCGAGACCTGCTTCG |
| *pchD*-R | GAAGCGACAGAAACCGCTGA |
| *pchF*-F | CATCACCCTGTGGAACTCGG |
| *pchF*-R | AATCCAGTCACCGGACCAGA |
| *pchR*-F | CAGCGTATTCGGCTACCTGC |
| *pchR*-R | CGGAAAGCGATGGAGAAATGC |
| *fptA*-F | TCAAGGACAGCCAGAACGAC |
| *fptA*-R | CGGTAGTCGACGCTGTAGTC |
| *pvdR*-F | GCGGATCGAGATGTACCAGG |
| *pvdR*-F | GCTGAGGGTCGTGAAGTAGG |
| *exoY*-F | GGTGGTTTCTAACGCAACGG |
| *exoY*-R | GCTTTTCCCCTTCACCGAGA |
| *exsB*-F | TCAGGGAAGGATGCAAACGAT |
| *exsB*-R | GGTACAATGCCTCGCCCTC |
| *exsC*-F | TAACGAGCAAGGTCAACCGA |
| *exsC*-R | AATGCAGATCGAAACGGTGC |
| *pscD*-F | GTGCCCTATGTAGTGCTCGG |
| *pscD*-R | GCGTTTCCAAGGCTGTCATC |
| *pscQ*-F | GAACTCGACCAACTGCCGA |
| *pscQ*-R | CCAGTTCGCCGATACCGAG |
| *exoS*-F | ATCGACCAAGGTATGTCCGC |
| *exoS*-R | CCGAACACGGTGGATATCGT |
| *exoT*-F | ATAACCACCCTGTTCGGCAG |
| *exoT*-R | TCCTTGGCACTGAGAAGCAC |
| *pcrH*-F | CTGTGCATGCTCGACCACTA |
| *pcrH*-R | GAGTAGAAGCCACTCTCGGC |
| *pcrV*-F | TCCTGGTGTCGGCCTATTTC |
| *pcrV*-R | CGACTGGATCACGCTGTAGA |
| *pscJ*-F | ATGAGGCGAACGGTGAAAGG |
| *pscJ*-R | CTTCCTTCTGGCTGATCCCG |
| *pscK*-F | ATTGACGGCCTACCAGTTGC |
| *pscK*-R | GCAGTCCAATTCCAGTTGCTC |
| *rplU*-F | GCAGCACAAAGTCACCGAAG |
| *rplU*-R | CCGATTTTCACGTCTTCGCC |

Table S3 Proteins identified by liquid chromatography–tandem mass spectrometry (LC-MS/MS)

| Accession | Protein Description | -10lgP |
| --- | --- | --- |
| tr\|Q9HTQ9\|Q9HTQ9_PSEAE | Uncharacterized protein OS=Pseudomonas aeruginosa (strain ATCC 15692 / DSM 22644 / CIP 104116 / JCM 14847 / LMG 12228 / 1C / PRS 101 / PAO1) GN=PA5295 PE=4 SV=1 | 387.78 |
| Q9I2V5\|ACNB_PSEAE | Aconitate hydratase B OS=Pseudomonas aeruginosa (strain ATCC 15692 / DSM 22644 / CIP 104116 / JCM 14847 / LMG 12228 / 1C / PRS 101 / PAO1) GN=acnB PE=3 SV=1 | 365.38 |
| P30718\|CH60_PSEAE | 60 kDa chaperonin OS=Pseudomonas aeruginosa (strain ATCC 15692 / DSM 22644 / CIP 104116 / JCM 14847 / LMG 12228 / 1C / PRS 101 / PAO1) GN=groL PE=1 SV=3 | 356.04 |
| P09591\|EFTU_PSEAE | Elongation factor Tu OS=Pseudomonas aeruginosa (strain ATCC 15692 / DSM 22644 / CIP 104116 / JCM 14847 / LMG 12228 / 1C / PRS 101 / PAO1) GN=tufA PE=1 SV=2 | 351.6 |
| Q9HZE0\|DHE2_PSEAE | NAD-specific glutamate dehydrogenase OS=Pseudomonas aeruginosa (strain ATCC 15692 / DSM 22644 / CIP 104116 / JCM 14847 / LMG 12228 / 1C / PRS 101 / PAO1) GN=gdhB PE=1 SV=1 | 350.7 |
| Q9I742\|CLPV1_PSEAE | Protein ClpV1 OS=Pseudomonas aeruginosa (strain ATCC 15692 / DSM 22644 / CIP 104116 / JCM 14847 / LMG 12228 / 1C / PRS 101 / PAO1) GN=clpV1 PE=1 SV=1 | 314.25 |
| Q9I0J6\|NUOG_PSEAE | NADH-quinone oxidoreductase subunit G OS=Pseudomonas aeruginosa (strain ATCC 15692 / DSM 22644 / CIP 104116 / JCM 14847 / LMG 12228 / 1C / PRS 101 / PAO1) GN=nuoG PE=3 SV=1 | 311.02 |
| tr\|Q9I1H3\|Q9I1H3_PSEAE | AmbE OS=Pseudomonas aeruginosa (strain ATCC 15692 / DSM 22644 / CIP 104116 / JCM 14847 / LMG 12228 / 1C / PRS 101 / PAO1) GN=ambE PE=4 SV=1 | 305.65 |
| P72151\|FLICB_PSEAE | B-type flagellin OS=Pseudomonas aeruginosa (strain ATCC 15692 / DSM 22644 / CIP 104116 / JCM 14847 / LMG 12228 / 1C / PRS 101 / PAO1) GN=fliC PE=1 SV=2 | 302.26 |
| Q9HZ71\|RS1_PSEAE | 30S ribosomal protein S1 OS=Pseudomonas aeruginosa (strain ATCC 15692 / DSM 22644 / CIP 104116 / JCM 14847 / LMG 12228 / 1C / PRS 101 / PAO1) GN=rpsA PE=3 SV=1 | 298.9 |
| tr\|Q9HYR8\|Q9HYR8_PSEAE | Probable non-ribosomal peptide synthetase OS=Pseudomonas aeruginosa (strain ATCC 15692 / DSM 22644 / CIP 104116 / JCM 14847 / LMG 12228 / 1C / PRS 101 / PAO1) GN=PA3327 PE=4 SV=1 | 297.73 |
| Q9LCT3\|SECA_PSEAE | Protein translocase subunit SecA OS=Pseudomonas aeruginosa (strain ATCC 15692 / DSM 22644 / CIP 104116 / JCM 14847 / LMG 12228 / 1C / PRS 101 / PAO1) GN=secA PE=3 SV=1 | 297.25 |
| tr\|Q9HW49\|Q9HW49_PSEAE | Uncharacterized protein OS=Pseudomonas aeruginosa (strain ATCC 15692 / DSM 22644 / CIP 104116 / JCM 14847 / LMG 12228 / 1C / PRS 101 / PAO1) GN=PA4352 PE=3 SV=1 | 293.66 |
| Q9HV43\|DNAK_PSEAE | Chaperone protein DnaK OS=Pseudomonas aeruginosa (strain ATCC 15692 / DSM 22644 / CIP 104116 / JCM 14847 / LMG 12228 / 1C / PRS 101 / PAO1) GN=dnaK PE=3 SV=1 | 293.04 |
| tr\|Q9HVJ1\|Q9HVJ1_PSEAE | Probable ATP-binding component of ABC transporter OS=Pseudomonas aeruginosa (strain ATCC 15692 / DSM 22644 / CIP 104116 / JCM 14847 / LMG 12228 / 1C / PRS 101 / PAO1) GN=PA4595 PE=4 SV=1 | 291.3 |
| Q9HWD2\|EFG1_PSEAE | Elongation factor G 1 OS=Pseudomonas aeruginosa (strain ATCC 15692 / DSM 22644 / CIP 104116 / JCM 14847 / LMG 12228 / 1C / PRS 101 / PAO1) GN=fusA PE=1 SV=1 | 290.84 |
| tr\|Q9I0L5\|Q9I0L5_PSEAE | Isocitrate dehydrogenase [NADP] OS=Pseudomonas aeruginosa (strain ATCC 15692 / DSM 22644 / CIP 104116 / JCM 14847 / LMG 12228 / 1C / PRS 101 / PAO1) GN=icd PE=4 SV=1 | 283.16 |
| tr\|Q9I3D5\|Q9I3D5_PSEAE | Succinate dehydrogenase flavoprotein subunit OS=Pseudomonas aeruginosa (strain ATCC 15692 / DSM 22644 / CIP 104116 / JCM 14847 / LMG 12228 / 1C / PRS 101 / PAO1) GN=sdhA PE=3 SV=1 | 282.64 |
| tr\|Q9I367\|Q9I367_PSEAE | Uncharacterized protein OS=Pseudomonas aeruginosa (strain ATCC 15692 / DSM 22644 / CIP 104116 / JCM 14847 / LMG 12228 / 1C / PRS 101 / PAO1) GN=PA1658 PE=4 SV=1 | 279.65 |
| P47203\|FTSA_PSEAE | Cell division protein FtsA OS=Pseudomonas aeruginosa (strain ATCC 15692 / DSM 22644 / CIP 104116 / JCM 14847 / LMG 12228 / 1C / PRS 101 / PAO1) GN=ftsA PE=3 SV=2 | 278.12 |
| Q9I7C2\|GYRB_PSEAE | DNA gyrase subunit B OS=Pseudomonas aeruginosa (strain ATCC 15692 / DSM 22644 / CIP 104116 / JCM 14847 / LMG 12228 / 1C / PRS 101 / PAO1) GN=gyrB PE=3 SV=1 | 272.23 |
| Q9I0J9\|NUOCD_PSEAE | NADH-quinone oxidoreductase subunit C/D OS=Pseudomonas aeruginosa (strain ATCC 15692 / DSM 22644 / CIP 104116 / JCM 14847 / LMG 12228 / 1C / PRS 101 / PAO1) GN=nuoC PE=3 SV=1 | 272.08 |
| tr\|Q9HVU0\|Q9HVU0_PSEAE | Rod shape-determining protein MreB OS=Pseudomonas aeruginosa (strain ATCC 15692 / DSM 22644 / CIP 104116 / JCM 14847 / LMG 12228 / 1C / PRS 101 / PAO1) GN=mreB PE=4 SV=1 | 266.46 |
| Q9HTV1\|RHO_PSEAE | Transcription termination factor Rho OS=Pseudomonas aeruginosa (strain ATCC 15692 / DSM 22644 / CIP 104116 / JCM 14847 / LMG 12228 / 1C / PRS 101 / PAO1) GN=rho PE=3 SV=1 | 262.41 |
| P08280\|RECA_PSEAE | Protein RecA OS=Pseudomonas aeruginosa (strain ATCC 15692 / DSM 22644 / CIP 104116 / JCM 14847 / LMG 12228 / 1C / PRS 101 / PAO1) GN=recA PE=3 SV=1 | 257.93 |
| Q9HT20\|ATPB_PSEAE | ATP synthase subunit beta OS=Pseudomonas aeruginosa (strain ATCC 15692 / DSM 22644 / CIP 104116 / JCM 14847 / LMG 12228 / 1C / PRS 101 / PAO1) GN=atpD PE=3 SV=1 | 256.9 |
| P13981\|ARCA_PSEAE | Arginine deiminase OS=Pseudomonas aeruginosa (strain ATCC 15692 / DSM 22644 / CIP 104116 / JCM 14847 / LMG 12228 / 1C / PRS 101 / PAO1) GN=arcA PE=1 SV=2 | 250.23 |
| Q9HXZ1\|DPO3A_PSEAE | DNA polymerase III subunit alpha OS=Pseudomonas aeruginosa (strain ATCC 15692 / DSM 22644 / CIP 104116 / JCM 14847 / LMG 12228 / 1C / PRS 101 / PAO1) GN=dnaE PE=3 SV=1 | 247.9 |
| P37798\|ACCC_PSEAE | Biotin carboxylase OS=Pseudomonas aeruginosa (strain ATCC 15692 / DSM 22644 / CIP 104116 / JCM 14847 / LMG 12228 / 1C / PRS 101 / PAO1) GN=accC PE=1 SV=1 | 247.59 |
| G3XD61\|WBPI_PSEAE | UDP-2 3-diacetamido-2 3-dideoxy-D-glucuronate 2-epimerase OS=Pseudomonas aeruginosa (strain ATCC 15692 / DSM 22644 / CIP 104116 / JCM 14847 / LMG 12228 / 1C / PRS 101 / PAO1) GN=wbpI PE=1 SV=1 | 246.78 |
| tr\|Q9I5U9\|Q9I5U9_PSEAE | Uncharacterized protein OS=Pseudomonas aeruginosa (strain ATCC 15692 / DSM 22644 / CIP 104116 / JCM 14847 / LMG 12228 / 1C / PRS 101 / PAO1) GN=PA0588 PE=4 SV=1 | 246.34 |
| P77915\|HEMN_PSEAE | Oxygen-independent coproporphyrinogen III oxidase OS=Pseudomonas aeruginosa (strain ATCC 15692 / DSM 22644 / CIP 104116 / JCM 14847 / LMG 12228 / 1C / PRS 101 / PAO1) GN=hemN PE=2 SV=2 | 246.24 |
| tr\|Q9HYT5\|Q9HYT5_PSEAE | Uncharacterized protein OS=Pseudomonas aeruginosa (strain ATCC 15692 / DSM 22644 / CIP 104116 / JCM 14847 / LMG 12228 / 1C / PRS 101 / PAO1) GN=PA3309 PE=3 SV=1 | 244.35 |
| tr\|Q9HYP7\|Q9HYP7_PSEAE | Probable chemotaxis protein OS=Pseudomonas aeruginosa (strain ATCC 15692 / DSM 22644 / CIP 104116 / JCM 14847 / LMG 12228 / 1C / PRS 101 / PAO1) GN=PA3349 PE=4 SV=1 | 243.5 |
| Q9I2U0\|CLPX_PSEAE | ATP-dependent Clp protease ATP-binding subunit ClpX OS=Pseudomonas aeruginosa (strain ATCC 15692 / DSM 22644 / CIP 104116 / JCM 14847 / LMG 12228 / 1C / PRS 101 / PAO1) GN=clpX PE=3 SV=1 | 243.14 |
| P47204\|FTSZ_PSEAE | Cell division protein FtsZ OS=Pseudomonas aeruginosa (strain ATCC 15692 / DSM 22644 / CIP 104116 / JCM 14847 / LMG 12228 / 1C / PRS 101 / PAO1) GN=ftsZ PE=1 SV=2 | 243.13 |
| O82850\|RS2_PSEAE | 30S ribosomal protein S2 OS=Pseudomonas aeruginosa (strain ATCC 15692 / DSM 22644 / CIP 104116 / JCM 14847 / LMG 12228 / 1C / PRS 101 / PAO1) GN=rpsB PE=3 SV=2 | 239.97 |
| tr\|Q9I524\|Q9I524_PSEAE | GTP pyrophosphokinase OS=Pseudomonas aeruginosa (strain ATCC 15692 / DSM 22644 / CIP 104116 / JCM 14847 / LMG 12228 / 1C / PRS 101 / PAO1) GN=relA PE=3 SV=1 | 239.32 |
| Q9HWE1\|RS3_PSEAE | 30S ribosomal protein S3 OS=Pseudomonas aeruginosa (strain ATCC 15692 / DSM 22644 / CIP 104116 / JCM 14847 / LMG 12228 / 1C / PRS 101 / PAO1) GN=rpsC PE=3 SV=1 | 238.97 |
| tr\|Q9I6Z3\|Q9I6Z3_PSEAE | Alkyl hydroperoxide reductase subunit C OS=Pseudomonas aeruginosa (strain ATCC 15692 / DSM 22644 / CIP 104116 / JCM 14847 / LMG 12228 / 1C / PRS 101 / PAO1) GN=ahpC PE=4 SV=1 | 238.61 |
| Q9HZ76\|WBPE_PSEAE | UDP-2-acetamido-2-deoxy-3-oxo-D-glucuronate aminotransferase OS=Pseudomonas aeruginosa (strain ATCC 15692 / DSM 22644 / CIP 104116 / JCM 14847 / LMG 12228 / 1C / PRS 101 / PAO1) GN=wbpE PE=1 SV=1 | 238.47 |
| tr\|Q9I4I1\|Q9I4I1_PSEAE | Ribonucleoside-diphosphate reductase OS=Pseudomonas aeruginosa (strain ATCC 15692 / DSM 22644 / CIP 104116 / JCM 14847 / LMG 12228 / 1C / PRS 101 / PAO1) GN=nrdA PE=1 SV=1 | 233.73 |
| Q9HV59\|PNP_PSEAE | Polyribonucleotide nucleotidyltransferase OS=Pseudomonas aeruginosa (strain ATCC 15692 / DSM 22644 / CIP 104116 / JCM 14847 / LMG 12228 / 1C / PRS 101 / PAO1) GN=pnp PE=3 SV=1 | 230.28 |
| tr\|Q9I6J3\|Q9I6J3_PSEAE | Probable glutamine synthetase OS=Pseudomonas aeruginosa (strain ATCC 15692 / DSM 22644 / CIP 104116 / JCM 14847 / LMG 12228 / 1C / PRS 101 / PAO1) GN=spuB PE=3 SV=1 | 229.43 |
| Q9I3D2\|ODO2_PSEAE | Dihydrolipoyllysine-residue succinyltransferase component of 2-oxoglutarate dehydrogenase complex OS=Pseudomonas aeruginosa (strain ATCC 15692 / DSM 22644 / CIP 104116 / JCM 14847 / LMG 12228 / 1C / PRS 101 / PAO1) GN=sucB PE=3 SV=1 | 226.08 |
| tr\|Q9I6M1\|Q9I6M1_PSEAE | Alkyl hydroperoxide reductase AhpD OS=Pseudomonas aeruginosa (strain ATCC 15692 / DSM 22644 / CIP 104116 / JCM 14847 / LMG 12228 / 1C / PRS 101 / PAO1) GN=PA0269 PE=1 SV=1 | 220.79 |
| tr\|Q9I0A7\|Q9I0A7_PSEAE | Probable restriction-modification system protein OS=Pseudomonas aeruginosa (strain ATCC 15692 / DSM 22644 / CIP 104116 / JCM 14847 / LMG 12228 / 1C / PRS 101 / PAO1) GN=PA2735 PE=4 SV=1 | 218.98 |
| Q9HWD8\|RL2_PSEAE | 50S ribosomal protein L2 OS=Pseudomonas aeruginosa (strain ATCC 15692 / DSM 22644 / CIP 104116 / JCM 14847 / LMG 12228 / 1C / PRS 101 / PAO1) GN=rplB PE=3 SV=1 | 216.92 |
| Q51561\|RPOB_PSEAE | DNA-directed RNA polymerase subunit beta OS=Pseudomonas aeruginosa (strain ATCC 15692 / DSM 22644 / CIP 104116 / JCM 14847 / LMG 12228 / 1C / PRS 101 / PAO1) GN=rpoB PE=3 SV=2 | 213.05 |
| tr\|Q9I659\|Q9I659_PSEAE | Probable ClpA/B protease ATP binding subunit OS=Pseudomonas aeruginosa (strain ATCC 15692 / DSM 22644 / CIP 104116 / JCM 14847 / LMG 12228 / 1C / PRS 101 / PAO1) GN=PA0459 PE=4 SV=1 | 212.74 |
| Q9HT18\|ATPA_PSEAE | ATP synthase subunit alpha OS=Pseudomonas aeruginosa (strain ATCC 15692 / DSM 22644 / CIP 104116 / JCM 14847 / LMG 12228 / 1C / PRS 101 / PAO1) GN=atpA PE=3 SV=1 | 210.71 |
| tr\|Q9I696\|Q9I696_PSEAE | Component of chemotactic signal transduction system OS=Pseudomonas aeruginosa (strain ATCC 15692 / DSM 22644 / CIP 104116 / JCM 14847 / LMG 12228 / 1C / PRS 101 / PAO1) GN=chpA PE=4 SV=1 | 209.98 |
| tr\|Q9HXR4\|Q9HXR4_PSEAE | Uncharacterized protein OS=Pseudomonas aeruginosa (strain ATCC 15692 / DSM 22644 / CIP 104116 / JCM 14847 / LMG 12228 / 1C / PRS 101 / PAO1) GN=PA3728 PE=4 SV=1 | 206.6 |
| Q9HUL8\|MUTL_PSEAE | DNA mismatch repair protein MutL OS=Pseudomonas aeruginosa (strain ATCC 15692 / DSM 22644 / CIP 104116 / JCM 14847 / LMG 12228 / 1C / PRS 101 / PAO1) GN=mutL PE=3 SV=1 | 206.53 |
| tr\|Q9HUE2\|Q9HUE2_PSEAE | Uncharacterized protein OS=Pseudomonas aeruginosa (strain ATCC 15692 / DSM 22644 / CIP 104116 / JCM 14847 / LMG 12228 / 1C / PRS 101 / PAO1) GN=PA5027 PE=3 SV=1 | 203.78 |
| Q9HV50\|GLMM_PSEAE | Phosphoglucosamine mutase OS=Pseudomonas aeruginosa (strain ATCC 15692 / DSM 22644 / CIP 104116 / JCM 14847 / LMG 12228 / 1C / PRS 101 / PAO1) GN=glmM PE=3 SV=1 | 201.36 |
| Q9HWC6\|RL1_PSEAE | 50S ribosomal protein L1 OS=Pseudomonas aeruginosa (strain ATCC 15692 / DSM 22644 / CIP 104116 / JCM 14847 / LMG 12228 / 1C / PRS 101 / PAO1) GN=rplA PE=3 SV=1 | 200.66 |
| tr\|Q9HVV6\|Q9HVV6_PSEAE | Probable ATP-binding component of ABC transporter OS=Pseudomonas aeruginosa (strain ATCC 15692 / DSM 22644 / CIP 104116 / JCM 14847 / LMG 12228 / 1C / PRS 101 / PAO1) GN=PA4461 PE=1 SV=1 | 198.04 |
| Q9I5Z0\|METK_PSEAE | S-adenosylmethionine synthase OS=Pseudomonas aeruginosa (strain ATCC 15692 / DSM 22644 / CIP 104116 / JCM 14847 / LMG 12228 / 1C / PRS 101 / PAO1) GN=metK PE=3 SV=1 | 197.23 |
| tr\|Q9I0Z1\|Q9I0Z1_PSEAE | Probable oxidoreductase OS=Pseudomonas aeruginosa (strain ATCC 15692 / DSM 22644 / CIP 104116 / JCM 14847 / LMG 12228 / 1C / PRS 101 / PAO1) GN=PA2491 PE=4 SV=1 | 196.15 |
| P13794\|PORF_PSEAE | Outer membrane porin F OS=Pseudomonas aeruginosa (strain ATCC 15692 / DSM 22644 / CIP 104116 / JCM 14847 / LMG 12228 / 1C / PRS 101 / PAO1) GN=oprF PE=1 SV=1 | 195.93 |
| tr\|Q9HY13\|Q9HY13_PSEAE | Uncharacterized protein OS=Pseudomonas aeruginosa (strain ATCC 15692 / DSM 22644 / CIP 104116 / JCM 14847 / LMG 12228 / 1C / PRS 101 / PAO1) GN=PA3613 PE=4 SV=1 | 194.84 |
| tr\|Q9I688\|Q9I688_PSEAE | Uncharacterized protein OS=Pseudomonas aeruginosa (strain ATCC 15692 / DSM 22644 / CIP 104116 / JCM 14847 / LMG 12228 / 1C / PRS 101 / PAO1) GN=PA0429 PE=4 SV=1 | 192.54 |
| Q9I3S3\|GBUA_PSEAE | Guanidinobutyrase OS=Pseudomonas aeruginosa (strain ATCC 15692 / DSM 22644 / CIP 104116 / JCM 14847 / LMG 12228 / 1C / PRS 101 / PAO1) GN=gbuA PE=1 SV=1 | 187.75 |
| Q9I1M2\|ODBA_PSEAE | 2-oxoisovalerate dehydrogenase subunit alpha OS=Pseudomonas aeruginosa (strain ATCC 15692 / DSM 22644 / CIP 104116 / JCM 14847 / LMG 12228 / 1C / PRS 101 / PAO1) GN=bkdA1 PE=3 SV=1 | 187.55 |
| tr\|Q9I363\|Q9I363_PSEAE | Probable ClpA/B-type protease OS=Pseudomonas aeruginosa (strain ATCC 15692 / DSM 22644 / CIP 104116 / JCM 14847 / LMG 12228 / 1C / PRS 101 / PAO1) GN=PA1662 PE=3 SV=1 | 187.32 |
| tr\|G3XD28\|G3XD28_PSEAE | Type 4 fimbrial biogenesis protein PilM OS=Pseudomonas aeruginosa (strain ATCC 15692 / DSM 22644 / CIP 104116 / JCM 14847 / LMG 12228 / 1C / PRS 101 / PAO1) GN=pilM PE=1 SV=1 | 186.86 |
| tr\|Q9HZM8\|Q9HZM8_PSEAE | Ribonuclease E OS=Pseudomonas aeruginosa (strain ATCC 15692 / DSM 22644 / CIP 104116 / JCM 14847 / LMG 12228 / 1C / PRS 101 / PAO1) GN=rne PE=1 SV=1 | 186.55 |
| tr\|G3XCV0\|G3XCV0_PSEAE | Transcriptional regulator FleQ OS=Pseudomonas aeruginosa (strain ATCC 15692 / DSM 22644 / CIP 104116 / JCM 14847 / LMG 12228 / 1C / PRS 101 / PAO1) GN=fleQ PE=1 SV=1 | 185.8 |
| Q9HWC9\|RPOC_PSEAE | DNA-directed RNA polymerase subunit beta' OS=Pseudomonas aeruginosa (strain ATCC 15692 / DSM 22644 / CIP 104116 / JCM 14847 / LMG 12228 / 1C / PRS 101 / PAO1) GN=rpoC PE=3 SV=1 | 184.85 |
| G3XD23\|WBPB_PSEAE | UDP-N-acetyl-2-amino-2-deoxy-D-glucuronate oxidase OS=Pseudomonas aeruginosa (strain ATCC 15692 / DSM 22644 / CIP 104116 / JCM 14847 / LMG 12228 / 1C / PRS 101 / PAO1) GN=wbpB PE=1 SV=1 | 184.6 |
| Q9HWF2\|RS5_PSEAE | 30S ribosomal protein S5 OS=Pseudomonas aeruginosa (strain ATCC 15692 / DSM 22644 / CIP 104116 / JCM 14847 / LMG 12228 / 1C / PRS 101 / PAO1) GN=rpsE PE=3 SV=1 | 184.31 |
| Q9I685\|SAHH_PSEAE | Adenosylhomocysteinase OS=Pseudomonas aeruginosa (strain ATCC 15692 / DSM 22644 / CIP 104116 / JCM 14847 / LMG 12228 / 1C / PRS 101 / PAO1) GN=ahcY PE=3 SV=1 | 183.78 |
| Q9I1M0\|ODB2_PSEAE | Lipoamide acyltransferase component of branched-chain alpha-keto acid dehydrogenase complex OS=Pseudomonas aeruginosa (strain ATCC 15692 / DSM 22644 / CIP 104116 / JCM 14847 / LMG 12228 / 1C / PRS 101 / PAO1) GN=bkdB PE=1 SV=1 | 181.65 |
| Q9HXU0\|SYK_PSEAE | Lysine--tRNA ligase OS=Pseudomonas aeruginosa (strain ATCC 15692 / DSM 22644 / CIP 104116 / JCM 14847 / LMG 12228 / 1C / PRS 101 / PAO1) GN=lysS PE=3 SV=1 | 180.18 |
| Q9HWE7\|RL5_PSEAE | 50S ribosomal protein L5 OS=Pseudomonas aeruginosa (strain ATCC 15692 / DSM 22644 / CIP 104116 / JCM 14847 / LMG 12228 / 1C / PRS 101 / PAO1) GN=rplE PE=3 SV=1 | 178.29 |
| Q9I291\|GALU_PSEAE | UTP--glucose-1-phosphate uridylyltransferase OS=Pseudomonas aeruginosa (strain ATCC 15692 / DSM 22644 / CIP 104116 / JCM 14847 / LMG 12228 / 1C / PRS 101 / PAO1) GN=galU PE=3 SV=1 | 177.83 |
| tr\|Q9I246\|Q9I246_PSEAE | Probable carbamoyl transferase OS=Pseudomonas aeruginosa (strain ATCC 15692 / DSM 22644 / CIP 104116 / JCM 14847 / LMG 12228 / 1C / PRS 101 / PAO1) GN=PA2069 PE=4 SV=1 | 176 |
| tr\|Q9I1F5\|Q9I1F5_PSEAE | Probable glyceraldehyde-3-phosphate dehydrogenase OS=Pseudomonas aeruginosa (strain ATCC 15692 / DSM 22644 / CIP 104116 / JCM 14847 / LMG 12228 / 1C / PRS 101 / PAO1) GN=PA2323 PE=4 SV=1 | 175.55 |
| tr\|Q9I3B2\|Q9I3B2_PSEAE | Beta-ketoacyl-ACP synthase I OS=Pseudomonas aeruginosa (strain ATCC 15692 / DSM 22644 / CIP 104116 / JCM 14847 / LMG 12228 / 1C / PRS 101 / PAO1) GN=fabB PE=3 SV=1 | 173.53 |
| Q9I6Z1\|PK21A_PSEAE | Polyphosphate:ADP/GDP phosphotransferase OS=Pseudomonas aeruginosa (strain ATCC 15692 / DSM 22644 / CIP 104116 / JCM 14847 / LMG 12228 / 1C / PRS 101 / PAO1) GN=ppk2 PE=1 SV=2 | 173.22 |
| Q9HUM9\|RS6_PSEAE | 30S ribosomal protein S6 OS=Pseudomonas aeruginosa (strain ATCC 15692 / DSM 22644 / CIP 104116 / JCM 14847 / LMG 12228 / 1C / PRS 101 / PAO1) GN=rpsF PE=3 SV=1 | 172.53 |
| tr\|G3XD74\|G3XD74_PSEAE | D-ala-D-ala-carboxypeptidase OS=Pseudomonas aeruginosa (strain ATCC 15692 / DSM 22644 / CIP 104116 / JCM 14847 / LMG 12228 / 1C / PRS 101 / PAO1) GN=dacC PE=3 SV=1 | 172.37 |
| Q9HVW7\|MURA_PSEAE | UDP-N-acetylglucosamine 1-carboxyvinyltransferase OS=Pseudomonas aeruginosa (strain ATCC 15692 / DSM 22644 / CIP 104116 / JCM 14847 / LMG 12228 / 1C / PRS 101 / PAO1) GN=murA PE=1 SV=1 | 171.02 |
| Q9I3D1\|DLDH2_PSEAE | Dihydrolipoyl dehydrogenase OS=Pseudomonas aeruginosa (strain ATCC 15692 / DSM 22644 / CIP 104116 / JCM 14847 / LMG 12228 / 1C / PRS 101 / PAO1) GN=lpdG PE=1 SV=1 | 170.51 |
| P54292\|RHLR_PSEAE | Regulatory protein RhlR OS=Pseudomonas aeruginosa (strain ATCC 15692 / DSM 22644 / CIP 104116 / JCM 14847 / LMG 12228 / 1C / PRS 101 / PAO1) GN=rhlR PE=3 SV=1 | 170.45 |
| Q51422\|SYDND_PSEAE | Aspartate--tRNA(Asp/Asn) ligase OS=Pseudomonas aeruginosa (strain ATCC 15692 / DSM 22644 / CIP 104116 / JCM 14847 / LMG 12228 / 1C / PRS 101 / PAO1) GN=aspS PE=1 SV=2 | 170.44 |
| Q9HZ66\|SERC_PSEAE | Phosphoserine aminotransferase OS=Pseudomonas aeruginosa (strain ATCC 15692 / DSM 22644 / CIP 104116 / JCM 14847 / LMG 12228 / 1C / PRS 101 / PAO1) GN=serC PE=1 SV=1 | 169.81 |
| tr\|Q9I5V1\|Q9I5V1_PSEAE | Uncharacterized protein OS=Pseudomonas aeruginosa (strain ATCC 15692 / DSM 22644 / CIP 104116 / JCM 14847 / LMG 12228 / 1C / PRS 101 / PAO1) GN=PA0586 PE=4 SV=1 | 169.58 |
| tr\|Q9I3D3\|Q9I3D3_PSEAE | 2-oxoglutarate dehydrogenase (E1 subunit) OS=Pseudomonas aeruginosa (strain ATCC 15692 / DSM 22644 / CIP 104116 / JCM 14847 / LMG 12228 / 1C / PRS 101 / PAO1) GN=sucA PE=4 SV=1 | 168.7 |
| Q9HWD4\|RS10_PSEAE | 30S ribosomal protein S10 OS=Pseudomonas aeruginosa (strain ATCC 15692 / DSM 22644 / CIP 104116 / JCM 14847 / LMG 12228 / 1C / PRS 101 / PAO1) GN=rpsJ PE=3 SV=1 | 167.31 |
| P04739\|FMPO_PSEAE | Fimbrial protein OS=Pseudomonas aeruginosa (strain ATCC 15692 / DSM 22644 / CIP 104116 / JCM 14847 / LMG 12228 / 1C / PRS 101 / PAO1) GN=pilA PE=1 SV=1 | 165.81 |
| tr\|Q9HUG0\|Q9HUG0_PSEAE | Probable carbamoyl transferase OS=Pseudomonas aeruginosa (strain ATCC 15692 / DSM 22644 / CIP 104116 / JCM 14847 / LMG 12228 / 1C / PRS 101 / PAO1) GN=PA5005 PE=4 SV=1 | 165.18 |
| tr\|Q9I3D4\|Q9I3D4_PSEAE | Succinate dehydrogenase (B subunit) OS=Pseudomonas aeruginosa (strain ATCC 15692 / DSM 22644 / CIP 104116 / JCM 14847 / LMG 12228 / 1C / PRS 101 / PAO1) GN=sdhB PE=4 SV=1 | 164.9 |
| P08308\|OTCC_PSEAE | Ornithine carbamoyltransferase catabolic OS=Pseudomonas aeruginosa (strain ATCC 15692 / DSM 22644 / CIP 104116 / JCM 14847 / LMG 12228 / 1C / PRS 101 / PAO1) GN=arcB PE=1 SV=3 | 162.41 |
| P38100\|CARB_PSEAE | Carbamoyl-phosphate synthase large chain OS=Pseudomonas aeruginosa (strain ATCC 15692 / DSM 22644 / CIP 104116 / JCM 14847 / LMG 12228 / 1C / PRS 101 / PAO1) GN=carB PE=3 SV=3 | 160.96 |
| P72138\|HIS52_PSEAE | Imidazole glycerol phosphate synthase subunit HisH 2 OS=Pseudomonas aeruginosa (strain ATCC 15692 / DSM 22644 / CIP 104116 / JCM 14847 / LMG 12228 / 1C / PRS 101 / PAO1) GN=hisH2 PE=3 SV=2 | 160.81 |
| tr\|Q9HTU9\|Q9HTU9_PSEAE | Glyoxalase OS=Pseudomonas aeruginosa (strain ATCC 15692 / DSM 22644 / CIP 104116 / JCM 14847 / LMG 12228 / 1C / PRS 101 / PAO1) GN=PA5245 PE=3 SV=1 | 160.54 |
| tr\|Q9HZK4\|Q9HZK4_PSEAE | Glyceraldehyde-3-phosphate dehydrogenase OS=Pseudomonas aeruginosa (strain ATCC 15692 / DSM 22644 / CIP 104116 / JCM 14847 / LMG 12228 / 1C / PRS 101 / PAO1) GN=PA3001 PE=3 SV=1 | 159.49 |
| P29365\|DHOM_PSEAE | Homoserine dehydrogenase OS=Pseudomonas aeruginosa (strain ATCC 15692 / DSM 22644 / CIP 104116 / JCM 14847 / LMG 12228 / 1C / PRS 101 / PAO1) GN=hom PE=3 SV=2 | 158.93 |
| tr\|Q9HXR8\|Q9HXR8_PSEAE | Probable FMN oxidoreductase OS=Pseudomonas aeruginosa (strain ATCC 15692 / DSM 22644 / CIP 104116 / JCM 14847 / LMG 12228 / 1C / PRS 101 / PAO1) GN=PA3723 PE=4 SV=1 | 158.25 |
| tr\|Q9HTD1\|Q9HTD1_PSEAE | Probable transcarboxylase subunit OS=Pseudomonas aeruginosa (strain ATCC 15692 / DSM 22644 / CIP 104116 / JCM 14847 / LMG 12228 / 1C / PRS 101 / PAO1) GN=PA5435 PE=4 SV=1 | 157.19 |
| Q9I383\|SELD_PSEAE | Selenide water dikinase OS=Pseudomonas aeruginosa (strain ATCC 15692 / DSM 22644 / CIP 104116 / JCM 14847 / LMG 12228 / 1C / PRS 101 / PAO1) GN=selD PE=3 SV=1 | 157.04 |
| Q9HU19\|DCTD_PSEAE | C4-dicarboxylate transport transcriptional regulatory protein DctD OS=Pseudomonas aeruginosa (strain ATCC 15692 / DSM 22644 / CIP 104116 / JCM 14847 / LMG 12228 / 1C / PRS 101 / PAO1) GN=dctD PE=3 SV=1 | 156.49 |
| tr\|G3XD36\|G3XD36_PSEAE | Phosphoenolpyruvate-protein phosphotransferase PtsP OS=Pseudomonas aeruginosa (strain ATCC 15692 / DSM 22644 / CIP 104116 / JCM 14847 / LMG 12228 / 1C / PRS 101 / PAO1) GN=ptsP PE=3 SV=1 | 156.31 |
| Q9HV44\|DNAJ_PSEAE | Chaperone protein DnaJ OS=Pseudomonas aeruginosa (strain ATCC 15692 / DSM 22644 / CIP 104116 / JCM 14847 / LMG 12228 / 1C / PRS 101 / PAO1) GN=dnaJ PE=3 SV=1 | 155.24 |
| tr\|Q9I1Z6\|Q9I1Z6_PSEAE | Alcohol dehydrogenase (Zn-dependent) OS=Pseudomonas aeruginosa (strain ATCC 15692 / DSM 22644 / CIP 104116 / JCM 14847 / LMG 12228 / 1C / PRS 101 / PAO1) GN=PA2119 PE=3 SV=1 | 154.92 |
| tr\|Q9HTM0\|Q9HTM0_PSEAE | Guanosine-3' 5'-bis(Diphosphate) 3'-pyrophosphohydrolase OS=Pseudomonas aeruginosa (strain ATCC 15692 / DSM 22644 / CIP 104116 / JCM 14847 / LMG 12228 / 1C / PRS 101 / PAO1) GN=spoT PE=3 SV=1 | 154.84 |
| tr\|Q9HWQ0\|Q9HWQ0_PSEAE | Probable iron-sulfur protein OS=Pseudomonas aeruginosa (strain ATCC 15692 / DSM 22644 / CIP 104116 / JCM 14847 / LMG 12228 / 1C / PRS 101 / PAO1) GN=PA4131 PE=4 SV=1 | 152.86 |
| G3XD12\|HCNC_PSEAE | Hydrogen cyanide synthase subunit HcnC OS=Pseudomonas aeruginosa (strain ATCC 15692 / DSM 22644 / CIP 104116 / JCM 14847 / LMG 12228 / 1C / PRS 101 / PAO1) GN=hcnC PE=1 SV=1 | 152.07 |
| tr\|Q9I046\|Q9I046_PSEAE | Uncharacterized protein OS=Pseudomonas aeruginosa (strain ATCC 15692 / DSM 22644 / CIP 104116 / JCM 14847 / LMG 12228 / 1C / PRS 101 / PAO1) GN=PA2797 PE=4 SV=1 | 151.68 |
| Q9HVC4\|RL25_PSEAE | 50S ribosomal protein L25 OS=Pseudomonas aeruginosa (strain ATCC 15692 / DSM 22644 / CIP 104116 / JCM 14847 / LMG 12228 / 1C / PRS 101 / PAO1) GN=rplY PE=3 SV=1 | 151.68 |
| tr\|Q9HX94\|Q9HX94_PSEAE | Uncharacterized protein OS=Pseudomonas aeruginosa (strain ATCC 15692 / DSM 22644 / CIP 104116 / JCM 14847 / LMG 12228 / 1C / PRS 101 / PAO1) GN=PA3919 PE=4 SV=1 | 151.57 |
| P42807\|HEM1_PSEAE | Glutamyl-tRNA reductase OS=Pseudomonas aeruginosa (strain ATCC 15692 / DSM 22644 / CIP 104116 / JCM 14847 / LMG 12228 / 1C / PRS 101 / PAO1) GN=hemA PE=3 SV=3 | 151.51 |
| P55222\|VFR_PSEAE | Cyclic AMP receptor-like protein OS=Pseudomonas aeruginosa (strain ATCC 15692 / DSM 22644 / CIP 104116 / JCM 14847 / LMG 12228 / 1C / PRS 101 / PAO1) GN=vfr PE=1 SV=1 | 150.21 |
| Q9HV42\|GRPE_PSEAE | Protein GrpE OS=Pseudomonas aeruginosa (strain ATCC 15692 / DSM 22644 / CIP 104116 / JCM 14847 / LMG 12228 / 1C / PRS 101 / PAO1) GN=grpE PE=3 SV=1 | 150.15 |
| tr\|Q9I2Z5\|Q9I2Z5_PSEAE | Probable amidotransferase OS=Pseudomonas aeruginosa (strain ATCC 15692 / DSM 22644 / CIP 104116 / JCM 14847 / LMG 12228 / 1C / PRS 101 / PAO1) GN=PA1742 PE=4 SV=1 | 149.99 |
| Q9HY08\|MUTS_PSEAE | DNA mismatch repair protein MutS OS=Pseudomonas aeruginosa (strain ATCC 15692 / DSM 22644 / CIP 104116 / JCM 14847 / LMG 12228 / 1C / PRS 101 / PAO1) GN=mutS PE=3 SV=1 | 149.94 |
| Q51373\|GACA_PSEAE | Response regulator GacA OS=Pseudomonas aeruginosa (strain ATCC 15692 / DSM 22644 / CIP 104116 / JCM 14847 / LMG 12228 / 1C / PRS 101 / PAO1) GN=gacA PE=3 SV=2 | 148.01 |
| tr\|Q9HY81\|Q9HY81_PSEAE | Probable peroxidase OS=Pseudomonas aeruginosa (strain ATCC 15692 / DSM 22644 / CIP 104116 / JCM 14847 / LMG 12228 / 1C / PRS 101 / PAO1) GN=PA3529 PE=1 SV=1 | 147.06 |
| tr\|Q9HXI9\|Q9HXI9_PSEAE | Iron-sulfur cluster assembly scaffold protein IscU OS=Pseudomonas aeruginosa (strain ATCC 15692 / DSM 22644 / CIP 104116 / JCM 14847 / LMG 12228 / 1C / PRS 101 / PAO1) GN=iscU PE=3 SV=1 | 145.41 |
| O52759\|RS4_PSEAE | 30S ribosomal protein S4 OS=Pseudomonas aeruginosa (strain ATCC 15692 / DSM 22644 / CIP 104116 / JCM 14847 / LMG 12228 / 1C / PRS 101 / PAO1) GN=rpsD PE=3 SV=2 | 145.07 |
| Q9HYX7\|RDGC_PSEAE | Recombination-associated protein RdgC OS=Pseudomonas aeruginosa (strain ATCC 15692 / DSM 22644 / CIP 104116 / JCM 14847 / LMG 12228 / 1C / PRS 101 / PAO1) GN=rdgC PE=1 SV=1 | 144.52 |
| O52762\|CATA_PSEAE | Catalase OS=Pseudomonas aeruginosa (strain ATCC 15692 / DSM 22644 / CIP 104116 / JCM 14847 / LMG 12228 / 1C / PRS 101 / PAO1) GN=katA PE=1 SV=1 | 143.9 |
| tr\|G3XD04\|G3XD04_PSEAE | DNA helicase OS=Pseudomonas aeruginosa (strain ATCC 15692 / DSM 22644 / CIP 104116 / JCM 14847 / LMG 12228 / 1C / PRS 101 / PAO1) GN=uvrD PE=3 SV=1 | 143.74 |
| Q9HWF1\|RL18_PSEAE | 50S ribosomal protein L18 OS=Pseudomonas aeruginosa (strain ATCC 15692 / DSM 22644 / CIP 104116 / JCM 14847 / LMG 12228 / 1C / PRS 101 / PAO1) GN=rplR PE=3 SV=1 | 143.69 |
| Q9HWX4\|RIBB_PSEAE | 3 4-dihydroxy-2-butanone 4-phosphate synthase OS=Pseudomonas aeruginosa (strain ATCC 15692 / DSM 22644 / CIP 104116 / JCM 14847 / LMG 12228 / 1C / PRS 101 / PAO1) GN=ribB PE=3 SV=1 | 143.02 |
| tr\|G3XCX3\|G3XCX3_PSEAE | Twitching motility protein PilU OS=Pseudomonas aeruginosa (strain ATCC 15692 / DSM 22644 / CIP 104116 / JCM 14847 / LMG 12228 / 1C / PRS 101 / PAO1) GN=pilU PE=4 SV=1 | 142.71 |
| Q9HWE5\|RL14_PSEAE | 50S ribosomal protein L14 OS=Pseudomonas aeruginosa (strain ATCC 15692 / DSM 22644 / CIP 104116 / JCM 14847 / LMG 12228 / 1C / PRS 101 / PAO1) GN=rplN PE=3 SV=1 | 140.97 |
| Q9HWE2\|RL16_PSEAE | 50S ribosomal protein L16 OS=Pseudomonas aeruginosa (strain ATCC 15692 / DSM 22644 / CIP 104116 / JCM 14847 / LMG 12228 / 1C / PRS 101 / PAO1) GN=rplP PE=3 SV=1 | 140.55 |
| tr\|Q9I4I2\|Q9I4I2_PSEAE | Ribonucleoside-diphosphate reductase subunit beta OS=Pseudomonas aeruginosa (strain ATCC 15692 / DSM 22644 / CIP 104116 / JCM 14847 / LMG 12228 / 1C / PRS 101 / PAO1) GN=nrdB PE=3 SV=1 | 140.28 |
| tr\|Q9HZ98\|Q9HZ98_PSEAE | Heat-shock protein IbpA OS=Pseudomonas aeruginosa (strain ATCC 15692 / DSM 22644 / CIP 104116 / JCM 14847 / LMG 12228 / 1C / PRS 101 / PAO1) GN=ibpA PE=3 SV=1 | 140.2 |
| Q9I0K0\|NUOB_PSEAE | NADH-quinone oxidoreductase subunit B OS=Pseudomonas aeruginosa (strain ATCC 15692 / DSM 22644 / CIP 104116 / JCM 14847 / LMG 12228 / 1C / PRS 101 / PAO1) GN=nuoB PE=3 SV=1 | 139.64 |
| Q59638\|ODP2_PSEAE | Dihydrolipoyllysine-residue acetyltransferase component of pyruvate dehydrogenase complex OS=Pseudomonas aeruginosa (strain ATCC 15692 / DSM 22644 / CIP 104116 / JCM 14847 / LMG 12228 / 1C / PRS 101 / PAO1) GN=aceF PE=2 SV=2 | 139.1 |
| O54439\|ACP1_PSEAE | Acyl carrier protein 1 OS=Pseudomonas aeruginosa (strain ATCC 15692 / DSM 22644 / CIP 104116 / JCM 14847 / LMG 12228 / 1C / PRS 101 / PAO1) GN=acpP1 PE=3 SV=4 | 138.97 |
| Q9HWC7\|RL10_PSEAE | 50S ribosomal protein L10 OS=Pseudomonas aeruginosa (strain ATCC 15692 / DSM 22644 / CIP 104116 / JCM 14847 / LMG 12228 / 1C / PRS 101 / PAO1) GN=rplJ PE=3 SV=1 | 138.68 |
| Q51470\|MIAB_PSEAE | tRNA-2-methylthio-N(6)-dimethylallyladenosine synthase OS=Pseudomonas aeruginosa (strain ATCC 15692 / DSM 22644 / CIP 104116 / JCM 14847 / LMG 12228 / 1C / PRS 101 / PAO1) GN=miaB PE=3 SV=2 | 138.45 |
| P05384\|DBHB_PSEAE | DNA-binding protein HU-beta OS=Pseudomonas aeruginosa (strain ATCC 15692 / DSM 22644 / CIP 104116 / JCM 14847 / LMG 12228 / 1C / PRS 101 / PAO1) GN=hupB PE=1 SV=3 | 137.66 |
| P49988\|RP54_PSEAE | RNA polymerase sigma-54 factor OS=Pseudomonas aeruginosa (strain ATCC 15692 / DSM 22644 / CIP 104116 / JCM 14847 / LMG 12228 / 1C / PRS 101 / PAO1) GN=rpoN PE=3 SV=2 | 137.56 |
| Q9HVL9\|PROB_PSEAE | Glutamate 5-kinase OS=Pseudomonas aeruginosa (strain ATCC 15692 / DSM 22644 / CIP 104116 / JCM 14847 / LMG 12228 / 1C / PRS 101 / PAO1) GN=proB PE=3 SV=1 | 137.15 |
| tr\|Q9I671\|Q9I671_PSEAE | Glutaryl-CoA dehydrogenase OS=Pseudomonas aeruginosa (strain ATCC 15692 / DSM 22644 / CIP 104116 / JCM 14847 / LMG 12228 / 1C / PRS 101 / PAO1) GN=gcdH PE=3 SV=1 | 137.04 |
| tr\|Q9I3G2\|Q9I3G2_PSEAE | Cbb3-type cytochrome c oxidase subunit OS=Pseudomonas aeruginosa (strain ATCC 15692 / DSM 22644 / CIP 104116 / JCM 14847 / LMG 12228 / 1C / PRS 101 / PAO1) GN=ccoP2 PE=3 SV=1 | 136.87 |
| tr\|Q9I368\|Q9I368_PSEAE | Uncharacterized protein OS=Pseudomonas aeruginosa (strain ATCC 15692 / DSM 22644 / CIP 104116 / JCM 14847 / LMG 12228 / 1C / PRS 101 / PAO1) GN=PA1657 PE=4 SV=1 | 136.63 |
| tr\|Q9HYC8\|Q9HYC8_PSEAE | Iron-sulfur cluster carrier protein OS=Pseudomonas aeruginosa (strain ATCC 15692 / DSM 22644 / CIP 104116 / JCM 14847 / LMG 12228 / 1C / PRS 101 / PAO1) GN=PA3481 PE=3 SV=1 | 136.31 |
| Q9X2T1\|THIO_PSEAE | Thioredoxin OS=Pseudomonas aeruginosa (strain ATCC 15692 / DSM 22644 / CIP 104116 / JCM 14847 / LMG 12228 / 1C / PRS 101 / PAO1) GN=trxA PE=3 SV=1 | 136.29 |
| tr\|Q9I1N4\|Q9I1N4_PSEAE | PslE OS=Pseudomonas aeruginosa (strain ATCC 15692 / DSM 22644 / CIP 104116 / JCM 14847 / LMG 12228 / 1C / PRS 101 / PAO1) GN=pslE PE=4 SV=1 | 136.23 |
| Q9HWH2\|PHZM_PSEAE | Phenazine-1-carboxylate N-methyltransferase OS=Pseudomonas aeruginosa (strain ATCC 15692 / DSM 22644 / CIP 104116 / JCM 14847 / LMG 12228 / 1C / PRS 101 / PAO1) GN=phzM PE=1 SV=1 | 136.2 |
| Q9HYR9\|CLPP2_PSEAE | ATP-dependent Clp protease proteolytic subunit 2 OS=Pseudomonas aeruginosa (strain ATCC 15692 / DSM 22644 / CIP 104116 / JCM 14847 / LMG 12228 / 1C / PRS 101 / PAO1) GN=clpP2 PE=3 SV=1 | 135.73 |
| tr\|Q9I5F6\|Q9I5F6_PSEAE | Bifunctional protein PutA OS=Pseudomonas aeruginosa (strain ATCC 15692 / DSM 22644 / CIP 104116 / JCM 14847 / LMG 12228 / 1C / PRS 101 / PAO1) GN=putA PE=3 SV=1 | 135.26 |
| Q9HUC0\|UBIE_PSEAE | Ubiquinone/menaquinone biosynthesis C-methyltransferase UbiE OS=Pseudomonas aeruginosa (strain ATCC 15692 / DSM 22644 / CIP 104116 / JCM 14847 / LMG 12228 / 1C / PRS 101 / PAO1) GN=ubiE PE=3 SV=1 | 135 |
| Q9HXZ5\|ENO_PSEAE | Enolase OS=Pseudomonas aeruginosa (strain ATCC 15692 / DSM 22644 / CIP 104116 / JCM 14847 / LMG 12228 / 1C / PRS 101 / PAO1) GN=eno PE=3 SV=1 | 134.81 |
| Q9HY63\|ARNA_PSEAE | Bifunctional polymyxin resistance protein ArnA OS=Pseudomonas aeruginosa (strain ATCC 15692 / DSM 22644 / CIP 104116 / JCM 14847 / LMG 12228 / 1C / PRS 101 / PAO1) GN=arnA PE=3 SV=1 | 134.65 |
| Q9HWD5\|RL3_PSEAE | 50S ribosomal protein L3 OS=Pseudomonas aeruginosa (strain ATCC 15692 / DSM 22644 / CIP 104116 / JCM 14847 / LMG 12228 / 1C / PRS 101 / PAO1) GN=rplC PE=3 SV=1 | 134.32 |
| P21629\|BRAF_PSEAE | High-affinity branched-chain amino acid transport ATP-binding protein BraF OS=Pseudomonas aeruginosa (strain ATCC 15692 / DSM 22644 / CIP 104116 / JCM 14847 / LMG 12228 / 1C / PRS 101 / PAO1) GN=braF PE=3 SV=1 | 133.8 |
| tr\|Q9I3C4\|Q9I3C4_PSEAE | Uncharacterized protein OS=Pseudomonas aeruginosa (strain ATCC 15692 / DSM 22644 / CIP 104116 / JCM 14847 / LMG 12228 / 1C / PRS 101 / PAO1) GN=PA1597 PE=4 SV=1 | 133.69 |
| tr\|Q9HWV0\|Q9HWV0_PSEAE | Probable nonribosomal peptide synthetase OS=Pseudomonas aeruginosa (strain ATCC 15692 / DSM 22644 / CIP 104116 / JCM 14847 / LMG 12228 / 1C / PRS 101 / PAO1) GN=PA4078 PE=4 SV=1 | 132.96 |
| Q51567\|SUCD_PSEAE | Succinate--CoA ligase [ADP-forming] subunit alpha OS=Pseudomonas aeruginosa (strain ATCC 15692 / DSM 22644 / CIP 104116 / JCM 14847 / LMG 12228 / 1C / PRS 101 / PAO1) GN=sucD PE=1 SV=2 | 132.34 |
| tr\|Q9I0R4\|Q9I0R4_PSEAE | Probable two-component response regulator OS=Pseudomonas aeruginosa (strain ATCC 15692 / DSM 22644 / CIP 104116 / JCM 14847 / LMG 12228 / 1C / PRS 101 / PAO1) GN=PA2572 PE=4 SV=1 | 132.26 |
| P0DP44\|PPK1_PSEAE | Polyphosphate kinase OS=Pseudomonas aeruginosa (strain ATCC 15692 / DSM 22644 / CIP 104116 / JCM 14847 / LMG 12228 / 1C / PRS 101 / PAO1) GN=ppk PE=1 SV=1 | 131.84 |
| tr\|Q9I0B5\|Q9I0B5_PSEAE | Uncharacterized protein OS=Pseudomonas aeruginosa (strain ATCC 15692 / DSM 22644 / CIP 104116 / JCM 14847 / LMG 12228 / 1C / PRS 101 / PAO1) GN=PA2727 PE=4 SV=1 | 131.25 |
| Q9I5G8\|LEPA_PSEAE | Elongation factor 4 OS=Pseudomonas aeruginosa (strain ATCC 15692 / DSM 22644 / CIP 104116 / JCM 14847 / LMG 12228 / 1C / PRS 101 / PAO1) GN=lepA PE=3 SV=1 | 131.16 |
| Q9LCT6\|DDLB_PSEAE | D-alanine--D-alanine ligase B OS=Pseudomonas aeruginosa (strain ATCC 15692 / DSM 22644 / CIP 104116 / JCM 14847 / LMG 12228 / 1C / PRS 101 / PAO1) GN=ddlB PE=3 SV=1 | 131.15 |
| Q9I5V0\|Y587_PSEAE | UPF0229 protein PA0587 OS=Pseudomonas aeruginosa (strain ATCC 15692 / DSM 22644 / CIP 104116 / JCM 14847 / LMG 12228 / 1C / PRS 101 / PAO1) GN=PA0587 PE=3 SV=1 | 130.84 |
| Q9I3F5\|ACNA_PSEAE | Aconitate hydratase A OS=Pseudomonas aeruginosa (strain ATCC 15692 / DSM 22644 / CIP 104116 / JCM 14847 / LMG 12228 / 1C / PRS 101 / PAO1) GN=acnA PE=3 SV=1 | 130.28 |
| Q9HUE6\|Y5023_PSEAE | UPF0061 protein PA5023 OS=Pseudomonas aeruginosa (strain ATCC 15692 / DSM 22644 / CIP 104116 / JCM 14847 / LMG 12228 / 1C / PRS 101 / PAO1) GN=PA5023 PE=3 SV=1 | 130.14 |
| Q9I7C5\|DNAA_PSEAE | Chromosomal replication initiator protein DnaA OS=Pseudomonas aeruginosa (strain ATCC 15692 / DSM 22644 / CIP 104116 / JCM 14847 / LMG 12228 / 1C / PRS 101 / PAO1) GN=dnaA PE=3 SV=1 | 129.32 |
| tr\|Q9I3I1\|Q9I3I1_PSEAE | DNA polymerase III subunit gamma/tau OS=Pseudomonas aeruginosa (strain ATCC 15692 / DSM 22644 / CIP 104116 / JCM 14847 / LMG 12228 / 1C / PRS 101 / PAO1) GN=dnaX PE=3 SV=1 | 129.21 |
| Q51390\|GLPK2_PSEAE | Glycerol kinase 2 OS=Pseudomonas aeruginosa (strain ATCC 15692 / DSM 22644 / CIP 104116 / JCM 14847 / LMG 12228 / 1C / PRS 101 / PAO1) GN=glpK2 PE=3 SV=2 | 128.38 |
| tr\|Q9HVA0\|Q9HVA0_PSEAE | Acetolactate synthase OS=Pseudomonas aeruginosa (strain ATCC 15692 / DSM 22644 / CIP 104116 / JCM 14847 / LMG 12228 / 1C / PRS 101 / PAO1) GN=ilvI PE=3 SV=1 | 126.86 |
| Q9HWC5\|RL11_PSEAE | 50S ribosomal protein L11 OS=Pseudomonas aeruginosa (strain ATCC 15692 / DSM 22644 / CIP 104116 / JCM 14847 / LMG 12228 / 1C / PRS 101 / PAO1) GN=rplK PE=3 SV=1 | 125.7 |
| Q9HWD6\|RL4_PSEAE | 50S ribosomal protein L4 OS=Pseudomonas aeruginosa (strain ATCC 15692 / DSM 22644 / CIP 104116 / JCM 14847 / LMG 12228 / 1C / PRS 101 / PAO1) GN=rplD PE=3 SV=1 | 125.11 |
| tr\|Q9HUW8\|Q9HUW8_PSEAE | Uncharacterized protein OS=Pseudomonas aeruginosa (strain ATCC 15692 / DSM 22644 / CIP 104116 / JCM 14847 / LMG 12228 / 1C / PRS 101 / PAO1) GN=PA4842 PE=4 SV=1 | 124.82 |
| tr\|Q9I2W9\|Q9I2W9_PSEAE | Phosphoenolpyruvate synthase OS=Pseudomonas aeruginosa (strain ATCC 15692 / DSM 22644 / CIP 104116 / JCM 14847 / LMG 12228 / 1C / PRS 101 / PAO1) GN=ppsA PE=3 SV=1 | 124.17 |
| P96963\|RADA_PSEAE | DNA repair protein RadA OS=Pseudomonas aeruginosa (strain ATCC 15692 / DSM 22644 / CIP 104116 / JCM 14847 / LMG 12228 / 1C / PRS 101 / PAO1) GN=radA PE=3 SV=2 | 124.11 |
| P47205\|LPXC_PSEAE | UDP-3-O-acyl-N-acetylglucosamine deacetylase OS=Pseudomonas aeruginosa (strain ATCC 15692 / DSM 22644 / CIP 104116 / JCM 14847 / LMG 12228 / 1C / PRS 101 / PAO1) GN=lpxC PE=1 SV=2 | 123.81 |
| Q9I1S2\|HCNB_PSEAE | Hydrogen cyanide synthase subunit HcnB OS=Pseudomonas aeruginosa (strain ATCC 15692 / DSM 22644 / CIP 104116 / JCM 14847 / LMG 12228 / 1C / PRS 101 / PAO1) GN=hcnB PE=1 SV=1 | 123.4 |
| tr\|Q9HZI7\|Q9HZI7_PSEAE | Probable ATP-binding component of ABC transporter OS=Pseudomonas aeruginosa (strain ATCC 15692 / DSM 22644 / CIP 104116 / JCM 14847 / LMG 12228 / 1C / PRS 101 / PAO1) GN=PA3019 PE=4 SV=1 | 122.96 |
| tr\|Q9HY79\|Q9HY79_PSEAE | Bacterioferritin OS=Pseudomonas aeruginosa (strain ATCC 15692 / DSM 22644 / CIP 104116 / JCM 14847 / LMG 12228 / 1C / PRS 101 / PAO1) GN=bfrB PE=1 SV=1 | 122.85 |
| Q9HWF8\|RS11_PSEAE | 30S ribosomal protein S11 OS=Pseudomonas aeruginosa (strain ATCC 15692 / DSM 22644 / CIP 104116 / JCM 14847 / LMG 12228 / 1C / PRS 101 / PAO1) GN=rpsK PE=3 SV=1 | 122.43 |
| P53641\|SODF_PSEAE | Superoxide dismutase [Fe] OS=Pseudomonas aeruginosa (strain ATCC 15692 / DSM 22644 / CIP 104116 / JCM 14847 / LMG 12228 / 1C / PRS 101 / PAO1) GN=sodB PE=3 SV=3 | 122.19 |
| Q9HVL6\|RL21_PSEAE | 50S ribosomal protein L21 OS=Pseudomonas aeruginosa (strain ATCC 15692 / DSM 22644 / CIP 104116 / JCM 14847 / LMG 12228 / 1C / PRS 101 / PAO1) GN=rplU PE=3 SV=1 | 121.95 |
| tr\|Q9I2Z1\|Q9I2Z1_PSEAE | Uncharacterized protein OS=Pseudomonas aeruginosa (strain ATCC 15692 / DSM 22644 / CIP 104116 / JCM 14847 / LMG 12228 / 1C / PRS 101 / PAO1) GN=PA1746 PE=4 SV=1 | 121.43 |
| Q9HUG9\|HLDE_PSEAE | Bifunctional protein HldE OS=Pseudomonas aeruginosa (strain ATCC 15692 / DSM 22644 / CIP 104116 / JCM 14847 / LMG 12228 / 1C / PRS 101 / PAO1) GN=hldE PE=3 SV=1 | 119.96 |
| Q9HXQ2\|RL19_PSEAE | 50S ribosomal protein L19 OS=Pseudomonas aeruginosa (strain ATCC 15692 / DSM 22644 / CIP 104116 / JCM 14847 / LMG 12228 / 1C / PRS 101 / PAO1) GN=rplS PE=3 SV=1 | 119.95 |
| Q9HVW0\|KDSD_PSEAE | Arabinose 5-phosphate isomerase KdsD OS=Pseudomonas aeruginosa (strain ATCC 15692 / DSM 22644 / CIP 104116 / JCM 14847 / LMG 12228 / 1C / PRS 101 / PAO1) GN=kdsD PE=1 SV=1 | 119.31 |
| G3XD01\|WBPD_PSEAE | UDP-2-acetamido-3-amino-2 3-dideoxy-D-glucuronate N-acetyltransferase OS=Pseudomonas aeruginosa (strain ATCC 15692 / DSM 22644 / CIP 104116 / JCM 14847 / LMG 12228 / 1C / PRS 101 / PAO1) GN=wbpD PE=1 SV=1 | 119.3 |
| tr\|Q9HTI8\|Q9HTI8_PSEAE | Probable ATP-binding component of ABC transporter OS=Pseudomonas aeruginosa (strain ATCC 15692 / DSM 22644 / CIP 104116 / JCM 14847 / LMG 12228 / 1C / PRS 101 / PAO1) GN=PA5376 PE=4 SV=1 | 119.21 |
| tr\|Q9HTQ8\|Q9HTQ8_PSEAE | ATP-dependent DNA helicase Rep OS=Pseudomonas aeruginosa (strain ATCC 15692 / DSM 22644 / CIP 104116 / JCM 14847 / LMG 12228 / 1C / PRS 101 / PAO1) GN=rep PE=3 SV=1 | 118.27 |
| Q9HWF7\|RS13_PSEAE | 30S ribosomal protein S13 OS=Pseudomonas aeruginosa (strain ATCC 15692 / DSM 22644 / CIP 104116 / JCM 14847 / LMG 12228 / 1C / PRS 101 / PAO1) GN=rpsM PE=3 SV=1 | 117.69 |
| Q9I6C8\|CALB_PSEAE | Probable coniferyl aldehyde dehydrogenase OS=Pseudomonas aeruginosa (strain ATCC 15692 / DSM 22644 / CIP 104116 / JCM 14847 / LMG 12228 / 1C / PRS 101 / PAO1) GN=calB PE=3 SV=1 | 117.41 |
| tr\|Q9I3X2\|Q9I3X2_PSEAE | DNA helicase OS=Pseudomonas aeruginosa (strain ATCC 15692 / DSM 22644 / CIP 104116 / JCM 14847 / LMG 12228 / 1C / PRS 101 / PAO1) GN=PA1372 PE=4 SV=1 | 116.98 |
| Q9HXZ4\|PYRG_PSEAE | CTP synthase OS=Pseudomonas aeruginosa (strain ATCC 15692 / DSM 22644 / CIP 104116 / JCM 14847 / LMG 12228 / 1C / PRS 101 / PAO1) GN=pyrG PE=3 SV=1 | 116.91 |
| Q9HWF3\|RL30_PSEAE | 50S ribosomal protein L30 OS=Pseudomonas aeruginosa (strain ATCC 15692 / DSM 22644 / CIP 104116 / JCM 14847 / LMG 12228 / 1C / PRS 101 / PAO1) GN=rpmD PE=1 SV=3 | 116.58 |
| O30506\|AOTP_PSEAE | Arginine/ornithine transport ATP-binding protein AotP OS=Pseudomonas aeruginosa (strain ATCC 15692 / DSM 22644 / CIP 104116 / JCM 14847 / LMG 12228 / 1C / PRS 101 / PAO1) GN=aotP PE=3 SV=2 | 116.5 |
| Q9I7B8\|SYGB_PSEAE | Glycine--tRNA ligase beta subunit OS=Pseudomonas aeruginosa (strain ATCC 15692 / DSM 22644 / CIP 104116 / JCM 14847 / LMG 12228 / 1C / PRS 101 / PAO1) GN=glyS PE=3 SV=1 | 116.42 |
| tr\|Q9HZG8\|Q9HZG8_PSEAE | Uncharacterized protein OS=Pseudomonas aeruginosa (strain ATCC 15692 / DSM 22644 / CIP 104116 / JCM 14847 / LMG 12228 / 1C / PRS 101 / PAO1) GN=PA3040 PE=4 SV=1 | 116.16 |
| Q9HUF1\|MSRA_PSEAE | Peptide methionine sulfoxide reductase MsrA OS=Pseudomonas aeruginosa (strain ATCC 15692 / DSM 22644 / CIP 104116 / JCM 14847 / LMG 12228 / 1C / PRS 101 / PAO1) GN=msrA PE=3 SV=1 | 115.87 |
| Q9HW01\|MURG_PSEAE | UDP-N-acetylglucosamine--N-acetylmuramyl-(pentapeptide) pyrophosphoryl-undecaprenol N-acetylglucosamine transferase OS=Pseudomonas aeruginosa (strain ATCC 15692 / DSM 22644 / CIP 104116 / JCM 14847 / LMG 12228 / 1C / PRS 101 / PAO1) ... | 115.45 |
| tr\|Q9HY77\|Q9HY77_PSEAE | Glutaredoxin OS=Pseudomonas aeruginosa (strain ATCC 15692 / DSM 22644 / CIP 104116 / JCM 14847 / LMG 12228 / 1C / PRS 101 / PAO1) GN=PA3533 PE=3 SV=1 | 114.67 |
| Q9HUN2\|RL9_PSEAE | 50S ribosomal protein L9 OS=Pseudomonas aeruginosa (strain ATCC 15692 / DSM 22644 / CIP 104116 / JCM 14847 / LMG 12228 / 1C / PRS 101 / PAO1) GN=rplI PE=3 SV=1 | 114.07 |
| Q51481\|NIRQ_PSEAE | Denitrification regulatory protein NirQ OS=Pseudomonas aeruginosa (strain ATCC 15692 / DSM 22644 / CIP 104116 / JCM 14847 / LMG 12228 / 1C / PRS 101 / PAO1) GN=nirQ PE=2 SV=1 | 113.78 |
| tr\|Q9HXT9\|Q9HXT9_PSEAE | Probable two-component response regulator OS=Pseudomonas aeruginosa (strain ATCC 15692 / DSM 22644 / CIP 104116 / JCM 14847 / LMG 12228 / 1C / PRS 101 / PAO1) GN=wspR PE=1 SV=1 | 113.43 |
| Q00934\|PILR_PSEAE | Type 4 fimbriae expression regulatory protein PilR OS=Pseudomonas aeruginosa (strain ATCC 15692 / DSM 22644 / CIP 104116 / JCM 14847 / LMG 12228 / 1C / PRS 101 / PAO1) GN=pilR PE=3 SV=2 | 113.31 |
| Q9I0J7\|NUOF_PSEAE | NADH-quinone oxidoreductase subunit F OS=Pseudomonas aeruginosa (strain ATCC 15692 / DSM 22644 / CIP 104116 / JCM 14847 / LMG 12228 / 1C / PRS 101 / PAO1) GN=nuoF PE=3 SV=1 | 113.22 |
| Q9HWF4\|RL15_PSEAE | 50S ribosomal protein L15 OS=Pseudomonas aeruginosa (strain ATCC 15692 / DSM 22644 / CIP 104116 / JCM 14847 / LMG 12228 / 1C / PRS 101 / PAO1) GN=rplO PE=3 SV=1 | 112.95 |
| tr\|Q9HWW1\|Q9HWW1_PSEAE | Outer membrane protein OprG OS=Pseudomonas aeruginosa (strain ATCC 15692 / DSM 22644 / CIP 104116 / JCM 14847 / LMG 12228 / 1C / PRS 101 / PAO1) GN=oprG PE=1 SV=1 | 112.89 |
| Q9HZA3\|LEUC_PSEAE | 3-isopropylmalate dehydratase large subunit OS=Pseudomonas aeruginosa (strain ATCC 15692 / DSM 22644 / CIP 104116 / JCM 14847 / LMG 12228 / 1C / PRS 101 / PAO1) GN=leuC PE=3 SV=1 | 112.89 |
| P15276\|ALGP_PSEAE | Transcriptional regulatory protein AlgP OS=Pseudomonas aeruginosa (strain ATCC 15692 / DSM 22644 / CIP 104116 / JCM 14847 / LMG 12228 / 1C / PRS 101 / PAO1) GN=algP PE=4 SV=2 | 112.61 |
| Q9HVY3\|RS9_PSEAE | 30S ribosomal protein S9 OS=Pseudomonas aeruginosa (strain ATCC 15692 / DSM 22644 / CIP 104116 / JCM 14847 / LMG 12228 / 1C / PRS 101 / PAO1) GN=rpsI PE=3 SV=1 | 111.83 |
| P20576\|TRPG_PSEAE | Anthranilate synthase component 2 OS=Pseudomonas aeruginosa (strain ATCC 15692 / DSM 22644 / CIP 104116 / JCM 14847 / LMG 12228 / 1C / PRS 101 / PAO1) GN=trpG PE=4 SV=2 | 110.74 |
| tr\|Q9HW06\|Q9HW06_PSEAE | Probable pyrophosphohydrolase OS=Pseudomonas aeruginosa (strain ATCC 15692 / DSM 22644 / CIP 104116 / JCM 14847 / LMG 12228 / 1C / PRS 101 / PAO1) GN=PA4400 PE=4 SV=1 | 110.37 |
| tr\|G3XCT6\|G3XCT6_PSEAE | Probable two-component sensor OS=Pseudomonas aeruginosa (strain ATCC 15692 / DSM 22644 / CIP 104116 / JCM 14847 / LMG 12228 / 1C / PRS 101 / PAO1) GN=PA1458 PE=4 SV=1 | 109.88 |
| tr\|Q9I4F9\|Q9I4F9_PSEAE | Two-component response regulator PhoP OS=Pseudomonas aeruginosa (strain ATCC 15692 / DSM 22644 / CIP 104116 / JCM 14847 / LMG 12228 / 1C / PRS 101 / PAO1) GN=phoP PE=4 SV=1 | 109.67 |
| tr\|Q9I632\|Q9I632_PSEAE | Stress response kinase A OS=Pseudomonas aeruginosa (strain ATCC 15692 / DSM 22644 / CIP 104116 / JCM 14847 / LMG 12228 / 1C / PRS 101 / PAO1) GN=srkA PE=3 SV=1 | 109.53 |
| Q9I2U2\|TIG_PSEAE | Trigger factor OS=Pseudomonas aeruginosa (strain ATCC 15692 / DSM 22644 / CIP 104116 / JCM 14847 / LMG 12228 / 1C / PRS 101 / PAO1) GN=tig PE=3 SV=1 | 109.32 |
| Q9I3C5\|HTPG_PSEAE | Chaperone protein HtpG OS=Pseudomonas aeruginosa (strain ATCC 15692 / DSM 22644 / CIP 104116 / JCM 14847 / LMG 12228 / 1C / PRS 101 / PAO1) GN=htpG PE=3 SV=1 | 108.74 |
| tr\|Q9HUE1\|Q9HUE1_PSEAE | Uncharacterized protein OS=Pseudomonas aeruginosa (strain ATCC 15692 / DSM 22644 / CIP 104116 / JCM 14847 / LMG 12228 / 1C / PRS 101 / PAO1) GN=PA5028 PE=4 SV=1 | 108.64 |
| Q9I6J2\|SPUC_PSEAE | Putrescine--pyruvate aminotransferase OS=Pseudomonas aeruginosa (strain ATCC 15692 / DSM 22644 / CIP 104116 / JCM 14847 / LMG 12228 / 1C / PRS 101 / PAO1) GN=spuC PE=1 SV=1 | 107.6 |
| Q9HVA2\|ILVC_PSEAE | Ketol-acid reductoisomerase (NADP(+)) OS=Pseudomonas aeruginosa (strain ATCC 15692 / DSM 22644 / CIP 104116 / JCM 14847 / LMG 12228 / 1C / PRS 101 / PAO1) GN=ilvC PE=1 SV=1 | 107.08 |
| tr\|Q9HVY6\|Q9HVY6_PSEAE | Probable cytochrome c1 OS=Pseudomonas aeruginosa (strain ATCC 15692 / DSM 22644 / CIP 104116 / JCM 14847 / LMG 12228 / 1C / PRS 101 / PAO1) GN=PA4429 PE=4 SV=1 | 105.72 |
| P20582\|PQSD_PSEAE | Anthraniloyl-CoA anthraniloyltransferase OS=Pseudomonas aeruginosa (strain ATCC 15692 / DSM 22644 / CIP 104116 / JCM 14847 / LMG 12228 / 1C / PRS 101 / PAO1) GN=pqsD PE=1 SV=2 | 104.92 |
| tr\|Q9HYU8\|Q9HYU8_PSEAE | Probable HIT family protein OS=Pseudomonas aeruginosa (strain ATCC 15692 / DSM 22644 / CIP 104116 / JCM 14847 / LMG 12228 / 1C / PRS 101 / PAO1) GN=PA3295 PE=4 SV=1 | 103.47 |
| Q9HXZ2\|ACCA_PSEAE | Acetyl-coenzyme A carboxylase carboxyl transferase subunit alpha OS=Pseudomonas aeruginosa (strain ATCC 15692 / DSM 22644 / CIP 104116 / JCM 14847 / LMG 12228 / 1C / PRS 101 / PAO1) GN=accA PE=1 SV=1 | 103.06 |
| Q9HVC5\|KPRS_PSEAE | Ribose-phosphate pyrophosphokinase OS=Pseudomonas aeruginosa (strain ATCC 15692 / DSM 22644 / CIP 104116 / JCM 14847 / LMG 12228 / 1C / PRS 101 / PAO1) GN=prs PE=3 SV=1 | 103.02 |
| Q9HUF4\|GLNE_PSEAE | Bifunctional glutamine synthetase adenylyltransferase/adenylyl-removing enzyme OS=Pseudomonas aeruginosa (strain ATCC 15692 / DSM 22644 / CIP 104116 / JCM 14847 / LMG 12228 / 1C / PRS 101 / PAO1) GN=glnE PE=3 SV=1 | 103 |
| tr\|Q9HX42\|Q9HX42_PSEAE | Lost Adherence Sensor LadS OS=Pseudomonas aeruginosa (strain ATCC 15692 / DSM 22644 / CIP 104116 / JCM 14847 / LMG 12228 / 1C / PRS 101 / PAO1) GN=ladS PE=4 SV=1 | 102.52 |
| Q9I0H4\|HMP_PSEAE | Flavohemoprotein OS=Pseudomonas aeruginosa (strain ATCC 15692 / DSM 22644 / CIP 104116 / JCM 14847 / LMG 12228 / 1C / PRS 101 / PAO1) GN=hmp PE=3 SV=1 | 102.32 |
| Q59637\|ODP1_PSEAE | Pyruvate dehydrogenase E1 component OS=Pseudomonas aeruginosa (strain ATCC 15692 / DSM 22644 / CIP 104116 / JCM 14847 / LMG 12228 / 1C / PRS 101 / PAO1) GN=aceE PE=3 SV=2 | 102.27 |
| tr\|Q9HV96\|Q9HV96_PSEAE | Uncharacterized protein OS=Pseudomonas aeruginosa (strain ATCC 15692 / DSM 22644 / CIP 104116 / JCM 14847 / LMG 12228 / 1C / PRS 101 / PAO1) GN=PA4701 PE=4 SV=1 | 101.94 |
| tr\|Q9HTC6\|Q9HTC6_PSEAE | Probable peptidase OS=Pseudomonas aeruginosa (strain ATCC 15692 / DSM 22644 / CIP 104116 / JCM 14847 / LMG 12228 / 1C / PRS 101 / PAO1) GN=PA5440 PE=4 SV=1 | 101.62 |
| tr\|Q9HYK7\|Q9HYK7_PSEAE | Ferredoxin--NADP+ reductase OS=Pseudomonas aeruginosa (strain ATCC 15692 / DSM 22644 / CIP 104116 / JCM 14847 / LMG 12228 / 1C / PRS 101 / PAO1) GN=fpr PE=1 SV=1 | 101.49 |
| Q9I3W8\|ACEK_PSEAE | Isocitrate dehydrogenase kinase/phosphatase OS=Pseudomonas aeruginosa (strain ATCC 15692 / DSM 22644 / CIP 104116 / JCM 14847 / LMG 12228 / 1C / PRS 101 / PAO1) GN=aceK PE=3 SV=1 | 101.36 |
| tr\|Q9I1N6\|Q9I1N6_PSEAE | PslC OS=Pseudomonas aeruginosa (strain ATCC 15692 / DSM 22644 / CIP 104116 / JCM 14847 / LMG 12228 / 1C / PRS 101 / PAO1) GN=pslC PE=4 SV=1 | 101.11 |
| tr\|Q9HUC3\|Q9HUC3_PSEAE | Polyhydroxyalkanoate synthesis protein PhaF OS=Pseudomonas aeruginosa (strain ATCC 15692 / DSM 22644 / CIP 104116 / JCM 14847 / LMG 12228 / 1C / PRS 101 / PAO1) GN=phaF PE=4 SV=1 | 100.54 |
| Q9HXJ4\|ISPG_PSEAE | 4-hydroxy-3-methylbut-2-en-1-yl diphosphate synthase (flavodoxin) OS=Pseudomonas aeruginosa (strain ATCC 15692 / DSM 22644 / CIP 104116 / JCM 14847 / LMG 12228 / 1C / PRS 101 / PAO1) GN=ispG PE=3 SV=1 | 100.42 |
| Q9HT95\|ATSE4_PSEAE | Acetyltransferase PA5475 OS=Pseudomonas aeruginosa (strain ATCC 15692 / DSM 22644 / CIP 104116 / JCM 14847 / LMG 12228 / 1C / PRS 101 / PAO1) GN=PA5475 PE=4 SV=1 | 100.36 |
| P13982\|ARCC_PSEAE | Carbamate kinase OS=Pseudomonas aeruginosa (strain ATCC 15692 / DSM 22644 / CIP 104116 / JCM 14847 / LMG 12228 / 1C / PRS 101 / PAO1) GN=arcC PE=3 SV=1 | 100.09 |
| Q9HWD1\|RS7_PSEAE | 30S ribosomal protein S7 OS=Pseudomonas aeruginosa (strain ATCC 15692 / DSM 22644 / CIP 104116 / JCM 14847 / LMG 12228 / 1C / PRS 101 / PAO1) GN=rpsG PE=3 SV=1 | 99.71 |
| Q9HUP4\|PNCB1_PSEAE | Nicotinate phosphoribosyltransferase 1 OS=Pseudomonas aeruginosa (strain ATCC 15692 / DSM 22644 / CIP 104116 / JCM 14847 / LMG 12228 / 1C / PRS 101 / PAO1) GN=pncB1 PE=3 SV=1 | 99.26 |
| tr\|Q9HYR5\|Q9HYR5_PSEAE | Probable short chain dehydrogenase OS=Pseudomonas aeruginosa (strain ATCC 15692 / DSM 22644 / CIP 104116 / JCM 14847 / LMG 12228 / 1C / PRS 101 / PAO1) GN=PA3330 PE=4 SV=1 | 99.2 |
| tr\|Q9I762\|Q9I762_PSEAE | Uncharacterized protein OS=Pseudomonas aeruginosa (strain ATCC 15692 / DSM 22644 / CIP 104116 / JCM 14847 / LMG 12228 / 1C / PRS 101 / PAO1) GN=PA0070 PE=4 SV=1 | 98.09 |
| tr\|Q9HYZ6\|Q9HYZ6_PSEAE | Site-determining protein OS=Pseudomonas aeruginosa (strain ATCC 15692 / DSM 22644 / CIP 104116 / JCM 14847 / LMG 12228 / 1C / PRS 101 / PAO1) GN=minD PE=3 SV=1 | 97.35 |
| Q9HTQ0\|DADA1_PSEAE | D-amino acid dehydrogenase 1 OS=Pseudomonas aeruginosa (strain ATCC 15692 / DSM 22644 / CIP 104116 / JCM 14847 / LMG 12228 / 1C / PRS 101 / PAO1) GN=dadA1 PE=1 SV=1 | 97.16 |
| Q9HWE0\|RL22_PSEAE | 50S ribosomal protein L22 OS=Pseudomonas aeruginosa (strain ATCC 15692 / DSM 22644 / CIP 104116 / JCM 14847 / LMG 12228 / 1C / PRS 101 / PAO1) GN=rplV PE=3 SV=1 | 96.37 |
| P29364\|KHSE_PSEAE | Homoserine kinase OS=Pseudomonas aeruginosa (strain ATCC 15692 / DSM 22644 / CIP 104116 / JCM 14847 / LMG 12228 / 1C / PRS 101 / PAO1) GN=thrB PE=3 SV=2 | 95.06 |
| tr\|Q9HXC2\|Q9HXC2_PSEAE | Probable ATP-binding component of ABC transporter OS=Pseudomonas aeruginosa (strain ATCC 15692 / DSM 22644 / CIP 104116 / JCM 14847 / LMG 12228 / 1C / PRS 101 / PAO1) GN=PA3891 PE=4 SV=1 | 94.57 |
| Q9HVV3\|Y4465_PSEAE | Nucleotide-binding protein PA4465 OS=Pseudomonas aeruginosa (strain ATCC 15692 / DSM 22644 / CIP 104116 / JCM 14847 / LMG 12228 / 1C / PRS 101 / PAO1) GN=PA4465 PE=3 SV=1 | 94.25 |
| tr\|G3XD64\|G3XD64_PSEAE | Site-determining protein OS=Pseudomonas aeruginosa (strain ATCC 15692 / DSM 22644 / CIP 104116 / JCM 14847 / LMG 12228 / 1C / PRS 101 / PAO1) GN=fleN PE=1 SV=1 | 93.4 |
| tr\|Q9HZ78\|Q9HZ78_PSEAE | LPS biosynthesis protein WbpG OS=Pseudomonas aeruginosa (strain ATCC 15692 / DSM 22644 / CIP 104116 / JCM 14847 / LMG 12228 / 1C / PRS 101 / PAO1) GN=wbpG PE=4 SV=1 | 93.35 |
| tr\|Q9I2H8\|Q9I2H8_PSEAE | Uncharacterized protein OS=Pseudomonas aeruginosa (strain ATCC 15692 / DSM 22644 / CIP 104116 / JCM 14847 / LMG 12228 / 1C / PRS 101 / PAO1) GN=PA1926 PE=4 SV=1 | 93.24 |
| tr\|Q9I4R3\|Q9I4R3_PSEAE | Uncharacterized protein OS=Pseudomonas aeruginosa (strain ATCC 15692 / DSM 22644 / CIP 104116 / JCM 14847 / LMG 12228 / 1C / PRS 101 / PAO1) GN=PA1061 PE=4 SV=1 | 92.82 |
| tr\|Q9I0L8\|Q9I0L8_PSEAE | ATP-binding protease component ClpA OS=Pseudomonas aeruginosa (strain ATCC 15692 / DSM 22644 / CIP 104116 / JCM 14847 / LMG 12228 / 1C / PRS 101 / PAO1) GN=clpA PE=3 SV=1 | 92.63 |
| tr\|Q9I6I9\|Q9I6I9_PSEAE | Spermidine/putrescine import ATP-binding protein PotA OS=Pseudomonas aeruginosa (strain ATCC 15692 / DSM 22644 / CIP 104116 / JCM 14847 / LMG 12228 / 1C / PRS 101 / PAO1) GN=spuF PE=3 SV=1 | 92.61 |
| tr\|Q9HTU2\|Q9HTU2_PSEAE | Probable ATP-binding component of ABC transporter OS=Pseudomonas aeruginosa (strain ATCC 15692 / DSM 22644 / CIP 104116 / JCM 14847 / LMG 12228 / 1C / PRS 101 / PAO1) GN=PA5252 PE=4 SV=1 | 91.49 |
| tr\|C6JW54\|C6JW54_PSEAE | Uncharacterized protein OS=Pseudomonas aeruginosa (strain ATCC 15692 / DSM 22644 / CIP 104116 / JCM 14847 / LMG 12228 / 1C / PRS 101 / PAO1) GN=PA4673.16 PE=4 SV=1 | 91.42 |
| O82852\|PYRH_PSEAE | Uridylate kinase OS=Pseudomonas aeruginosa (strain ATCC 15692 / DSM 22644 / CIP 104116 / JCM 14847 / LMG 12228 / 1C / PRS 101 / PAO1) GN=pyrH PE=3 SV=2 | 91.18 |
| Q9HXJ5\|SYH_PSEAE | Histidine--tRNA ligase OS=Pseudomonas aeruginosa (strain ATCC 15692 / DSM 22644 / CIP 104116 / JCM 14847 / LMG 12228 / 1C / PRS 101 / PAO1) GN=hisS PE=3 SV=1 | 91.14 |
| tr\|Q9HV73\|Q9HV73_PSEAE | Two-component response regulator CbrB OS=Pseudomonas aeruginosa (strain ATCC 15692 / DSM 22644 / CIP 104116 / JCM 14847 / LMG 12228 / 1C / PRS 101 / PAO1) GN=cbrB PE=4 SV=1 | 90.76 |
| P53593\|SUCC_PSEAE | Succinate--CoA ligase [ADP-forming] subunit beta OS=Pseudomonas aeruginosa (strain ATCC 15692 / DSM 22644 / CIP 104116 / JCM 14847 / LMG 12228 / 1C / PRS 101 / PAO1) GN=sucC PE=1 SV=2 | 90.2 |
| P48247\|GSA_PSEAE | Glutamate-1-semialdehyde 2 1-aminomutase OS=Pseudomonas aeruginosa (strain ATCC 15692 / DSM 22644 / CIP 104116 / JCM 14847 / LMG 12228 / 1C / PRS 101 / PAO1) GN=hemL PE=1 SV=2 | 89.98 |
| tr\|G3XCW5\|G3XCW5_PSEAE | Uncharacterized protein OS=Pseudomonas aeruginosa (strain ATCC 15692 / DSM 22644 / CIP 104116 / JCM 14847 / LMG 12228 / 1C / PRS 101 / PAO1) GN=PA3031 PE=4 SV=1 | 89.88 |
| Q9HTF1\|LTAE_PSEAE | Low specificity L-threonine aldolase OS=Pseudomonas aeruginosa (strain ATCC 15692 / DSM 22644 / CIP 104116 / JCM 14847 / LMG 12228 / 1C / PRS 101 / PAO1) GN=ltaE PE=3 SV=1 | 89.83 |
| Q9I5A5\|PTA_PSEAE | Phosphate acetyltransferase OS=Pseudomonas aeruginosa (strain ATCC 15692 / DSM 22644 / CIP 104116 / JCM 14847 / LMG 12228 / 1C / PRS 101 / PAO1) GN=pta PE=1 SV=1 | 89.81 |
| Q9HVY2\|RL13_PSEAE | 50S ribosomal protein L13 OS=Pseudomonas aeruginosa (strain ATCC 15692 / DSM 22644 / CIP 104116 / JCM 14847 / LMG 12228 / 1C / PRS 101 / PAO1) GN=rplM PE=3 SV=1 | 89.78 |
| tr\|Q9HV76\|Q9HV76_PSEAE | Aminotransferase OS=Pseudomonas aeruginosa (strain ATCC 15692 / DSM 22644 / CIP 104116 / JCM 14847 / LMG 12228 / 1C / PRS 101 / PAO1) GN=PA4722 PE=3 SV=1 | 88.65 |
| tr\|Q9HYX8\|Q9HYX8_PSEAE | Peptidyl-prolyl cis-trans isomerase OS=Pseudomonas aeruginosa (strain ATCC 15692 / DSM 22644 / CIP 104116 / JCM 14847 / LMG 12228 / 1C / PRS 101 / PAO1) GN=PA3262 PE=3 SV=1 | 88.11 |
| tr\|Q9HTL3\|Q9HTL3_PSEAE | ATP-dependent DNA helicase RecG OS=Pseudomonas aeruginosa (strain ATCC 15692 / DSM 22644 / CIP 104116 / JCM 14847 / LMG 12228 / 1C / PRS 101 / PAO1) GN=recG PE=3 SV=1 | 88.08 |
| tr\|Q9I2R2\|Q9I2R2_PSEAE | Probable oxidoreductase OS=Pseudomonas aeruginosa (strain ATCC 15692 / DSM 22644 / CIP 104116 / JCM 14847 / LMG 12228 / 1C / PRS 101 / PAO1) GN=PA1833 PE=4 SV=1 | 87.97 |
| tr\|Q9HZG2\|Q9HZG2_PSEAE | Uncharacterized protein OS=Pseudomonas aeruginosa (strain ATCC 15692 / DSM 22644 / CIP 104116 / JCM 14847 / LMG 12228 / 1C / PRS 101 / PAO1) GN=PA3046 PE=4 SV=1 | 87.59 |
| Q9I2X0\|PSRP_PSEAE | Putative phosphoenolpyruvate synthase regulatory protein OS=Pseudomonas aeruginosa (strain ATCC 15692 / DSM 22644 / CIP 104116 / JCM 14847 / LMG 12228 / 1C / PRS 101 / PAO1) GN=PA1769 PE=3 SV=1 | 87.54 |
| tr\|Q9I2T9\|Q9I2T9_PSEAE | Lon protease OS=Pseudomonas aeruginosa (strain ATCC 15692 / DSM 22644 / CIP 104116 / JCM 14847 / LMG 12228 / 1C / PRS 101 / PAO1) GN=lon PE=2 SV=1 | 86.63 |
| tr\|Q9HUN3\|Q9HUN3_PSEAE | Replicative DNA helicase OS=Pseudomonas aeruginosa (strain ATCC 15692 / DSM 22644 / CIP 104116 / JCM 14847 / LMG 12228 / 1C / PRS 101 / PAO1) GN=dnaB PE=3 SV=1 | 86.51 |
| tr\|Q9HUK6\|Q9HUK6_PSEAE | FimX OS=Pseudomonas aeruginosa (strain ATCC 15692 / DSM 22644 / CIP 104116 / JCM 14847 / LMG 12228 / 1C / PRS 101 / PAO1) GN=fimX PE=1 SV=1 | 86.29 |
| Q9I5V8\|RS21_PSEAE | 30S ribosomal protein S21 OS=Pseudomonas aeruginosa (strain ATCC 15692 / DSM 22644 / CIP 104116 / JCM 14847 / LMG 12228 / 1C / PRS 101 / PAO1) GN=rpsU PE=1 SV=3 | 86.24 |
| tr\|Q9I6C0\|Q9I6C0_PSEAE | Cell division protein FtsE OS=Pseudomonas aeruginosa (strain ATCC 15692 / DSM 22644 / CIP 104116 / JCM 14847 / LMG 12228 / 1C / PRS 101 / PAO1) GN=ftsE PE=4 SV=1 | 85.89 |
| Q9K3C5\|FLID2_PSEAE | B-type flagellar hook-associated protein 2 OS=Pseudomonas aeruginosa (strain ATCC 15692 / DSM 22644 / CIP 104116 / JCM 14847 / LMG 12228 / 1C / PRS 101 / PAO1) GN=fliD PE=1 SV=1 | 85.33 |
| tr\|E1JGJ8\|E1JGJ8_PSEAE | Peptide chain release factor 2 OS=Pseudomonas aeruginosa (strain ATCC 15692 / DSM 22644 / CIP 104116 / JCM 14847 / LMG 12228 / 1C / PRS 101 / PAO1) GN=prfB PE=3 SV=1 | 84.88 |
| tr\|Q9I0D7\|Q9I0D7_PSEAE | Uncharacterized protein OS=Pseudomonas aeruginosa (strain ATCC 15692 / DSM 22644 / CIP 104116 / JCM 14847 / LMG 12228 / 1C / PRS 101 / PAO1) GN=PA2705 PE=4 SV=1 | 84.59 |
| Q9HW02\|MURC_PSEAE | UDP-N-acetylmuramate--L-alanine ligase OS=Pseudomonas aeruginosa (strain ATCC 15692 / DSM 22644 / CIP 104116 / JCM 14847 / LMG 12228 / 1C / PRS 101 / PAO1) GN=murC PE=1 SV=1 | 83.81 |
| tr\|Q9I2E0\|Q9I2E0_PSEAE | Probable ATP-binding component of ABC transporter OS=Pseudomonas aeruginosa (strain ATCC 15692 / DSM 22644 / CIP 104116 / JCM 14847 / LMG 12228 / 1C / PRS 101 / PAO1) GN=PA1964 PE=4 SV=1 | 83.14 |
| P40947\|SSB_PSEAE | Single-stranded DNA-binding protein OS=Pseudomonas aeruginosa (strain ATCC 15692 / DSM 22644 / CIP 104116 / JCM 14847 / LMG 12228 / 1C / PRS 101 / PAO1) GN=ssb PE=1 SV=3 | 83.13 |
| tr\|Q9HV54\|Q9HV54_PSEAE | Transcription termination/antitermination protein NusA OS=Pseudomonas aeruginosa (strain ATCC 15692 / DSM 22644 / CIP 104116 / JCM 14847 / LMG 12228 / 1C / PRS 101 / PAO1) GN=nusA PE=3 SV=1 | 82.76 |
| Q9HYT6\|RAPA_PSEAE | RNA polymerase-associated protein RapA OS=Pseudomonas aeruginosa (strain ATCC 15692 / DSM 22644 / CIP 104116 / JCM 14847 / LMG 12228 / 1C / PRS 101 / PAO1) GN=rapA PE=3 SV=1 | 82.01 |
| tr\|Q9HUL5\|Q9HUL5_PSEAE | Multifunctional fusion protein OS=Pseudomonas aeruginosa (strain ATCC 15692 / DSM 22644 / CIP 104116 / JCM 14847 / LMG 12228 / 1C / PRS 101 / PAO1) GN=nnrD PE=3 SV=1 | 81.72 |
| Q9I0L2\|MNMA_PSEAE | tRNA-specific 2-thiouridylase MnmA OS=Pseudomonas aeruginosa (strain ATCC 15692 / DSM 22644 / CIP 104116 / JCM 14847 / LMG 12228 / 1C / PRS 101 / PAO1) GN=mnmA PE=3 SV=1 | 81.64 |
| tr\|Q9I0L6\|Q9I0L6_PSEAE | Cold-shock protein CspD OS=Pseudomonas aeruginosa (strain ATCC 15692 / DSM 22644 / CIP 104116 / JCM 14847 / LMG 12228 / 1C / PRS 101 / PAO1) GN=cspD PE=4 SV=1 | 81.62 |
| Q9I2P8\|NFUA_PSEAE | Fe/S biogenesis protein NfuA OS=Pseudomonas aeruginosa (strain ATCC 15692 / DSM 22644 / CIP 104116 / JCM 14847 / LMG 12228 / 1C / PRS 101 / PAO1) GN=nfuA PE=3 SV=1 | 80.98 |
| tr\|Q9I5F9\|Q9I5F9_PSEAE | Lon protease OS=Pseudomonas aeruginosa (strain ATCC 15692 / DSM 22644 / CIP 104116 / JCM 14847 / LMG 12228 / 1C / PRS 101 / PAO1) GN=lon PE=2 SV=1 | 80.21 |
| Q9HU41\|HIS7_PSEAE | Imidazoleglycerol-phosphate dehydratase OS=Pseudomonas aeruginosa (strain ATCC 15692 / DSM 22644 / CIP 104116 / JCM 14847 / LMG 12228 / 1C / PRS 101 / PAO1) GN=hisB PE=3 SV=1 | 79.44 |
| P25084\|LASR_PSEAE | Transcriptional activator protein LasR OS=Pseudomonas aeruginosa (strain ATCC 15692 / DSM 22644 / CIP 104116 / JCM 14847 / LMG 12228 / 1C / PRS 101 / PAO1) GN=lasR PE=1 SV=1 | 79.43 |
| tr\|Q9HW86\|Q9HW86_PSEAE | MvaT OS=Pseudomonas aeruginosa (strain ATCC 15692 / DSM 22644 / CIP 104116 / JCM 14847 / LMG 12228 / 1C / PRS 101 / PAO1) GN=mvaT PE=1 SV=1 | 79.22 |
| P72139\|HIS62_PSEAE | Putative imidazole glycerol phosphate synthase subunit hisF2 OS=Pseudomonas aeruginosa (strain ATCC 15692 / DSM 22644 / CIP 104116 / JCM 14847 / LMG 12228 / 1C / PRS 101 / PAO1) GN=hisF2 PE=3 SV=1 | 79.04 |
| tr\|Q9I624\|Q9I624_PSEAE | Probable acyl-CoA carboxylase subunit OS=Pseudomonas aeruginosa (strain ATCC 15692 / DSM 22644 / CIP 104116 / JCM 14847 / LMG 12228 / 1C / PRS 101 / PAO1) GN=PA0494 PE=4 SV=1 | 78.06 |
| Q9HXT8\|CHEB3_PSEAE | Chemotaxis response regulator protein-glutamate methylesterase of group 3 operon OS=Pseudomonas aeruginosa (strain ATCC 15692 / DSM 22644 / CIP 104116 / JCM 14847 / LMG 12228 / 1C / PRS 101 / PAO1) GN=cheB3 PE=3 SV=1 | 77.67 |
| P24474\|NIRS_PSEAE | Nitrite reductase OS=Pseudomonas aeruginosa (strain ATCC 15692 / DSM 22644 / CIP 104116 / JCM 14847 / LMG 12228 / 1C / PRS 101 / PAO1) GN=nirS PE=1 SV=1 | 77.6 |
| Q9HWF9\|BFR_PSEAE | Bacterioferritin OS=Pseudomonas aeruginosa (strain ATCC 15692 / DSM 22644 / CIP 104116 / JCM 14847 / LMG 12228 / 1C / PRS 101 / PAO1) GN=bfr PE=1 SV=1 | 77.34 |
| Q9ZN70\|PPX_PSEAE | Exopolyphosphatase OS=Pseudomonas aeruginosa (strain ATCC 15692 / DSM 22644 / CIP 104116 / JCM 14847 / LMG 12228 / 1C / PRS 101 / PAO1) GN=ppx PE=1 SV=1 | 77.26 |
| tr\|Q9HWW9\|Q9HWW9_PSEAE | Uncharacterized protein OS=Pseudomonas aeruginosa (strain ATCC 15692 / DSM 22644 / CIP 104116 / JCM 14847 / LMG 12228 / 1C / PRS 101 / PAO1) GN=PA4059 PE=4 SV=1 | 76.92 |
| Q9HV52\|SECG_PSEAE | Protein-export membrane protein SecG OS=Pseudomonas aeruginosa (strain ATCC 15692 / DSM 22644 / CIP 104116 / JCM 14847 / LMG 12228 / 1C / PRS 101 / PAO1) GN=secG PE=3 SV=1 | 76.91 |
| Q59653\|PYRB_PSEAE | Aspartate carbamoyltransferase OS=Pseudomonas aeruginosa (strain ATCC 15692 / DSM 22644 / CIP 104116 / JCM 14847 / LMG 12228 / 1C / PRS 101 / PAO1) GN=pyrB PE=3 SV=2 | 76.87 |
| tr\|Q9HTY0\|Q9HTY0_PSEAE | Probable secretion pathway ATPase OS=Pseudomonas aeruginosa (strain ATCC 15692 / DSM 22644 / CIP 104116 / JCM 14847 / LMG 12228 / 1C / PRS 101 / PAO1) GN=PA5210 PE=4 SV=1 | 76.67 |
| Q9HTN8\|RL28_PSEAE | 50S ribosomal protein L28 OS=Pseudomonas aeruginosa (strain ATCC 15692 / DSM 22644 / CIP 104116 / JCM 14847 / LMG 12228 / 1C / PRS 101 / PAO1) GN=rpmB PE=3 SV=1 | 75.74 |
| Q9HT70\|METN2_PSEAE | Methionine import ATP-binding protein MetN 2 OS=Pseudomonas aeruginosa (strain ATCC 15692 / DSM 22644 / CIP 104116 / JCM 14847 / LMG 12228 / 1C / PRS 101 / PAO1) GN=metN2 PE=3 SV=1 | 75.71 |
| Q9HVD1\|PAGL_PSEAE | Lipid A deacylase PagL OS=Pseudomonas aeruginosa (strain ATCC 15692 / DSM 22644 / CIP 104116 / JCM 14847 / LMG 12228 / 1C / PRS 101 / PAO1) GN=pagL PE=1 SV=1 | 74.07 |
| tr\|Q9HU74\|Q9HU74_PSEAE | Uncharacterized protein OS=Pseudomonas aeruginosa (strain ATCC 15692 / DSM 22644 / CIP 104116 / JCM 14847 / LMG 12228 / 1C / PRS 101 / PAO1) GN=PA5109 PE=4 SV=1 | 73.95 |
| Q9I5Q6\|ERPA_PSEAE | Iron-sulfur cluster insertion protein ErpA OS=Pseudomonas aeruginosa (strain ATCC 15692 / DSM 22644 / CIP 104116 / JCM 14847 / LMG 12228 / 1C / PRS 101 / PAO1) GN=erpA PE=3 SV=1 | 73.87 |
| Q9HV55\|IF2_PSEAE | Translation initiation factor IF-2 OS=Pseudomonas aeruginosa (strain ATCC 15692 / DSM 22644 / CIP 104116 / JCM 14847 / LMG 12228 / 1C / PRS 101 / PAO1) GN=infB PE=3 SV=1 | 73.11 |
| tr\|Q9HVS8\|Q9HVS8_PSEAE | RoxR OS=Pseudomonas aeruginosa (strain ATCC 15692 / DSM 22644 / CIP 104116 / JCM 14847 / LMG 12228 / 1C / PRS 101 / PAO1) GN=roxR PE=4 SV=1 | 72.68 |
| tr\|Q9I2S3\|Q9I2S3_PSEAE | Uncharacterized protein OS=Pseudomonas aeruginosa (strain ATCC 15692 / DSM 22644 / CIP 104116 / JCM 14847 / LMG 12228 / 1C / PRS 101 / PAO1) GN=fimL PE=4 SV=1 | 72.18 |
| tr\|Q9HZ61\|Q9HZ61_PSEAE | Probable short-chain dehydrogenase OS=Pseudomonas aeruginosa (strain ATCC 15692 / DSM 22644 / CIP 104116 / JCM 14847 / LMG 12228 / 1C / PRS 101 / PAO1) GN=PA3173 PE=4 SV=1 | 71.99 |
| tr\|Q9I3G6\|Q9I3G6_PSEAE | Probable ferredoxin OS=Pseudomonas aeruginosa (strain ATCC 15692 / DSM 22644 / CIP 104116 / JCM 14847 / LMG 12228 / 1C / PRS 101 / PAO1) GN=PA1551 PE=4 SV=1 | 71.43 |
| tr\|Q9I2X9\|Q9I2X9_PSEAE | Probable transcriptional regulator OS=Pseudomonas aeruginosa (strain ATCC 15692 / DSM 22644 / CIP 104116 / JCM 14847 / LMG 12228 / 1C / PRS 101 / PAO1) GN=PA1760 PE=4 SV=1 | 71.25 |
| G3XD94\|UGND_PSEAE | UDP-N-acetyl-D-glucosamine 6-dehydrogenase OS=Pseudomonas aeruginosa (strain ATCC 15692 / DSM 22644 / CIP 104116 / JCM 14847 / LMG 12228 / 1C / PRS 101 / PAO1) GN=wbpA PE=1 SV=1 | 71.13 |
| tr\|Q9I0R1\|Q9I0R1_PSEAE | Uncharacterized protein OS=Pseudomonas aeruginosa (strain ATCC 15692 / DSM 22644 / CIP 104116 / JCM 14847 / LMG 12228 / 1C / PRS 101 / PAO1) GN=PA2575 PE=4 SV=1 | 70.52 |
| tr\|Q9I0D5\|Q9I0D5_PSEAE | Uncharacterized protein OS=Pseudomonas aeruginosa (strain ATCC 15692 / DSM 22644 / CIP 104116 / JCM 14847 / LMG 12228 / 1C / PRS 101 / PAO1) GN=PA2707 PE=4 SV=1 | 70.11 |
| tr\|Q9I7B0\|Q9I7B0_PSEAE | Potassium uptake protein TrkA OS=Pseudomonas aeruginosa (strain ATCC 15692 / DSM 22644 / CIP 104116 / JCM 14847 / LMG 12228 / 1C / PRS 101 / PAO1) GN=trkA PE=4 SV=1 | 69.73 |
| Q00514\|GSPG_PSEAE | Type II secretion system protein G OS=Pseudomonas aeruginosa (strain ATCC 15692 / DSM 22644 / CIP 104116 / JCM 14847 / LMG 12228 / 1C / PRS 101 / PAO1) GN=xcpT PE=1 SV=1 | 69.71 |
| Q9HUU5\|URE1_PSEAE | Urease subunit alpha OS=Pseudomonas aeruginosa (strain ATCC 15692 / DSM 22644 / CIP 104116 / JCM 14847 / LMG 12228 / 1C / PRS 101 / PAO1) GN=ureC PE=3 SV=1 | 69.58 |
| tr\|Q9I3G4\|Q9I3G4_PSEAE | Cytochrome c oxidase cbb3-type CcoO subunit OS=Pseudomonas aeruginosa (strain ATCC 15692 / DSM 22644 / CIP 104116 / JCM 14847 / LMG 12228 / 1C / PRS 101 / PAO1) GN=ccoO1 PE=4 SV=1 | 69.5 |
| tr\|Q9I3G1\|Q9I3G1_PSEAE | Cytochrome c oxidase cbb3-type CcoO subunit OS=Pseudomonas aeruginosa (strain ATCC 15692 / DSM 22644 / CIP 104116 / JCM 14847 / LMG 12228 / 1C / PRS 101 / PAO1) GN=ccoO2 PE=4 SV=1 | 69.5 |
| tr\|Q9HVR5\|Q9HVR5_PSEAE | Probable ATP-binding component of ABC dipeptide transporter OS=Pseudomonas aeruginosa (strain ATCC 15692 / DSM 22644 / CIP 104116 / JCM 14847 / LMG 12228 / 1C / PRS 101 / PAO1) GN=PA4506 PE=3 SV=1 | 69.34 |
| O52761\|RL17_PSEAE | 50S ribosomal protein L17 OS=Pseudomonas aeruginosa (strain ATCC 15692 / DSM 22644 / CIP 104116 / JCM 14847 / LMG 12228 / 1C / PRS 101 / PAO1) GN=rplQ PE=3 SV=1 | 69.22 |
| Q9I4Z4\|PAL_PSEAE | Peptidoglycan-associated lipoprotein OS=Pseudomonas aeruginosa (strain ATCC 15692 / DSM 22644 / CIP 104116 / JCM 14847 / LMG 12228 / 1C / PRS 101 / PAO1) GN=pal PE=3 SV=1 | 69.21 |
| tr\|Q9I2U9\|Q9I2U9_PSEAE | Peptidyl-prolyl cis-trans isomerase OS=Pseudomonas aeruginosa (strain ATCC 15692 / DSM 22644 / CIP 104116 / JCM 14847 / LMG 12228 / 1C / PRS 101 / PAO1) GN=ppiB PE=3 SV=1 | 69.05 |
| Q9HVT8\|GATA_PSEAE | Glutamyl-tRNA(Gln) amidotransferase subunit A OS=Pseudomonas aeruginosa (strain ATCC 15692 / DSM 22644 / CIP 104116 / JCM 14847 / LMG 12228 / 1C / PRS 101 / PAO1) GN=gatA PE=1 SV=1 | 68.85 |
| tr\|Q9I0U7\|Q9I0U7_PSEAE | Probable acyltransferase OS=Pseudomonas aeruginosa (strain ATCC 15692 / DSM 22644 / CIP 104116 / JCM 14847 / LMG 12228 / 1C / PRS 101 / PAO1) GN=PA2537 PE=4 SV=1 | 68.73 |
| tr\|Q9HTS8\|Q9HTS8_PSEAE | Adenylate cyclase OS=Pseudomonas aeruginosa (strain ATCC 15692 / DSM 22644 / CIP 104116 / JCM 14847 / LMG 12228 / 1C / PRS 101 / PAO1) GN=cyaA PE=4 SV=1 | 68.72 |
| tr\|Q9HTP2\|Q9HTP2_PSEAE | Probable aldehyde dehydrogenase OS=Pseudomonas aeruginosa (strain ATCC 15692 / DSM 22644 / CIP 104116 / JCM 14847 / LMG 12228 / 1C / PRS 101 / PAO1) GN=PA5312 PE=3 SV=1 | 68.6 |
| Q9HX97\|MOAE_PSEAE | Molybdopterin synthase catalytic subunit OS=Pseudomonas aeruginosa (strain ATCC 15692 / DSM 22644 / CIP 104116 / JCM 14847 / LMG 12228 / 1C / PRS 101 / PAO1) GN=moaE PE=3 SV=1 | 68.48 |
| Q9HZP7\|ETFA_PSEAE | Electron transfer flavoprotein subunit alpha OS=Pseudomonas aeruginosa (strain ATCC 15692 / DSM 22644 / CIP 104116 / JCM 14847 / LMG 12228 / 1C / PRS 101 / PAO1) GN=etfA PE=3 SV=1 | 67.55 |
| Q9HZ62\|MUPP_PSEAE | N-acetylmuramic acid 6-phosphate phosphatase OS=Pseudomonas aeruginosa (strain ATCC 15692 / DSM 22644 / CIP 104116 / JCM 14847 / LMG 12228 / 1C / PRS 101 / PAO1) GN=mupP PE=1 SV=1 | 67.51 |
| Q9HWE4\|RS17_PSEAE | 30S ribosomal protein S17 OS=Pseudomonas aeruginosa (strain ATCC 15692 / DSM 22644 / CIP 104116 / JCM 14847 / LMG 12228 / 1C / PRS 101 / PAO1) GN=rpsQ PE=3 SV=1 | 67.45 |
| P20581\|PQSE_PSEAE | 2-aminobenzoylacetyl-CoA thioesterase OS=Pseudomonas aeruginosa (strain ATCC 15692 / DSM 22644 / CIP 104116 / JCM 14847 / LMG 12228 / 1C / PRS 101 / PAO1) GN=pqsE PE=1 SV=1 | 67.14 |
| P15275\|ALGQ_PSEAE | Transcriptional regulatory protein AlgQ OS=Pseudomonas aeruginosa (strain ATCC 15692 / DSM 22644 / CIP 104116 / JCM 14847 / LMG 12228 / 1C / PRS 101 / PAO1) GN=algQ PE=3 SV=1 | 66.47 |
| tr\|Q9HV81\|Q9HV81_PSEAE | Uncharacterized protein OS=Pseudomonas aeruginosa (strain ATCC 15692 / DSM 22644 / CIP 104116 / JCM 14847 / LMG 12228 / 1C / PRS 101 / PAO1) GN=PA4717 PE=4 SV=1 | 66.45 |
| Q9HWP3\|SYY1_PSEAE | Tyrosine--tRNA ligase 1 OS=Pseudomonas aeruginosa (strain ATCC 15692 / DSM 22644 / CIP 104116 / JCM 14847 / LMG 12228 / 1C / PRS 101 / PAO1) GN=tyrS1 PE=3 SV=1 | 65.76 |
| tr\|Q9HUA9\|Q9HUA9_PSEAE | Probable ATP-binding component of ABC transporter OS=Pseudomonas aeruginosa (strain ATCC 15692 / DSM 22644 / CIP 104116 / JCM 14847 / LMG 12228 / 1C / PRS 101 / PAO1) GN=PA5074 PE=4 SV=1 | 65.5 |
| tr\|Q9I1N8\|Q9I1N8_PSEAE | PslA OS=Pseudomonas aeruginosa (strain ATCC 15692 / DSM 22644 / CIP 104116 / JCM 14847 / LMG 12228 / 1C / PRS 101 / PAO1) GN=pslA PE=4 SV=1 | 65.42 |
| tr\|Q9HUG1\|Q9HUG1_PSEAE | Probable glycosyl transferase OS=Pseudomonas aeruginosa (strain ATCC 15692 / DSM 22644 / CIP 104116 / JCM 14847 / LMG 12228 / 1C / PRS 101 / PAO1) GN=PA5004 PE=4 SV=1 | 65.16 |
| tr\|Q9HVR6\|Q9HVR6_PSEAE | Probable ATP-binding component of ABC transporter OS=Pseudomonas aeruginosa (strain ATCC 15692 / DSM 22644 / CIP 104116 / JCM 14847 / LMG 12228 / 1C / PRS 101 / PAO1) GN=PA4505 PE=3 SV=1 | 65.09 |
| tr\|Q9I511\|Q9I511_PSEAE | Uncharacterized protein OS=Pseudomonas aeruginosa (strain ATCC 15692 / DSM 22644 / CIP 104116 / JCM 14847 / LMG 12228 / 1C / PRS 101 / PAO1) GN=PA0947 PE=3 SV=1 | 64.63 |
| Q9HWE3\|RL29_PSEAE | 50S ribosomal protein L29 OS=Pseudomonas aeruginosa (strain ATCC 15692 / DSM 22644 / CIP 104116 / JCM 14847 / LMG 12228 / 1C / PRS 101 / PAO1) GN=rpmC PE=1 SV=1 | 64.58 |
| Q9HWD9\|RS19_PSEAE | 30S ribosomal protein S19 OS=Pseudomonas aeruginosa (strain ATCC 15692 / DSM 22644 / CIP 104116 / JCM 14847 / LMG 12228 / 1C / PRS 101 / PAO1) GN=rpsS PE=3 SV=1 | 63.81 |
| Q9HWC8\|RL7_PSEAE | 50S ribosomal protein L7/L12 OS=Pseudomonas aeruginosa (strain ATCC 15692 / DSM 22644 / CIP 104116 / JCM 14847 / LMG 12228 / 1C / PRS 101 / PAO1) GN=rplL PE=3 SV=1 | 63.71 |
| tr\|Q7DC81\|Q7DC81_PSEAE | Phenazine biosynthesis protein PhzE OS=Pseudomonas aeruginosa (strain ATCC 15692 / DSM 22644 / CIP 104116 / JCM 14847 / LMG 12228 / 1C / PRS 101 / PAO1) GN=phzE1 PE=4 SV=1 | 63.5 |
| tr\|Q9HVI5\|Q9HVI5_PSEAE | Uncharacterized protein OS=Pseudomonas aeruginosa (strain ATCC 15692 / DSM 22644 / CIP 104116 / JCM 14847 / LMG 12228 / 1C / PRS 101 / PAO1) GN=PA4604 PE=4 SV=1 | 63.31 |
| tr\|Q9HVN9\|Q9HVN9_PSEAE | NADH dehydrogenase OS=Pseudomonas aeruginosa (strain ATCC 15692 / DSM 22644 / CIP 104116 / JCM 14847 / LMG 12228 / 1C / PRS 101 / PAO1) GN=ndh PE=4 SV=1 | 63.11 |
| Q51391\|GLPR_PSEAE | Glycerol-3-phosphate regulon repressor OS=Pseudomonas aeruginosa (strain ATCC 15692 / DSM 22644 / CIP 104116 / JCM 14847 / LMG 12228 / 1C / PRS 101 / PAO1) GN=glpR PE=3 SV=2 | 62.03 |
| tr\|Q9HTX0\|Q9HTX0_PSEAE | Uncharacterized protein OS=Pseudomonas aeruginosa (strain ATCC 15692 / DSM 22644 / CIP 104116 / JCM 14847 / LMG 12228 / 1C / PRS 101 / PAO1) GN=PA5220 PE=4 SV=1 | 61.96 |
| Q9I553\|SYA_PSEAE | Alanine--tRNA ligase OS=Pseudomonas aeruginosa (strain ATCC 15692 / DSM 22644 / CIP 104116 / JCM 14847 / LMG 12228 / 1C / PRS 101 / PAO1) GN=alaS PE=3 SV=1 | 61.69 |
| tr\|Q9HVH3\|Q9HVH3_PSEAE | Uncharacterized protein OS=Pseudomonas aeruginosa (strain ATCC 15692 / DSM 22644 / CIP 104116 / JCM 14847 / LMG 12228 / 1C / PRS 101 / PAO1) GN=PA4618 PE=4 SV=1 | 61.61 |
| Q9XCX8\|ERA_PSEAE | GTPase Era OS=Pseudomonas aeruginosa (strain ATCC 15692 / DSM 22644 / CIP 104116 / JCM 14847 / LMG 12228 / 1C / PRS 101 / PAO1) GN=era PE=3 SV=2 | 61.3 |
| tr\|Q9HUD3\|Q9HUD3_PSEAE | Malic enzyme OS=Pseudomonas aeruginosa (strain ATCC 15692 / DSM 22644 / CIP 104116 / JCM 14847 / LMG 12228 / 1C / PRS 101 / PAO1) GN=PA5046 PE=4 SV=1 | 61.28 |
| tr\|Q9I015\|Q9I015_PSEAE | Probable aminotransferase OS=Pseudomonas aeruginosa (strain ATCC 15692 / DSM 22644 / CIP 104116 / JCM 14847 / LMG 12228 / 1C / PRS 101 / PAO1) GN=PA2828 PE=4 SV=1 | 61.12 |
| tr\|Q9I5A0\|Q9I5A0_PSEAE | Probable oxidoreductase OS=Pseudomonas aeruginosa (strain ATCC 15692 / DSM 22644 / CIP 104116 / JCM 14847 / LMG 12228 / 1C / PRS 101 / PAO1) GN=PA0840 PE=4 SV=1 | 60.93 |
| Q9HU65\|GLN1B_PSEAE | Glutamine synthetase OS=Pseudomonas aeruginosa (strain ATCC 15692 / DSM 22644 / CIP 104116 / JCM 14847 / LMG 12228 / 1C / PRS 101 / PAO1) GN=glnA PE=1 SV=1 | 60.9 |
| tr\|Q9HVY4\|Q9HVY4_PSEAE | Ubiquinol-cytochrome c reductase iron-sulfur subunit OS=Pseudomonas aeruginosa (strain ATCC 15692 / DSM 22644 / CIP 104116 / JCM 14847 / LMG 12228 / 1C / PRS 101 / PAO1) GN=PA4431 PE=4 SV=1 | 60.73 |
| tr\|Q9HWK5\|Q9HWK5_PSEAE | Peptidylprolyl isomerase OS=Pseudomonas aeruginosa (strain ATCC 15692 / DSM 22644 / CIP 104116 / JCM 14847 / LMG 12228 / 1C / PRS 101 / PAO1) GN=ppiC2 PE=4 SV=1 | 60.7 |
| O69754\|PHZF_PSEAE | Trans-2 3-dihydro-3-hydroxyanthranilate isomerase OS=Pseudomonas aeruginosa (strain ATCC 15692 / DSM 22644 / CIP 104116 / JCM 14847 / LMG 12228 / 1C / PRS 101 / PAO1) GN=phzF1 PE=3 SV=2 | 60.65 |
| tr\|Q9HZ81\|Q9HZ81_PSEAE | Probable NAD-dependent epimerase/dehydratase WbpK OS=Pseudomonas aeruginosa (strain ATCC 15692 / DSM 22644 / CIP 104116 / JCM 14847 / LMG 12228 / 1C / PRS 101 / PAO1) GN=wbpK PE=4 SV=1 | 60.56 |
| Q51385\|RLMN_PSEAE | Dual-specificity RNA methyltransferase RlmN OS=Pseudomonas aeruginosa (strain ATCC 15692 / DSM 22644 / CIP 104116 / JCM 14847 / LMG 12228 / 1C / PRS 101 / PAO1) GN=rlmN PE=3 SV=2 | 60.04 |
| P33883\|LASI_PSEAE | Acyl-homoserine-lactone synthase OS=Pseudomonas aeruginosa (strain ATCC 15692 / DSM 22644 / CIP 104116 / JCM 14847 / LMG 12228 / 1C / PRS 101 / PAO1) GN=lasI PE=1 SV=1 | 59.7 |
| Q51559\|RHLA_PSEAE | Rhamnosyltransferase 1 subunit A OS=Pseudomonas aeruginosa (strain ATCC 15692 / DSM 22644 / CIP 104116 / JCM 14847 / LMG 12228 / 1C / PRS 101 / PAO1) GN=rhlA PE=2 SV=2 | 59.27 |
| P11221\|OPRI_PSEAE | Major outer membrane lipoprotein OS=Pseudomonas aeruginosa (strain ATCC 15692 / DSM 22644 / CIP 104116 / JCM 14847 / LMG 12228 / 1C / PRS 101 / PAO1) GN=oprI PE=3 SV=1 | 59.23 |
| tr\|Q9I0Z9\|Q9I0Z9_PSEAE | Uncharacterized protein OS=Pseudomonas aeruginosa (strain ATCC 15692 / DSM 22644 / CIP 104116 / JCM 14847 / LMG 12228 / 1C / PRS 101 / PAO1) GN=PA2483 PE=4 SV=1 | 59.18 |
| tr\|Q9HTY1\|Q9HTY1_PSEAE | Uncharacterized protein OS=Pseudomonas aeruginosa (strain ATCC 15692 / DSM 22644 / CIP 104116 / JCM 14847 / LMG 12228 / 1C / PRS 101 / PAO1) GN=PA5209 PE=4 SV=1 | 59.15 |
| tr\|Q9HTW7\|Q9HTW7_PSEAE | UbiH protein OS=Pseudomonas aeruginosa (strain ATCC 15692 / DSM 22644 / CIP 104116 / JCM 14847 / LMG 12228 / 1C / PRS 101 / PAO1) GN=ubiH PE=4 SV=1 | 59.14 |
| Q9HUM6\|PURA_PSEAE | Adenylosuccinate synthetase OS=Pseudomonas aeruginosa (strain ATCC 15692 / DSM 22644 / CIP 104116 / JCM 14847 / LMG 12228 / 1C / PRS 101 / PAO1) GN=purA PE=3 SV=1 | 59.01 |
| tr\|Q9HWI1\|Q9HWI1_PSEAE | Uncharacterized protein OS=Pseudomonas aeruginosa (strain ATCC 15692 / DSM 22644 / CIP 104116 / JCM 14847 / LMG 12228 / 1C / PRS 101 / PAO1) GN=PA4200 PE=4 SV=1 | 58.5 |
| tr\|Q9I0S9\|Q9I0S9_PSEAE | Probable AMP-binding enzyme OS=Pseudomonas aeruginosa (strain ATCC 15692 / DSM 22644 / CIP 104116 / JCM 14847 / LMG 12228 / 1C / PRS 101 / PAO1) GN=PA2555 PE=4 SV=1 | 58.4 |
| Q9X4P2\|RPPH_PSEAE | RNA pyrophosphohydrolase OS=Pseudomonas aeruginosa (strain ATCC 15692 / DSM 22644 / CIP 104116 / JCM 14847 / LMG 12228 / 1C / PRS 101 / PAO1) GN=rppH PE=3 SV=1 | 57.94 |
| tr\|Q9HW68\|Q9HW68_PSEAE | Fumarate hydratase class I OS=Pseudomonas aeruginosa (strain ATCC 15692 / DSM 22644 / CIP 104116 / JCM 14847 / LMG 12228 / 1C / PRS 101 / PAO1) GN=PA4333 PE=3 SV=1 | 57.23 |
| tr\|Q9I5A8\|Q9I5A8_PSEAE | Uncharacterized protein OS=Pseudomonas aeruginosa (strain ATCC 15692 / DSM 22644 / CIP 104116 / JCM 14847 / LMG 12228 / 1C / PRS 101 / PAO1) GN=PA0832 PE=4 SV=1 | 57.19 |
| P25060\|GSPL_PSEAE | Type II secretion system protein L OS=Pseudomonas aeruginosa (strain ATCC 15692 / DSM 22644 / CIP 104116 / JCM 14847 / LMG 12228 / 1C / PRS 101 / PAO1) GN=xcpY PE=3 SV=2 | 57.17 |
| tr\|Q9HXR3\|Q9HXR3_PSEAE | Uncharacterized protein OS=Pseudomonas aeruginosa (strain ATCC 15692 / DSM 22644 / CIP 104116 / JCM 14847 / LMG 12228 / 1C / PRS 101 / PAO1) GN=PA3729 PE=4 SV=1 | 56.81 |
| tr\|Q9I0B4\|Q9I0B4_PSEAE | Uncharacterized protein OS=Pseudomonas aeruginosa (strain ATCC 15692 / DSM 22644 / CIP 104116 / JCM 14847 / LMG 12228 / 1C / PRS 101 / PAO1) GN=PA2728 PE=4 SV=1 | 56.47 |
| tr\|Q9I5R4\|Q9I5R4_PSEAE | Probable ATPase OS=Pseudomonas aeruginosa (strain ATCC 15692 / DSM 22644 / CIP 104116 / JCM 14847 / LMG 12228 / 1C / PRS 101 / PAO1) GN=PA0657 PE=4 SV=1 | 56.13 |
| tr\|Q9I0L4\|Q9I0L4_PSEAE | Isocitrate dehydrogenase OS=Pseudomonas aeruginosa (strain ATCC 15692 / DSM 22644 / CIP 104116 / JCM 14847 / LMG 12228 / 1C / PRS 101 / PAO1) GN=idh PE=4 SV=1 | 56.02 |
| tr\|Q9I5J7\|Q9I5J7_PSEAE | Uncharacterized protein OS=Pseudomonas aeruginosa (strain ATCC 15692 / DSM 22644 / CIP 104116 / JCM 14847 / LMG 12228 / 1C / PRS 101 / PAO1) GN=PA0732 PE=4 SV=1 | 55.76 |
| tr\|Q9HTD9\|Q9HTD9_PSEAE | Alcohol dehydrogenase OS=Pseudomonas aeruginosa (strain ATCC 15692 / DSM 22644 / CIP 104116 / JCM 14847 / LMG 12228 / 1C / PRS 101 / PAO1) GN=adhA PE=1 SV=1 | 55.73 |
| Q9HTJ1\|BETB_PSEAE | NAD/NADP-dependent betaine aldehyde dehydrogenase OS=Pseudomonas aeruginosa (strain ATCC 15692 / DSM 22644 / CIP 104116 / JCM 14847 / LMG 12228 / 1C / PRS 101 / PAO1) GN=betB PE=1 SV=1 | 55.62 |
| tr\|Q9I0A8\|Q9I0A8_PSEAE | Uncharacterized protein OS=Pseudomonas aeruginosa (strain ATCC 15692 / DSM 22644 / CIP 104116 / JCM 14847 / LMG 12228 / 1C / PRS 101 / PAO1) GN=PA2734 PE=4 SV=1 | 55.41 |
| tr\|Q9HV83\|Q9HV83_PSEAE | Probable aminotransferase OS=Pseudomonas aeruginosa (strain ATCC 15692 / DSM 22644 / CIP 104116 / JCM 14847 / LMG 12228 / 1C / PRS 101 / PAO1) GN=PA4715 PE=1 SV=1 | 54.68 |
| tr\|Q9I4W4\|Q9I4W4_PSEAE | Uncharacterized protein OS=Pseudomonas aeruginosa (strain ATCC 15692 / DSM 22644 / CIP 104116 / JCM 14847 / LMG 12228 / 1C / PRS 101 / PAO1) GN=PA1009 PE=4 SV=1 | 54.64 |
| tr\|Q9HU34\|Q9HU34_PSEAE | Probable short-chain dehydrogenase OS=Pseudomonas aeruginosa (strain ATCC 15692 / DSM 22644 / CIP 104116 / JCM 14847 / LMG 12228 / 1C / PRS 101 / PAO1) GN=PA5150 PE=4 SV=1 | 54.58 |
| tr\|G3XCZ4\|G3XCZ4_PSEAE | Uncharacterized protein OS=Pseudomonas aeruginosa (strain ATCC 15692 / DSM 22644 / CIP 104116 / JCM 14847 / LMG 12228 / 1C / PRS 101 / PAO1) GN=PA0891 PE=4 SV=1 | 54.52 |
| tr\|Q9HXU8\|Q9HXU8_PSEAE | Lipotoxon F LptF OS=Pseudomonas aeruginosa (strain ATCC 15692 / DSM 22644 / CIP 104116 / JCM 14847 / LMG 12228 / 1C / PRS 101 / PAO1) GN=lptF PE=3 SV=1 | 54.38 |
| tr\|Q9I494\|Q9I494_PSEAE | Uncharacterized protein OS=Pseudomonas aeruginosa (strain ATCC 15692 / DSM 22644 / CIP 104116 / JCM 14847 / LMG 12228 / 1C / PRS 101 / PAO1) GN=PA1244 PE=1 SV=1 | 54.18 |
| P30720\|CH10_PSEAE | 10 kDa chaperonin OS=Pseudomonas aeruginosa (strain ATCC 15692 / DSM 22644 / CIP 104116 / JCM 14847 / LMG 12228 / 1C / PRS 101 / PAO1) GN=groS PE=3 SV=1 | 53.63 |
| tr\|Q9HVU7\|Q9HVU7_PSEAE | Uncharacterized protein OS=Pseudomonas aeruginosa (strain ATCC 15692 / DSM 22644 / CIP 104116 / JCM 14847 / LMG 12228 / 1C / PRS 101 / PAO1) GN=PA4474 PE=4 SV=1 | 53.56 |
| Q9I138\|GLYA2_PSEAE | Serine hydroxymethyltransferase 2 OS=Pseudomonas aeruginosa (strain ATCC 15692 / DSM 22644 / CIP 104116 / JCM 14847 / LMG 12228 / 1C / PRS 101 / PAO1) GN=glyA2 PE=3 SV=1 | 53.18 |
| tr\|Q9HW98\|Q9HW98_PSEAE | TadA ATPase OS=Pseudomonas aeruginosa (strain ATCC 15692 / DSM 22644 / CIP 104116 / JCM 14847 / LMG 12228 / 1C / PRS 101 / PAO1) GN=tadA PE=4 SV=1 | 53.18 |
| tr\|G3XD20\|G3XD20_PSEAE | Periplasmic serine endoprotease DegP-like OS=Pseudomonas aeruginosa (strain ATCC 15692 / DSM 22644 / CIP 104116 / JCM 14847 / LMG 12228 / 1C / PRS 101 / PAO1) GN=mucD PE=3 SV=1 | 52.71 |
| Q9KGU6\|DXR_PSEAE | 1-deoxy-D-xylulose 5-phosphate reductoisomerase OS=Pseudomonas aeruginosa (strain ATCC 15692 / DSM 22644 / CIP 104116 / JCM 14847 / LMG 12228 / 1C / PRS 101 / PAO1) GN=dxr PE=1 SV=1 | 52.51 |
| tr\|Q9HUM1\|Q9HUM1_PSEAE | GTPase HflX OS=Pseudomonas aeruginosa (strain ATCC 15692 / DSM 22644 / CIP 104116 / JCM 14847 / LMG 12228 / 1C / PRS 101 / PAO1) GN=hflX PE=3 SV=1 | 52.04 |
| Q9HWF0\|RL6_PSEAE | 50S ribosomal protein L6 OS=Pseudomonas aeruginosa (strain ATCC 15692 / DSM 22644 / CIP 104116 / JCM 14847 / LMG 12228 / 1C / PRS 101 / PAO1) GN=rplF PE=3 SV=1 | 51.9 |
| tr\|G3XCW7\|G3XCW7_PSEAE | Probable plasmid partitioning protein OS=Pseudomonas aeruginosa (strain ATCC 15692 / DSM 22644 / CIP 104116 / JCM 14847 / LMG 12228 / 1C / PRS 101 / PAO1) GN=PA1462 PE=4 SV=1 | 51.56 |
| tr\|Q9I693\|Q9I693_PSEAE | Adenosylmethionine-8-amino-7-oxononanoate aminotransferase OS=Pseudomonas aeruginosa (strain ATCC 15692 / DSM 22644 / CIP 104116 / JCM 14847 / LMG 12228 / 1C / PRS 101 / PAO1) GN=bioA PE=3 SV=1 | 51.26 |
| O52760\|RPOA_PSEAE | DNA-directed RNA polymerase subunit alpha OS=Pseudomonas aeruginosa (strain ATCC 15692 / DSM 22644 / CIP 104116 / JCM 14847 / LMG 12228 / 1C / PRS 101 / PAO1) GN=rpoA PE=3 SV=2 | 51.14 |
| tr\|Q9I1H4\|Q9I1H4_PSEAE | Uncharacterized protein OS=Pseudomonas aeruginosa (strain ATCC 15692 / DSM 22644 / CIP 104116 / JCM 14847 / LMG 12228 / 1C / PRS 101 / PAO1) GN=PA2301 PE=4 SV=1 | 50.89 |
| tr\|Q9HTC8\|Q9HTC8_PSEAE | Probable transcriptional regulator OS=Pseudomonas aeruginosa (strain ATCC 15692 / DSM 22644 / CIP 104116 / JCM 14847 / LMG 12228 / 1C / PRS 101 / PAO1) GN=PA5438 PE=4 SV=1 | 50.73 |
| tr\|Q9I0R8\|Q9I0R8_PSEAE | Uncharacterized protein OS=Pseudomonas aeruginosa (strain ATCC 15692 / DSM 22644 / CIP 104116 / JCM 14847 / LMG 12228 / 1C / PRS 101 / PAO1) GN=PA2567 PE=1 SV=1 | 50.42 |
| tr\|Q9I495\|Q9I495_PSEAE | Probable sensor/response regulator hybrid OS=Pseudomonas aeruginosa (strain ATCC 15692 / DSM 22644 / CIP 104116 / JCM 14847 / LMG 12228 / 1C / PRS 101 / PAO1) GN=PA1243 PE=4 SV=1 | 50.2 |
| tr\|Q9HYR6\|Q9HYR6_PSEAE | Uncharacterized protein OS=Pseudomonas aeruginosa (strain ATCC 15692 / DSM 22644 / CIP 104116 / JCM 14847 / LMG 12228 / 1C / PRS 101 / PAO1) GN=PA3329 PE=4 SV=1 | 50.08 |
| tr\|Q9I4Z7\|Q9I4Z7_PSEAE | Probable dna-binding stress protein OS=Pseudomonas aeruginosa (strain ATCC 15692 / DSM 22644 / CIP 104116 / JCM 14847 / LMG 12228 / 1C / PRS 101 / PAO1) GN=PA0962 PE=3 SV=1 | 49.04 |
| Q9HXV3\|CAPP_PSEAE | Phosphoenolpyruvate carboxylase OS=Pseudomonas aeruginosa (strain ATCC 15692 / DSM 22644 / CIP 104116 / JCM 14847 / LMG 12228 / 1C / PRS 101 / PAO1) GN=ppc PE=3 SV=1 | 49 |
| P80358\|ASTG_PSEAE | Arginine N-succinyltransferase subunit beta OS=Pseudomonas aeruginosa (strain ATCC 15692 / DSM 22644 / CIP 104116 / JCM 14847 / LMG 12228 / 1C / PRS 101 / PAO1) GN=aruG PE=1 SV=2 | 48.28 |
| Q51465\|FLIM_PSEAE | Flagellar motor switch protein FliM OS=Pseudomonas aeruginosa (strain ATCC 15692 / DSM 22644 / CIP 104116 / JCM 14847 / LMG 12228 / 1C / PRS 101 / PAO1) GN=fliM PE=3 SV=2 | 48.2 |
| tr\|Q9HYD1\|Q9HYD1_PSEAE | Rhamnosyltransferase chain B OS=Pseudomonas aeruginosa (strain ATCC 15692 / DSM 22644 / CIP 104116 / JCM 14847 / LMG 12228 / 1C / PRS 101 / PAO1) GN=rhlB PE=4 SV=1 | 48.05 |
| Q9HUC8\|SYR_PSEAE | Arginine--tRNA ligase OS=Pseudomonas aeruginosa (strain ATCC 15692 / DSM 22644 / CIP 104116 / JCM 14847 / LMG 12228 / 1C / PRS 101 / PAO1) GN=argS PE=3 SV=1 | 47.7 |
| Q9I502\|SYP_PSEAE | Proline--tRNA ligase OS=Pseudomonas aeruginosa (strain ATCC 15692 / DSM 22644 / CIP 104116 / JCM 14847 / LMG 12228 / 1C / PRS 101 / PAO1) GN=proS PE=1 SV=1 | 47.66 |
| Q9HUB8\|UBIB_PSEAE | Probable protein kinase UbiB OS=Pseudomonas aeruginosa (strain ATCC 15692 / DSM 22644 / CIP 104116 / JCM 14847 / LMG 12228 / 1C / PRS 101 / PAO1) GN=ubiB PE=3 SV=1 | 47.43 |
| Q9I4E6\|TTCA_PSEAE | tRNA-cytidine(32) 2-sulfurtransferase OS=Pseudomonas aeruginosa (strain ATCC 15692 / DSM 22644 / CIP 104116 / JCM 14847 / LMG 12228 / 1C / PRS 101 / PAO1) GN=ttcA PE=3 SV=1 | 47.18 |
| Q9HWD7\|RL23_PSEAE | 50S ribosomal protein L23 OS=Pseudomonas aeruginosa (strain ATCC 15692 / DSM 22644 / CIP 104116 / JCM 14847 / LMG 12228 / 1C / PRS 101 / PAO1) GN=rplW PE=3 SV=1 | 47.1 |
| tr\|Q9I4N9\|Q9I4N9_PSEAE | Uncharacterized protein OS=Pseudomonas aeruginosa (strain ATCC 15692 / DSM 22644 / CIP 104116 / JCM 14847 / LMG 12228 / 1C / PRS 101 / PAO1) GN=PA1090 PE=4 SV=1 | 46.92 |
| tr\|C6JW53\|C6JW53_PSEAE | Uncharacterized protein OS=Pseudomonas aeruginosa (strain ATCC 15692 / DSM 22644 / CIP 104116 / JCM 14847 / LMG 12228 / 1C / PRS 101 / PAO1) GN=PA4673.15 PE=4 SV=1 | 46.83 |
| Q9HWZ6\|IPYR_PSEAE | Inorganic pyrophosphatase OS=Pseudomonas aeruginosa (strain ATCC 15692 / DSM 22644 / CIP 104116 / JCM 14847 / LMG 12228 / 1C / PRS 101 / PAO1) GN=ppa PE=1 SV=1 | 46.83 |
| tr\|Q9I4Q9\|Q9I4Q9_PSEAE | Uncharacterized protein OS=Pseudomonas aeruginosa (strain ATCC 15692 / DSM 22644 / CIP 104116 / JCM 14847 / LMG 12228 / 1C / PRS 101 / PAO1) GN=PA1065 PE=4 SV=1 | 46.82 |
| Q9I0J4\|NUOI_PSEAE | NADH-quinone oxidoreductase subunit I OS=Pseudomonas aeruginosa (strain ATCC 15692 / DSM 22644 / CIP 104116 / JCM 14847 / LMG 12228 / 1C / PRS 101 / PAO1) GN=nuoI PE=3 SV=1 | 46.69 |
| tr\|Q9I2T8\|Q9I2T8_PSEAE | Peptidylprolyl isomerase OS=Pseudomonas aeruginosa (strain ATCC 15692 / DSM 22644 / CIP 104116 / JCM 14847 / LMG 12228 / 1C / PRS 101 / PAO1) GN=ppiD PE=4 SV=1 | 46.34 |
| tr\|Q9HWJ1\|Q9HWJ1_PSEAE | Probable FAD-dependent monooxygenase OS=Pseudomonas aeruginosa (strain ATCC 15692 / DSM 22644 / CIP 104116 / JCM 14847 / LMG 12228 / 1C / PRS 101 / PAO1) GN=pqsL PE=1 SV=1 | 46.19 |
| tr\|Q9HUW7\|Q9HUW7_PSEAE | Probable two-component response regulator OS=Pseudomonas aeruginosa (strain ATCC 15692 / DSM 22644 / CIP 104116 / JCM 14847 / LMG 12228 / 1C / PRS 101 / PAO1) GN=PA4843 PE=4 SV=1 | 46.18 |
| tr\|Q9I107\|Q9I107_PSEAE | Probable cytochrome P450 OS=Pseudomonas aeruginosa (strain ATCC 15692 / DSM 22644 / CIP 104116 / JCM 14847 / LMG 12228 / 1C / PRS 101 / PAO1) GN=PA2475 PE=3 SV=1 | 45.74 |
| Q9I352\|HEMTB_PSEAE | Bacteriohemerythrin OS=Pseudomonas aeruginosa (strain ATCC 15692 / DSM 22644 / CIP 104116 / JCM 14847 / LMG 12228 / 1C / PRS 101 / PAO1) GN=PA1673 PE=3 SV=1 | 45.67 |
| Q9I347\|PRMB_PSEAE | 50S ribosomal protein L3 glutamine methyltransferase OS=Pseudomonas aeruginosa (strain ATCC 15692 / DSM 22644 / CIP 104116 / JCM 14847 / LMG 12228 / 1C / PRS 101 / PAO1) GN=prmB PE=3 SV=1 | 45.12 |
| tr\|Q9HVA1\|Q9HVA1_PSEAE | Acetolactate synthase isozyme III small subunit OS=Pseudomonas aeruginosa (strain ATCC 15692 / DSM 22644 / CIP 104116 / JCM 14847 / LMG 12228 / 1C / PRS 101 / PAO1) GN=ilvH PE=4 SV=1 | 45.06 |
| P43334\|PH4H_PSEAE | Phenylalanine-4-hydroxylase OS=Pseudomonas aeruginosa (strain ATCC 15692 / DSM 22644 / CIP 104116 / JCM 14847 / LMG 12228 / 1C / PRS 101 / PAO1) GN=phhA PE=3 SV=2 | 44.79 |
| Q9HT80\|DPO1_PSEAE | DNA polymerase I OS=Pseudomonas aeruginosa (strain ATCC 15692 / DSM 22644 / CIP 104116 / JCM 14847 / LMG 12228 / 1C / PRS 101 / PAO1) GN=polA PE=3 SV=1 | 44.24 |
| tr\|Q9HZI5\|Q9HZI5_PSEAE | Uncharacterized protein OS=Pseudomonas aeruginosa (strain ATCC 15692 / DSM 22644 / CIP 104116 / JCM 14847 / LMG 12228 / 1C / PRS 101 / PAO1) GN=PA3021 PE=4 SV=1 | 44.09 |
| Q9HT06\|YIDC_PSEAE | Membrane protein insertase YidC OS=Pseudomonas aeruginosa (strain ATCC 15692 / DSM 22644 / CIP 104116 / JCM 14847 / LMG 12228 / 1C / PRS 101 / PAO1) GN=yidC PE=3 SV=1 | 43.77 |
| Q9I088\|ECOT_PSEAE | Ecotin OS=Pseudomonas aeruginosa (strain ATCC 15692 / DSM 22644 / CIP 104116 / JCM 14847 / LMG 12228 / 1C / PRS 101 / PAO1) GN=eco PE=3 SV=1 | 43.66 |
| tr\|Q9HYY4\|Q9HYY4_PSEAE | Probable oxidoreductase OS=Pseudomonas aeruginosa (strain ATCC 15692 / DSM 22644 / CIP 104116 / JCM 14847 / LMG 12228 / 1C / PRS 101 / PAO1) GN=PA3256 PE=4 SV=1 | 43.59 |
| tr\|Q9I622\|Q9I622_PSEAE | Uncharacterized protein OS=Pseudomonas aeruginosa (strain ATCC 15692 / DSM 22644 / CIP 104116 / JCM 14847 / LMG 12228 / 1C / PRS 101 / PAO1) GN=PA0496 PE=4 SV=1 | 43.56 |
| Q9HXE4\|DAUB_PSEAE | NAD(P)H-dependent anabolic L-arginine dehydrogenase DauB OS=Pseudomonas aeruginosa (strain ATCC 15692 / DSM 22644 / CIP 104116 / JCM 14847 / LMG 12228 / 1C / PRS 101 / PAO1) GN=dauB PE=1 SV=1 | 43.52 |
| tr\|Q9I584\|Q9I584_PSEAE | Morphogene protein BolA OS=Pseudomonas aeruginosa (strain ATCC 15692 / DSM 22644 / CIP 104116 / JCM 14847 / LMG 12228 / 1C / PRS 101 / PAO1) GN=bolA PE=3 SV=1 | 43.48 |
| tr\|Q9HTR3\|Q9HTR3_PSEAE | Probable choline transporter OS=Pseudomonas aeruginosa (strain ATCC 15692 / DSM 22644 / CIP 104116 / JCM 14847 / LMG 12228 / 1C / PRS 101 / PAO1) GN=PA5291 PE=3 SV=1 | 43.42 |
| Q9I6Y4\|ADE_PSEAE | Adenine deaminase OS=Pseudomonas aeruginosa (strain ATCC 15692 / DSM 22644 / CIP 104116 / JCM 14847 / LMG 12228 / 1C / PRS 101 / PAO1) GN=PA0148 PE=1 SV=1 | 43.16 |
| Q9HT16\|ATPF_PSEAE | ATP synthase subunit b OS=Pseudomonas aeruginosa (strain ATCC 15692 / DSM 22644 / CIP 104116 / JCM 14847 / LMG 12228 / 1C / PRS 101 / PAO1) GN=atpF PE=3 SV=1 | 43.12 |
| tr\|Q9HW72\|Q9HW72_PSEAE | Pyruvate kinase OS=Pseudomonas aeruginosa (strain ATCC 15692 / DSM 22644 / CIP 104116 / JCM 14847 / LMG 12228 / 1C / PRS 101 / PAO1) GN=pykA PE=3 SV=1 | 42.3 |
| tr\|Q9I1N7\|Q9I1N7_PSEAE | PslB OS=Pseudomonas aeruginosa (strain ATCC 15692 / DSM 22644 / CIP 104116 / JCM 14847 / LMG 12228 / 1C / PRS 101 / PAO1) GN=pslB PE=3 SV=1 | 42.08 |
| tr\|Q9I1B2\|Q9I1B2_PSEAE | Uncharacterized protein OS=Pseudomonas aeruginosa (strain ATCC 15692 / DSM 22644 / CIP 104116 / JCM 14847 / LMG 12228 / 1C / PRS 101 / PAO1) GN=PA2367 PE=4 SV=1 | 41.84 |
| Q9I7C3\|RECF_PSEAE | DNA replication and repair protein RecF OS=Pseudomonas aeruginosa (strain ATCC 15692 / DSM 22644 / CIP 104116 / JCM 14847 / LMG 12228 / 1C / PRS 101 / PAO1) GN=recF PE=3 SV=1 | 41.76 |
| tr\|Q9I4P0\|Q9I4P0_PSEAE | Uncharacterized protein OS=Pseudomonas aeruginosa (strain ATCC 15692 / DSM 22644 / CIP 104116 / JCM 14847 / LMG 12228 / 1C / PRS 101 / PAO1) GN=PA1089 PE=4 SV=1 | 41.68 |
| tr\|Q9HYU4\|Q9HYU4_PSEAE | Long-chain-fatty-acid--CoA ligase OS=Pseudomonas aeruginosa (strain ATCC 15692 / DSM 22644 / CIP 104116 / JCM 14847 / LMG 12228 / 1C / PRS 101 / PAO1) GN=fadD1 PE=4 SV=1 | 41.19 |
| tr\|Q9HYP9\|Q9HYP9_PSEAE | Probable two-component response regulator OS=Pseudomonas aeruginosa (strain ATCC 15692 / DSM 22644 / CIP 104116 / JCM 14847 / LMG 12228 / 1C / PRS 101 / PAO1) GN=PA3346 PE=4 SV=1 | 41.11 |
| P43501\|PILH_PSEAE | Protein PilH OS=Pseudomonas aeruginosa (strain ATCC 15692 / DSM 22644 / CIP 104116 / JCM 14847 / LMG 12228 / 1C / PRS 101 / PAO1) GN=pilH PE=3 SV=1 | 41.02 |
| tr\|Q9HTQ4\|Q9HTQ4_PSEAE | Cytochrome c5 OS=Pseudomonas aeruginosa (strain ATCC 15692 / DSM 22644 / CIP 104116 / JCM 14847 / LMG 12228 / 1C / PRS 101 / PAO1) GN=cycB PE=4 SV=1 | 40.86 |
| tr\|G3XD97\|G3XD97_PSEAE | Transcriptional regulator PtxS OS=Pseudomonas aeruginosa (strain ATCC 15692 / DSM 22644 / CIP 104116 / JCM 14847 / LMG 12228 / 1C / PRS 101 / PAO1) GN=ptxS PE=4 SV=1 | 40.83 |
| tr\|Q9I593\|Q9I593_PSEAE | Probable alkyl hydroperoxide reductase OS=Pseudomonas aeruginosa (strain ATCC 15692 / DSM 22644 / CIP 104116 / JCM 14847 / LMG 12228 / 1C / PRS 101 / PAO1) GN=PA0848 PE=4 SV=1 | 40.65 |
| tr\|Q9I5L2\|Q9I5L2_PSEAE | Uncharacterized protein OS=Pseudomonas aeruginosa (strain ATCC 15692 / DSM 22644 / CIP 104116 / JCM 14847 / LMG 12228 / 1C / PRS 101 / PAO1) GN=PA0716 PE=4 SV=1 | 40.58 |
| tr\|Q9HU24\|Q9HU24_PSEAE | dTDP-glucose 4 6-dehydratase OS=Pseudomonas aeruginosa (strain ATCC 15692 / DSM 22644 / CIP 104116 / JCM 14847 / LMG 12228 / 1C / PRS 101 / PAO1) GN=rmlB PE=3 SV=1 | 40.36 |
| Q9I4X1\|PQSC_PSEAE | 2-heptyl-4(1H)-quinolone synthase subunit PqsC OS=Pseudomonas aeruginosa (strain ATCC 15692 / DSM 22644 / CIP 104116 / JCM 14847 / LMG 12228 / 1C / PRS 101 / PAO1) GN=pqsC PE=1 SV=1 | 39.64 |
| Q9HZQ2\|COBW_PSEAE | Protein CobW OS=Pseudomonas aeruginosa (strain ATCC 15692 / DSM 22644 / CIP 104116 / JCM 14847 / LMG 12228 / 1C / PRS 101 / PAO1) GN=cobW PE=3 SV=1 | 39.38 |
| Q9HWE9\|RS8_PSEAE | 30S ribosomal protein S8 OS=Pseudomonas aeruginosa (strain ATCC 15692 / DSM 22644 / CIP 104116 / JCM 14847 / LMG 12228 / 1C / PRS 101 / PAO1) GN=rpsH PE=3 SV=1 | 39.24 |
| tr\|Q9HVF2\|Q9HVF2_PSEAE | Uncharacterized protein OS=Pseudomonas aeruginosa (strain ATCC 15692 / DSM 22644 / CIP 104116 / JCM 14847 / LMG 12228 / 1C / PRS 101 / PAO1) GN=PA4639 PE=4 SV=1 | 39.18 |
| tr\|Q9I508\|Q9I508_PSEAE | Arsenate reductase OS=Pseudomonas aeruginosa (strain ATCC 15692 / DSM 22644 / CIP 104116 / JCM 14847 / LMG 12228 / 1C / PRS 101 / PAO1) GN=PA0950 PE=3 SV=1 | 38.7 |
| Q9HYC9\|DCD_PSEAE | dCTP deaminase OS=Pseudomonas aeruginosa (strain ATCC 15692 / DSM 22644 / CIP 104116 / JCM 14847 / LMG 12228 / 1C / PRS 101 / PAO1) GN=dcd PE=3 SV=1 | 38.24 |
| tr\|Q9HUD4\|Q9HUD4_PSEAE | Uncharacterized protein OS=Pseudomonas aeruginosa (strain ATCC 15692 / DSM 22644 / CIP 104116 / JCM 14847 / LMG 12228 / 1C / PRS 101 / PAO1) GN=PA5037 PE=4 SV=1 | 37.9 |
| tr\|Q9HWA4\|Q9HWA4_PSEAE | Two-component response regulator PprB OS=Pseudomonas aeruginosa (strain ATCC 15692 / DSM 22644 / CIP 104116 / JCM 14847 / LMG 12228 / 1C / PRS 101 / PAO1) GN=pprB PE=4 SV=1 | 37.59 |
| tr\|Q9HTY2\|Q9HTY2_PSEAE | Uncharacterized protein OS=Pseudomonas aeruginosa (strain ATCC 15692 / DSM 22644 / CIP 104116 / JCM 14847 / LMG 12228 / 1C / PRS 101 / PAO1) GN=PA5208 PE=4 SV=1 | 36.8 |
| Q9HU56\|SECB_PSEAE | Protein-export protein SecB OS=Pseudomonas aeruginosa (strain ATCC 15692 / DSM 22644 / CIP 104116 / JCM 14847 / LMG 12228 / 1C / PRS 101 / PAO1) GN=secB PE=3 SV=1 | 36.75 |
| tr\|G3XD85\|G3XD85_PSEAE | Probable glycosyltransferase WbpH OS=Pseudomonas aeruginosa (strain ATCC 15692 / DSM 22644 / CIP 104116 / JCM 14847 / LMG 12228 / 1C / PRS 101 / PAO1) GN=wbpH PE=4 SV=1 | 36.67 |
| tr\|Q9I3G8\|Q9I3G8_PSEAE | Probable cation-transporting P-type ATPase OS=Pseudomonas aeruginosa (strain ATCC 15692 / DSM 22644 / CIP 104116 / JCM 14847 / LMG 12228 / 1C / PRS 101 / PAO1) GN=PA1549 PE=3 SV=1 | 36.48 |
| Q9I0M6\|SYS_PSEAE | Serine--tRNA ligase OS=Pseudomonas aeruginosa (strain ATCC 15692 / DSM 22644 / CIP 104116 / JCM 14847 / LMG 12228 / 1C / PRS 101 / PAO1) GN=serS PE=3 SV=1 | 36.37 |
| tr\|Q9I4W2\|Q9I4W2_PSEAE | Uncharacterized protein OS=Pseudomonas aeruginosa (strain ATCC 15692 / DSM 22644 / CIP 104116 / JCM 14847 / LMG 12228 / 1C / PRS 101 / PAO1) GN=PA1011 PE=4 SV=1 | 33.68 |
| tr\|Q9HY55\|Q9HY55_PSEAE | Phosphotransferase system transporter enzyme I FruI OS=Pseudomonas aeruginosa (strain ATCC 15692 / DSM 22644 / CIP 104116 / JCM 14847 / LMG 12228 / 1C / PRS 101 / PAO1) GN=fruI PE=3 SV=1 | 33.47 |
| tr\|Q9HW12\|Q9HW12_PSEAE | Uncharacterized protein OS=Pseudomonas aeruginosa (strain ATCC 15692 / DSM 22644 / CIP 104116 / JCM 14847 / LMG 12228 / 1C / PRS 101 / PAO1) GN=PA4394 PE=4 SV=1 | 33.24 |
| tr\|Q9I4X0\|Q9I4X0_PSEAE | Transcriptional regulator MvfR OS=Pseudomonas aeruginosa (strain ATCC 15692 / DSM 22644 / CIP 104116 / JCM 14847 / LMG 12228 / 1C / PRS 101 / PAO1) GN=mvfR PE=4 SV=1 | 32.99 |
| O82851\|EFTS_PSEAE | Elongation factor Ts OS=Pseudomonas aeruginosa (strain ATCC 15692 / DSM 22644 / CIP 104116 / JCM 14847 / LMG 12228 / 1C / PRS 101 / PAO1) GN=tsf PE=3 SV=1 | 32.81 |
| tr\|Q9I5T8\|Q9I5T8_PSEAE | Probable two-component sensor OS=Pseudomonas aeruginosa (strain ATCC 15692 / DSM 22644 / CIP 104116 / JCM 14847 / LMG 12228 / 1C / PRS 101 / PAO1) GN=PA0600 PE=4 SV=1 | 32.79 |
| tr\|Q9HZ12\|Q9HZ12_PSEAE | Probable ATP-binding/permease fusion ABC transporter OS=Pseudomonas aeruginosa (strain ATCC 15692 / DSM 22644 / CIP 104116 / JCM 14847 / LMG 12228 / 1C / PRS 101 / PAO1) GN=PA3228 PE=4 SV=1 | 32.43 |
| tr\|Q9I5Q7\|Q9I5Q7_PSEAE | Uncharacterized protein OS=Pseudomonas aeruginosa (strain ATCC 15692 / DSM 22644 / CIP 104116 / JCM 14847 / LMG 12228 / 1C / PRS 101 / PAO1) GN=PA0664 PE=4 SV=1 | 30.53 |
| Q9I4G3\|NAPA_PSEAE | Periplasmic nitrate reductase OS=Pseudomonas aeruginosa (strain ATCC 15692 / DSM 22644 / CIP 104116 / JCM 14847 / LMG 12228 / 1C / PRS 101 / PAO1) GN=napA PE=3 SV=1 | 30.51 |
| Q9HTQ6\|XPT_PSEAE | Xanthine phosphoribosyltransferase OS=Pseudomonas aeruginosa (strain ATCC 15692 / DSM 22644 / CIP 104116 / JCM 14847 / LMG 12228 / 1C / PRS 101 / PAO1) GN=xpt PE=3 SV=1 | 30.31 |
| Q9HUA4\|DTD_PSEAE | D-aminoacyl-tRNA deacylase OS=Pseudomonas aeruginosa (strain ATCC 15692 / DSM 22644 / CIP 104116 / JCM 14847 / LMG 12228 / 1C / PRS 101 / PAO1) GN=dtd PE=3 SV=1 | 30 |
| Q9Z9H0\|GLND_PSEAE | Bifunctional uridylyltransferase/uridylyl-removing enzyme OS=Pseudomonas aeruginosa (strain ATCC 15692 / DSM 22644 / CIP 104116 / JCM 14847 / LMG 12228 / 1C / PRS 101 / PAO1) GN=glnD PE=3 SV=2 | 29.87 |
| tr\|Q9I1A8\|Q9I1A8_PSEAE | Probable ClpA/B-type protease OS=Pseudomonas aeruginosa (strain ATCC 15692 / DSM 22644 / CIP 104116 / JCM 14847 / LMG 12228 / 1C / PRS 101 / PAO1) GN=PA2371 PE=4 SV=1 | 29.64 |
| Q9I1M1\|ODBB_PSEAE | 2-oxoisovalerate dehydrogenase subunit beta OS=Pseudomonas aeruginosa (strain ATCC 15692 / DSM 22644 / CIP 104116 / JCM 14847 / LMG 12228 / 1C / PRS 101 / PAO1) GN=bkdA2 PE=3 SV=1 | 29.41 |
| Q59643\|HEM2_PSEAE | Delta-aminolevulinic acid dehydratase OS=Pseudomonas aeruginosa (strain ATCC 15692 / DSM 22644 / CIP 104116 / JCM 14847 / LMG 12228 / 1C / PRS 101 / PAO1) GN=hemB PE=1 SV=1 | 29.26 |
| tr\|Q9I065\|Q9I065_PSEAE | Uncharacterized protein OS=Pseudomonas aeruginosa (strain ATCC 15692 / DSM 22644 / CIP 104116 / JCM 14847 / LMG 12228 / 1C / PRS 101 / PAO1) GN=PA2778 PE=4 SV=1 | 28.12 |
| tr\|Q9HZ80\|Q9HZ80_PSEAE | Probable glycosyl transferase WbpJ OS=Pseudomonas aeruginosa (strain ATCC 15692 / DSM 22644 / CIP 104116 / JCM 14847 / LMG 12228 / 1C / PRS 101 / PAO1) GN=wbpJ PE=4 SV=1 | 27.78 |
| tr\|Q9I645\|Q9I645_PSEAE | Probable glutathione S-transferase OS=Pseudomonas aeruginosa (strain ATCC 15692 / DSM 22644 / CIP 104116 / JCM 14847 / LMG 12228 / 1C / PRS 101 / PAO1) GN=PA0473 PE=4 SV=1 | 27.61 |
| tr\|Q9HU22\|Q9HU22_PSEAE | Glucose-1-phosphate thymidylyltransferase OS=Pseudomonas aeruginosa (strain ATCC 15692 / DSM 22644 / CIP 104116 / JCM 14847 / LMG 12228 / 1C / PRS 101 / PAO1) GN=rmlA PE=1 SV=1 | 27.55 |
| tr\|Q9HXV0\|Q9HXV0_PSEAE | Probable metal-transporting P-type ATPase OS=Pseudomonas aeruginosa (strain ATCC 15692 / DSM 22644 / CIP 104116 / JCM 14847 / LMG 12228 / 1C / PRS 101 / PAO1) GN=PA3690 PE=3 SV=1 | 25.89 |
| tr\|Q9HVW1\|Q9HVW1_PSEAE | Probable ATP-binding component of ABC transporter OS=Pseudomonas aeruginosa (strain ATCC 15692 / DSM 22644 / CIP 104116 / JCM 14847 / LMG 12228 / 1C / PRS 101 / PAO1) GN=PA4456 PE=4 SV=1 | 24.64 |
| tr\|Q9HX92\|Q9HX92_PSEAE | Probable transcriptional regulator OS=Pseudomonas aeruginosa (strain ATCC 15692 / DSM 22644 / CIP 104116 / JCM 14847 / LMG 12228 / 1C / PRS 101 / PAO1) GN=PA3921 PE=4 SV=1 | 24.26 |
| tr\|Q9I0Y7\|Q9I0Y7_PSEAE | Multidrug efflux outer membrane protein OprN OS=Pseudomonas aeruginosa (strain ATCC 15692 / DSM 22644 / CIP 104116 / JCM 14847 / LMG 12228 / 1C / PRS 101 / PAO1) GN=oprN PE=1 SV=1 | 23.67 |
| Q51397\|OPRJ_PSEAE | Outer membrane protein OprJ OS=Pseudomonas aeruginosa (strain ATCC 15692 / DSM 22644 / CIP 104116 / JCM 14847 / LMG 12228 / 1C / PRS 101 / PAO1) GN=oprJ PE=1 SV=2 | 23.67 |
| Q51487\|OPRM_PSEAE | Outer membrane protein OprM OS=Pseudomonas aeruginosa (strain ATCC 15692 / DSM 22644 / CIP 104116 / JCM 14847 / LMG 12228 / 1C / PRS 101 / PAO1) GN=oprM PE=1 SV=2 | 23.67 |
| tr\|Q9HWH3\|Q9HWH3_PSEAE | Probable outer membrane protein OS=Pseudomonas aeruginosa (strain ATCC 15692 / DSM 22644 / CIP 104116 / JCM 14847 / LMG 12228 / 1C / PRS 101 / PAO1) GN=opmD PE=3 SV=1 | 23.67 |
| tr\|Q9HY88\|Q9HY88_PSEAE | Probable outer membrane protein OS=Pseudomonas aeruginosa (strain ATCC 15692 / DSM 22644 / CIP 104116 / JCM 14847 / LMG 12228 / 1C / PRS 101 / PAO1) GN=PA3521 PE=3 SV=1 | 23.67 |
| tr\|Q9I2F3\|Q9I2F3_PSEAE | Uncharacterized protein OS=Pseudomonas aeruginosa (strain ATCC 15692 / DSM 22644 / CIP 104116 / JCM 14847 / LMG 12228 / 1C / PRS 101 / PAO1) GN=PA1951 PE=4 SV=1 | 23.66 |
| Q9HXI8\|ISCS_PSEAE | Cysteine desulfurase IscS OS=Pseudomonas aeruginosa (strain ATCC 15692 / DSM 22644 / CIP 104116 / JCM 14847 / LMG 12228 / 1C / PRS 101 / PAO1) GN=iscS PE=3 SV=1 | 23.45 |
| tr\|Q9HZY2\|Q9HZY2_PSEAE | Uncharacterized protein OS=Pseudomonas aeruginosa (strain ATCC 15692 / DSM 22644 / CIP 104116 / JCM 14847 / LMG 12228 / 1C / PRS 101 / PAO1) GN=PA2864 PE=4 SV=1 | 23.3 |
| Q51576\|Y3106_PSEAE | Uncharacterized oxidoreductase PA3106 OS=Pseudomonas aeruginosa (strain ATCC 15692 / DSM 22644 / CIP 104116 / JCM 14847 / LMG 12228 / 1C / PRS 101 / PAO1) GN=PA3106 PE=3 SV=1 | 23.18 |
| tr\|Q9I3G0\|Q9I3G0_PSEAE | Cytochrome c oxidase cbb3-type CcoN subunit OS=Pseudomonas aeruginosa (strain ATCC 15692 / DSM 22644 / CIP 104116 / JCM 14847 / LMG 12228 / 1C / PRS 101 / PAO1) GN=ccoN2 PE=3 SV=1 | 22.68 |
| tr\|Q9I3G3\|Q9I3G3_PSEAE | Cytochrome c oxidase cbb3-type CcoN subunit OS=Pseudomonas aeruginosa (strain ATCC 15692 / DSM 22644 / CIP 104116 / JCM 14847 / LMG 12228 / 1C / PRS 101 / PAO1) GN=ccoN1 PE=3 SV=1 | 22.68 |
| Q9HTN2\|ARGB_PSEAE | Acetylglutamate kinase OS=Pseudomonas aeruginosa (strain ATCC 15692 / DSM 22644 / CIP 104116 / JCM 14847 / LMG 12228 / 1C / PRS 101 / PAO1) GN=argB PE=1 SV=3 | 22.2 |
| tr\|G3XCT5\|G3XCT5_PSEAE | Methyltransferase PilK OS=Pseudomonas aeruginosa (strain ATCC 15692 / DSM 22644 / CIP 104116 / JCM 14847 / LMG 12228 / 1C / PRS 101 / PAO1) GN=pilK PE=4 SV=1 | 21.1 |
| Q9HXE2\|DAUR_PSEAE | Transcriptional regulator DauR OS=Pseudomonas aeruginosa (strain ATCC 15692 / DSM 22644 / CIP 104116 / JCM 14847 / LMG 12228 / 1C / PRS 101 / PAO1) GN=dauR PE=2 SV=1 | 21.01 |
| tr\|Q9I2K5\|Q9I2K5_PSEAE | Uncharacterized protein OS=Pseudomonas aeruginosa (strain ATCC 15692 / DSM 22644 / CIP 104116 / JCM 14847 / LMG 12228 / 1C / PRS 101 / PAO1) GN=PA1892 PE=4 SV=1 | 20.43 |
| tr\|Q9HYP5\|Q9HYP5_PSEAE | FlgM OS=Pseudomonas aeruginosa (strain ATCC 15692 / DSM 22644 / CIP 104116 / JCM 14847 / LMG 12228 / 1C / PRS 101 / PAO1) GN=flgM PE=4 SV=1 | 20.13 |

Table S4 Differential expressed gene in PAO1(*proE*) vs PAO1

| Gene | log2FoldChange | padj |
| --- | --- | --- |
| *proE* | 7.11 | 1.89E-181 |
| *PA0848* | 2.93 | 1.72E-10 |
| *PA2294* | 2.83 | 1.79E-14 |
| *betB* | 2.77 | 2.94E-51 |
| *katB* | 2.54 | 1.45E-07 |
| *cobI* | 2.50 | 2.86E-21 |
| *PA1410* | 2.40 | 4.94E-22 |
| *aphA* | 2.39 | 6.68E-17 |
| *opdH* | 2.34 | 1.71E-10 |
| *glcF* | 2.19 | 0.00653986 |
| *betI* | 2.15 | 1.52E-22 |
| *PA5352* | 2.11 | 0.003717328 |
| *trxB2* | 1.99 | 1.00E-05 |
| *PA3287* | 1.96 | 1.22E-05 |
| *PA1699* | 1.96 | 8.46E-08 |
| *PA0752* | 1.93 | 5.90E-13 |
| *PA0754* | 1.91 | 6.40E-13 |
| *PA1701* | 1.90 | 0.000840281 |
| *katE* | 1.78 | 8.36E-12 |
| *PA2163* | 1.76 | 4.65E-15 |
| *PA2162* | 1.75 | 3.66E-14 |
| *betA* | 1.73 | 2.15E-33 |
| *piv* | 1.72 | 8.58E-14 |
| *pscK* | 1.71 | 0.00094959 |
| *PA3933* | 1.67 | 1.79E-14 |
| *glcE* | 1.66 | 0.022970811 |
| *phzG2* | 1.61 | 6.73E-05 |
| *PA0909* | 1.59 | 0.00010589 |
| *pcrG* | 1.57 | 0.00032654 |
| *PA2143* | 1.53 | 2.54E-21 |
| *PA0028* | 1.51 | 6.80E-11 |
| *PA4498* | 1.51 | 3.34E-14 |
| *PA2146* | 1.48 | 0.003166813 |
| *PA1961* | 1.45 | 3.21E-11 |
| *PA4500* | 1.42 | 3.85E-09 |
| *PA2161* | 1.41 | 2.65E-08 |
| *PA2164* | 1.40 | 2.31E-10 |
| *PA2154* | 1.40 | 1.05E-10 |
| *PA2141* | 1.39 | 2.40E-07 |
| *PA0645* | 1.39 | 0.000116876 |
| *PA0027* | 1.37 | 1.97E-14 |
| *PA2139* | 1.37 | 0.001288762 |
| *PA0751* | 1.37 | 6.50E-08 |
| *PA1711* | 1.36 | 1.94E-09 |
| *popB* | 1.34 | 2.79E-06 |
| *PA2170* | 1.33 | 4.50E-05 |
| *PA0639* | 1.33 | 1.90E-08 |
| *PA2159* | 1.33 | 2.23E-10 |
| *PA2165* | 1.31 | 8.04E-09 |
| *PA2166* | 1.30 | 1.36E-08 |
| *PA2155* | 1.30 | 9.29E-08 |
| *plcB* | 1.30 | 1.71E-10 |
| *PA2172* | 1.29 | 1.54E-09 |
| *pscP* | 1.29 | 2.98E-06 |
| *glgB* | 1.28 | 9.81E-08 |
| *PA2184* | 1.28 | 2.68E-08 |
| *PA0912* | 1.26 | 0.001494821 |
| *phzA2* | 1.26 | 0.015971966 |
| *PA2148* | 1.26 | 3.82E-10 |
| *exsC* | 1.25 | 1.20E-09 |
| *PA4172* | 1.24 | 2.46E-07 |
| *PA3288* | 1.24 | 2.46E-07 |
| *popD* | 1.23 | 1.80E-06 |
| *exoY* | 1.23 | 1.04E-07 |
| *PA2135* | 1.22 | 6.23E-08 |
| *PA2156* | 1.22 | 0.000703885 |
| *PA2157* | 1.22 | 2.46E-07 |
| *PA2160* | 1.22 | 1.19E-06 |
| *moeA1* | 1.21 | 0.026189203 |
| *phzB2* | 1.21 | 0.019016194 |
| *PA3278* | 1.21 | 4.19E-05 |
| *pscD* | 1.20 | 7.97E-06 |
| *PA2150* | 1.20 | 1.45E-08 |
| *PA2145* | 1.20 | 1.36E-09 |
| *PA1662* | 1.19 | 2.01E-06 |
| *PA2180* | 1.19 | 7.12E-07 |
| *cupA4* | 1.19 | 0.002112776 |
| *pppA* | 1.18 | 0.000153785 |
| *glgP* | 1.18 | 1.59E-09 |
| *PA0641* | 1.18 | 1.44E-06 |
| *PA1509* | 1.17 | 2.19E-06 |
| *narI* | 1.16 | 0.006204001 |
| *phzG1* | 1.16 | 0.042000796 |
| *exsB* | 1.16 | 0.000164031 |
| *PA0753* | 1.15 | 0.011012269 |
| *PA1697* | 1.15 | 0.000682703 |
| *PA2152* | 1.14 | 2.93E-06 |
| *PA0635* | 1.14 | 2.85E-06 |
| *PA3911* | 1.14 | 4.43E-05 |
| *narG* | 1.14 | 0.00024721 |
| *katN* | 1.13 | 3.47E-05 |
| *pscQ* | 1.13 | 0.000670899 |
| *PA2158* | 1.11 | 3.78E-06 |
| *PA2171* | 1.11 | 8.72E-06 |
| *PA0089* | 1.11 | 0.000682703 |
| *PA1663* | 1.11 | 1.49E-05 |
| *PA0029* | 1.11 | 1.90E-08 |
| *PA2190* | 1.11 | 3.81E-06 |
| *PA0638* | 1.11 | 7.26E-07 |
| *cobQ* | 1.09 | 1.63E-07 |
| *PA2179* | 1.09 | 6.09E-05 |
| *PA1914* | 1.09 | 7.89E-08 |
| *PA1700* | 1.09 | 0.033598392 |
| *PA2173* | 1.09 | 4.62E-05 |
| *PA0647* | 1.09 | 6.68E-05 |
| *PA0636* | 1.07 | 3.79E-05 |
| *PA3913* | 1.05 | 0.001656504 |
| *gabP* | 1.05 | 0.000147228 |
| *aruC* | 1.05 | 4.34E-05 |
| *PA0640* | 1.05 | 2.00E-05 |
| *PA0088* | 1.04 | 3.31E-05 |
| *PA1869* | 1.04 | 2.67E-06 |
| *ppkA* | 1.04 | 1.61E-05 |
| *pcoB* | 1.04 | 0.0003277 |
| *cobD* | 1.04 | 0.000528377 |
| *PA0078* | 1.03 | 1.08E-05 |
| *PA1510* | 1.03 | 5.32E-07 |
| *PA0130* | 1.02 | 0.003045422 |
| *clpV1* | 1.02 | 0.000941849 |
| *PA4171* | 1.01 | 1.24E-06 |
| *PA2134* | 1.01 | 0.001835748 |
| *aruD* | 1.01 | 0.002882837 |
| *PA2151* | 1.00 | 3.55E-07 |
| *PA4652* | 1.00 | 3.19E-11 |
| *PA3971* | -1.01 | 0.001323711 |
| *PA4181* | -1.01 | 6.12E-05 |
| *bdlA* | -1.03 | 4.10E-06 |
| *PA4471* | -1.03 | 0.006985852 |
| *dctA* | -1.03 | 1.17E-05 |
| *PA0149* | -1.04 | 0.006263981 |
| *PA5030* | -1.05 | 0.002627458 |
| *PA4929* | -1.05 | 3.91E-06 |
| *PA4090* | -1.06 | 0.005914684 |
| *PA1283* | -1.08 | 4.26E-05 |
| *PA2286* | -1.08 | 1.79E-14 |
| *PA1187* | -1.09 | 0.000954003 |
| *glpD* | -1.10 | 0.021669983 |
| *hscB* | -1.11 | 2.68E-05 |
| *PA1736* | -1.11 | 5.20E-08 |
| *PA4624* | -1.11 | 3.96E-08 |
| *PA2027* | -1.11 | 0.045391707 |
| *PA0883* | -1.12 | 0.027136213 |
| *PA4709* | -1.12 | 1.45E-06 |
| *PA4288* | -1.13 | 0.000696515 |
| *gcdH* | -1.13 | 1.78E-05 |
| *spuB* | -1.14 | 5.33E-18 |
| *PA4182* | -1.17 | 2.99E-05 |
| *PA5446* | -1.17 | 0.013865736 |
| *PA2086* | -1.20 | 0.022970811 |
| *dnaK* | -1.20 | 0.000267562 |
| *fdx2* | -1.20 | 0.000242878 |
| *PA3520* | -1.21 | 1.99E-07 |
| *PA4844* | -1.22 | 3.57E-08 |
| *PA1355* | -1.22 | 0.000353319 |
| *PA2091* | -1.22 | 0.042384275 |
| *PA4773* | -1.23 | 0.018604952 |
| *cdhC* | -1.23 | 0.011312269 |
| *PA4896* | -1.24 | 0.000528377 |
| *PA4878* | -1.25 | 2.16E-06 |
| *PA0534* | -1.26 | 1.74E-05 |
| *cdhB* | -1.26 | 0.009971875 |
| *PA3972* | -1.27 | 7.93E-09 |
| *PA5275* | -1.27 | 2.01E-07 |
| *PA1253* | -1.27 | 5.78E-08 |
| *PA1259* | -1.33 | 1.37E-07 |
| *PA3530* | -1.34 | 4.35E-05 |
| *PA2636* | -1.34 | 0.013865567 |
| *PA1255* | -1.34 | 7.18E-09 |
| *PA0535* | -1.35 | 0.002646574 |
| *cdhA* | -1.35 | 0.000409264 |
| *PA1864* | -1.36 | 0.025582361 |
| *PA3523* | -1.36 | 0.00010261 |
| *PA3428* | -1.38 | 0.035876721 |
| *PA4324* | -1.38 | 1.06E-15 |
| *PA3522* | -1.38 | 1.83E-05 |
| *PA1136* | -1.41 | 1.05E-09 |
| *iscS* | -1.43 | 4.92E-10 |
| *PA0172* | -1.43 | 3.11E-12 |
| *iscR* | -1.45 | 4.45E-10 |
| *PA4364* | -1.46 | 3.10E-07 |
| *PA4365* | -1.47 | 3.78E-09 |
| *acoB* | -1.47 | 1.24E-05 |
| *iscU* | -1.48 | 2.74E-08 |
| *PA1260* | -1.50 | 1.66E-15 |
| *ibpA* | -1.50 | 0.000136063 |
| *PA1256* | -1.51 | 1.90E-12 |
| *PA0433* | -1.53 | 0.000103844 |
| *PA3662* | -1.53 | 1.79E-14 |
| *PA5384* | -1.55 | 0.009033896 |
| *PA1257* | -1.55 | 7.03E-05 |
| *PA4153* | -1.57 | 1.39E-05 |
| *PA3920* | -1.57 | 3.41E-07 |
| *PA4148* | -1.58 | 1.39E-07 |
| *PA4152* | -1.71 | 1.21E-06 |
| *PA1169* | -1.72 | 1.25E-09 |
| *PA5084* | -1.73 | 3.79E-08 |
| *PA4843* | -2.10 | 8.28E-34 |
| *PA3574a* | -2.15 | 8.42E-17 |
| *PA1254* | -2.21 | 6.77E-16 |
| *PA5082* | -2.31 | 1.97E-14 |
| *PA0170* | -2.31 | 1.72E-07 |
| *PA4222* | -2.33 | 0.040804614 |
| *PA5083* | -2.42 | 9.83E-12 |
| *pchA* | -2.53 | 0.039439671 |
| *pchC* | -2.65 | 0.032291944 |
| *pchB* | -2.68 | 0.042167887 |
| *PA4218* | -2.69 | 4.88E-06 |
| *PA4219* | -2.78 | 0.000136046 |
| *pchD* | -2.78 | 0.034928868 |
| *PA4625* | -2.82 | 1.47E-30 |
| *pchE* | -2.88 | 0.031723179 |
| *PA4140* | -2.92 | 2.20E-39 |
| *PA1137* | -2.93 | 1.92E-08 |
| *PA4220* | -3.14 | 0.003246309 |
| *fptA* | -3.32 | 0.001771739 |
| *PA1168* | -3.79 | 5.47E-13 |
| *PA4139* | -5.21 | 1.25E-124 |

Table S5 Differential expressed gene in PAO1(*pqsE*) vs PAO1

| Gene | log2FoldChange | padj |
| --- | --- | --- |
| *pqsE* | 4.23 | 2.72E-93 |
| *PA0848* | 2.97 | 9.70E-10 |
| *katB* | 2.78 | 4.98E-08 |
| *PA3287* | 2.32 | 8.62E-07 |
| *trxB2* | 2.28 | 2.14E-06 |
| *antA* | 2.26 | 0.001965273 |
| *antB* | 2.24 | 0.002079841 |
| *phzG1* | 2.01 | 0.000418068 |
| *xylL* | 1.97 | 6.77E-05 |
| *antC* | 1.92 | 0.000875749 |
| *phzA2* | 1.76 | 0.002079841 |
| *phzB1* | 1.64 | 1.42E-06 |
| *ahpF* | 1.53 | 2.38E-07 |
| *phzC2* | 1.37 | 0.000170675 |
| *phzS* | 1.36 | 1.18E-08 |
| *PA3288* | 1.35 | 9.43E-08 |
| *phzC1* | 1.24 | 0.007750809 |
| *lecA* | 1.23 | 5.90E-16 |
| *phzG2* | 1.23 | 0.034489099 |
| *catA* | 1.20 | 3.88E-06 |
| *phzH* | 1.12 | 5.85E-13 |
| *PA2682* | 1.02 | 5.51E-05 |
| *phnA* | -1.02 | 0.000436796 |
| *pqsA* | -1.11 | 5.43E-05 |
| *pvdP* | -1.12 | 0.027519387 |
| *PA2409* | -1.12 | 0.017621958 |
| *pqsB* | -1.20 | 6.86E-05 |
| *PA1169* | -1.22 | 0.000281388 |
| *PA3574a* | -1.24 | 5.66E-05 |
| *pqsD* | -1.24 | 2.34E-06 |
| *pqsC* | -1.36 | 8.62E-07 |
| *PA1168* | -1.73 | 0.027802527 |
| *PA2412* | -1.75 | 0.02035266 |

Table S6 Differential expressed genes in PAO1(*pqsE*, *proE*) vs PAO1(*proE*)

| Gene | log2FoldChange | padj |
| --- | --- | --- |
| *pqsE* | 5.23 | 2.20E-143 |
| *ohr* | 3.83 | 5.23E-07 |
| *PA3237* | 2.45 | 1.49E-06 |
| *antA* | 2.13 | 0.001166185 |
| *metE* | 2.12 | 8.36E-15 |
| *PA3287* | 2.11 | 5.56E-06 |
| *phzC2* | 1.98 | 3.47E-10 |
| *ohrR* | 1.98 | 0.000331043 |
| *PA2868* | 1.95 | 5.28E-05 |
| *lecA* | 1.90 | 1.29E-40 |
| *rsmA* | 1.81 | 0.001116579 |
| *phzB2* | 1.81 | 0.000284007 |
| *antB* | 1.73 | 0.010417551 |
| *PA4612* | 1.72 | 0.00675943 |
| *antC* | 1.71 | 0.001506233 |
| *katB* | 1.69 | 0.001932358 |
| *phzG1* | 1.68 | 0.001900204 |
| *PA5392* | 1.66 | 0.00167753 |
| *PA0848* | 1.62 | 0.002341401 |
| *PA1154* | 1.61 | 0.003218887 |
| *PA0250* | 1.60 | 3.30E-16 |
| *phzC1* | 1.55 | 5.63E-05 |
| *PA2091* | 1.53 | 0.010101712 |
| *phzG2* | 1.52 | 0.000365753 |
| *phzA1* | 1.51 | 0.003202972 |
| *oprB* | 1.50 | 4.04E-20 |
| *chiC* | 1.49 | 1.27E-14 |
| *PA2805* | 1.45 | 0.001945593 |
| *PA0284* | 1.44 | 0.010246682 |
| *PA1216* | 1.40 | 7.82E-07 |
| *PA5460* | 1.40 | 0.000317925 |
| *PA1503* | 1.39 | 0.014182031 |
| *PA3187* | 1.37 | 1.34E-10 |
| *sdsA1* | 1.36 | 0.003680339 |
| *PA1907* | 1.36 | 1.91E-07 |
| *lecB* | 1.36 | 2.24E-23 |
| *PA4078* | 1.35 | 2.58E-09 |
| *phzH* | 1.35 | 8.84E-20 |
| *PA1506* | 1.34 | 0.008278269 |
| *bfrB* | 1.34 | 0.041877775 |
| *czcB* | 1.31 | 0.004104297 |
| *trxB2* | 1.31 | 0.0101785 |
| *mvaT* | 1.31 | 0.034237673 |
| *PA1355* | 1.31 | 0.000273938 |
| *cysW* | 1.30 | 8.30E-05 |
| *PA1289* | 1.28 | 1.58E-06 |
| *phzB1* | 1.27 | 0.000217637 |
| *PA3274* | 1.27 | 9.99E-05 |
| *xylZ* | 1.27 | 0.03083034 |
| *PA4384* | 1.25 | 3.31E-08 |
| *PA0346* | 1.25 | 0.033056737 |
| *PA2031* | 1.25 | 4.84E-05 |
| *PA4288* | 1.24 | 0.000373846 |
| *snr1* | 1.24 | 7.28E-09 |
| *PA1385* | 1.23 | 7.29E-05 |
| *phzA2* | 1.23 | 0.019799635 |
| *PA2589* | 1.23 | 7.99E-05 |
| *PA1217* | 1.23 | 1.49E-06 |
| *PA0883* | 1.23 | 0.016838541 |
| *PA3288* | 1.23 | 9.11E-07 |
| *PA0270* | 1.22 | 6.46E-11 |
| *PA2299* | 1.19 | 0.000494495 |
| *PA0937* | 1.17 | 0.000843102 |
| *PA3389* | 1.17 | 0.035114935 |
| *phzS* | 1.16 | 9.11E-07 |
| *PA1214* | 1.16 | 0.003849483 |
| *PA1545* | 1.15 | 5.33E-06 |
| *PA0983* | 1.15 | 0.018593063 |
| *PA2030* | 1.15 | 2.11E-07 |
| *PA2210* | 1.15 | 0.004602158 |
| *PA1138* | 1.14 | 0.037938287 |
| *PA1571* | 1.13 | 0.00920219 |
| *glpF* | 1.13 | 0.000879143 |
| *PA0187* | 1.13 | 4.47E-05 |
| *PA1870* | 1.12 | 2.05E-07 |
| *PA0918* | 1.12 | 4.25E-05 |
| *coxB* | 1.11 | 0.000192838 |
| *PA2457* | 1.10 | 0.001205637 |
| *crcZ* | 1.10 | 0.002332314 |
| *PA2021* | 1.10 | 0.00233875 |
| *PA2190* | 1.09 | 1.81E-05 |
| *PA4789* | 1.09 | 0.040062435 |
| *PA4141* | 1.08 | 5.41E-08 |
| *xylL* | 1.07 | 0.039209941 |
| *PA2321* | 1.07 | 0.028240526 |
| *PA1906* | 1.07 | 8.02E-07 |
| *PA1415* | 1.07 | 0.000140304 |
| *flgB* | 1.06 | 0.000228137 |
| *PA2140* | 1.06 | 0.009213158 |
| *PA2869* | 1.05 | 0.000224927 |
| *PA1213* | 1.05 | 0.00036578 |
| *PA1784* | 1.05 | 2.10E-08 |
| *hcnC* | 1.04 | 8.24E-05 |
| *katA* | 1.04 | 9.51E-05 |
| *PA0103a* | 1.02 | 0.010992247 |
| *PA0188* | 1.02 | 0.000215503 |
| *PA2768* | 1.02 | 0.025552407 |
| *PA0389* | -1.00 | 0.015129213 |
| *rpmD* | -1.02 | 4.61E-07 |
| *PA4355* | -1.02 | 9.17E-05 |
| *PA0909* | -1.02 | 0.028130407 |
| *pvdP* | -1.04 | 0.010681251 |
| *PA0378* | -1.04 | 0.003680339 |
| *PA1103* | -1.06 | 0.000482745 |
| *lnt* | -1.07 | 9.53E-05 |
| *PA3222* | -1.07 | 2.09E-05 |
| *PA1394* | -1.10 | 0.037702172 |
| *PA4169* | -1.10 | 0.032977523 |
| *pqsC* | -1.13 | 5.68E-05 |
| *pqsB* | -1.13 | 0.000114433 |
| *PA3335* | -1.14 | 2.36E-06 |
| *rpmF* | -1.18 | 0.009383941 |
| *PA3265* | -1.19 | 0.001811082 |
| *estX* | -1.20 | 0.001403112 |
| *pilK* | -1.21 | 4.06E-05 |
| *PA0370* | -1.23 | 8.93E-05 |
| *PA1970* | -1.27 | 0.020991164 |
| *PA0369* | -1.34 | 1.20E-06 |
| *plsX* | -1.37 | 3.32E-05 |
| *narI* | -1.50 | 0.000467646 |
| *PA2115* | -1.52 | 0.001916331 |
| *pqsA* | -1.56 | 1.82E-10 |
| *PA2393* | -1.63 | 0.014717299 |
| *PA4122* | -1.64 | 0.005605648 |
| *PA4861* | -1.67 | 0.021153368 |
| *PA4882* | -1.84 | 0.000214419 |
| *PA0682* | -2.26 | 0.006507326 |

Table S7 Differential expressed genes in PAO1(*pqsE*, *proE*) vs PAO1(*pqsE*)

| *Gene* | log2FoldChange | padj |
| --- | --- | --- |
| *proE* | 7.47 | 3.31E-200 |
| *ohr* | 4.12 | 1.10E-08 |
| *rsmA* | 2.88 | 4.09E-09 |
| *betB* | 2.70 | 1.14E-48 |
| *opdH* | 2.63 | 3.04E-13 |
| *PA1410* | 2.59 | 8.16E-26 |
| *PA0754* | 2.28 | 2.69E-18 |
| *PA2146* | 2.20 | 2.29E-06 |
| *cobI* | 2.20 | 2.58E-16 |
| *PA0668.4* | 2.18 | 0.0112264 |
| *betI* | 2.08 | 4.89E-21 |
| *PA2190* | 2.00 | 2.62E-19 |
| *PA2143* | 1.99 | 1.67E-36 |
| *PA1711* | 1.99 | 4.74E-20 |
| *PA5352* | 1.99 | 0.007797398 |
| *ohrR* | 1.98 | 0.000159391 |
| *piv* | 1.91 | 6.93E-17 |
| *phzG2* | 1.91 | 8.66E-07 |
| *PA2868* | 1.89 | 3.31E-05 |
| *aphA* | 1.89 | 1.24E-10 |
| *PA2139* | 1.87 | 3.13E-06 |
| *katE* | 1.84 | 1.29E-12 |
| *phzB2* | 1.82 | 0.000121622 |
| *PA0752* | 1.81 | 1.99E-11 |
| *PA4738* | 1.81 | 3.14E-16 |
| *PA3933* | 1.78 | 1.67E-16 |
| *PA2294* | 1.78 | 4.44E-06 |
| *PA3287* | 1.75 | 0.000135083 |
| *glcF* | 1.73 | 0.046114506 |
| *pscG* | 1.73 | 5.77E-11 |
| *PA2162* | 1.66 | 1.20E-12 |
| *PA2134* | 1.66 | 2.63E-08 |
| *PA2141* | 1.65 | 3.10E-10 |
| *PA2140* | 1.65 | 4.44E-06 |
| *PA4612* | 1.64 | 0.0083729 |
| *betA* | 1.62 | 6.17E-29 |
| *PA2166* | 1.61 | 6.36E-13 |
| *phzC2* | 1.59 | 3.95E-07 |
| *popB* | 1.58 | 1.43E-08 |
| *PA3274* | 1.58 | 1.22E-07 |
| *PA0848* | 1.58 | 0.001992237 |
| *PA1870* | 1.57 | 2.28E-15 |
| *katN* | 1.57 | 1.24E-09 |
| *glcD* | 1.56 | 0.034454666 |
| *PA1592* | 1.56 | 0.04780358 |
| *PA2805* | 1.56 | 0.000409587 |
| *PA2161* | 1.56 | 3.75E-10 |
| *PA2184* | 1.54 | 6.40E-12 |
| *PA2163* | 1.53 | 1.37E-11 |
| *PA2180* | 1.53 | 3.89E-11 |
| *popD* | 1.53 | 8.79E-10 |
| *PA2165* | 1.53 | 6.40E-12 |
| *PA2021* | 1.52 | 2.31E-06 |
| *crcZ* | 1.52 | 2.42E-06 |
| *pscI* | 1.51 | 2.72E-05 |
| *PA2138* | 1.51 | 9.71E-14 |
| *PA4172* | 1.50 | 1.33E-10 |
| *PA1699* | 1.50 | 7.82E-05 |
| *PA2150* | 1.49 | 4.48E-13 |
| *exoS* | 1.48 | 3.75E-10 |
| *PA0130* | 1.48 | 4.23E-06 |
| *PA1961* | 1.45 | 2.63E-11 |
| *PA2159* | 1.45 | 1.96E-12 |
| *katB* | 1.45 | 0.00738187 |
| *chiC* | 1.43 | 1.15E-13 |
| *PA2158* | 1.43 | 7.41E-10 |
| *PA0250* | 1.42 | 3.26E-13 |
| *PA4171* | 1.41 | 1.60E-12 |
| *PA2164* | 1.41 | 1.69E-10 |
| *aruD* | 1.40 | 9.71E-06 |
| *exsB* | 1.40 | 2.41E-06 |
| *PA2148* | 1.40 | 1.35E-12 |
| *PA2152* | 1.40 | 4.01E-09 |
| *PA2149* | 1.39 | 1.33E-05 |
| *PA2188* | 1.39 | 5.15E-08 |
| *PA2137* | 1.38 | 0.00013737 |
| *PA2135* | 1.37 | 6.54E-10 |
| *PA2179* | 1.35 | 2.41E-07 |
| *pcrH* | 1.35 | 0.020012268 |
| *pcrG* | 1.35 | 0.00273486 |
| *PA2154* | 1.34 | 5.05E-10 |
| *PA0912* | 1.34 | 0.00069058 |
| *pscJ* | 1.33 | 4.01E-09 |
| *PA3237* | 1.33 | 0.014378409 |
| *PA2321* | 1.33 | 0.003251813 |
| *PA0346* | 1.33 | 0.019748897 |
| *exsD* | 1.32 | 3.94E-08 |
| *PA2171* | 1.32 | 6.22E-08 |
| *PA1907* | 1.31 | 1.48E-07 |
| *PA2299* | 1.31 | 3.33E-05 |
| *glgB* | 1.31 | 5.17E-08 |
| *PA2160* | 1.30 | 1.50E-07 |
| *PA4877* | 1.28 | 4.26E-12 |
| *glgP* | 1.27 | 3.89E-11 |
| *PA2157* | 1.27 | 5.99E-08 |
| *PA2172* | 1.27 | 3.15E-09 |
| *PA1508* | 1.25 | 0.000221962 |
| *aruB* | 1.24 | 0.015404601 |
| *aruF* | 1.21 | 0.000222384 |
| *PA1216* | 1.21 | 1.12E-05 |
| *cbpD* | 1.21 | 2.63E-08 |
| *aruC* | 1.21 | 1.38E-06 |
| *PA0751* | 1.21 | 2.96E-06 |
| *aprE* | 1.20 | 0.000844361 |
| *aprA* | 1.19 | 1.84E-09 |
| *exoY* | 1.18 | 2.80E-07 |
| *aruG* | 1.18 | 0.000265711 |
| *exsC* | 1.18 | 1.11E-08 |
| *lasA* | 1.17 | 7.49E-05 |
| *PA1323* | 1.17 | 4.09E-09 |
| *PA2939* | 1.17 | 2.96E-07 |
| *pcrV* | 1.16 | 9.27E-07 |
| *PA1324* | 1.16 | 4.59E-08 |
| *PA0713* | 1.16 | 4.14E-05 |
| *PA1571* | 1.15 | 0.006068573 |
| *PA2151* | 1.13 | 4.78E-09 |
| *PA2108* | 1.13 | 1.56E-07 |
| *PA0132* | 1.13 | 0.000718928 |
| *pscB* | 1.13 | 0.001361818 |
| *PA2155* | 1.13 | 5.28E-06 |
| *PA3488* | 1.12 | 0.000414404 |
| *exoT* | 1.12 | 1.27E-07 |
| *PA2145* | 1.11 | 2.04E-08 |
| *PA2173* | 1.11 | 2.74E-05 |
| *PA2142* | 1.11 | 4.66E-09 |
| *PA3288* | 1.11 | 4.81E-06 |
| *oprB* | 1.11 | 2.28E-11 |
| *PA1509* | 1.11 | 9.68E-06 |
| *PA2107* | 1.10 | 0.000206468 |
| *PA1906* | 1.09 | 9.36E-08 |
| *PA0131* | 1.09 | 0.039055994 |
| *PA0645* | 1.09 | 0.004570407 |
| *atoB* | 1.09 | 1.17E-05 |
| *PA4499* | 1.07 | 0.000177371 |
| *PA2082* | 1.07 | 0.001139372 |
| *PA2621* | 1.07 | 8.60E-05 |
| *PA2192* | 1.07 | 0.007006232 |
| *PA0060* | 1.06 | 7.87E-08 |
| *PA1510* | 1.05 | 2.79E-07 |
| *PA0052* | 1.05 | 0.000207968 |
| *PA2575* | 1.04 | 0.003928941 |
| *cobP* | 1.04 | 0.008040388 |
| *PA2181* | 1.04 | 5.28E-07 |
| *PA0200* | 1.03 | 0.003054173 |
| *PA0572* | 1.03 | 2.00E-07 |
| *pscQ* | 1.03 | 0.002591231 |
| *osmC* | 1.03 | 2.74E-10 |
| *PA4498* | 1.02 | 1.06E-06 |
| *PA3842* | 1.01 | 9.20E-07 |
| *PA2170* | 1.01 | 0.00341416 |
| *pscD* | 1.00 | 0.000359299 |
| *PA4896* | -1.00 | 0.00838911 |
| *PA4152* | -1.01 | 0.010401583 |
| *carA* | -1.03 | 2.00E-07 |
| *PA5510* | -1.03 | 3.91E-05 |
| *hasE* | -1.04 | 0.040941115 |
| *PA4584* | -1.06 | 0.000447336 |
| *PA5508* | -1.06 | 4.47E-06 |
| *nosZ* | -1.09 | 0.000428706 |
| *PA0475* | -1.10 | 0.000514668 |
| *PA5030* | -1.11 | 0.001360424 |
| *PA1169* | -1.11 | 0.000269435 |
| *PA3662* | -1.12 | 7.82E-08 |
| *PA4181* | -1.13 | 6.11E-06 |
| *PA1622* | -1.13 | 5.31E-05 |
| *PA0939* | -1.13 | 0.019748897 |
| *PA4878* | -1.14 | 2.26E-05 |
| *PA1195* | -1.16 | 5.91E-06 |
| *PA0433* | -1.16 | 0.005804771 |
| *ibpA* | -1.17 | 0.00535473 |
| *PA5081* | -1.17 | 0.000234641 |
| *arsC* | -1.17 | 5.56E-06 |
| *PA3920* | -1.17 | 0.000308744 |
| *PA0172* | -1.18 | 2.23E-08 |
| *PA2282* | -1.21 | 3.96E-05 |
| *PA2115* | -1.21 | 0.014451948 |
| *PA4324* | -1.23 | 2.36E-12 |
| *PA1255* | -1.23 | 1.42E-07 |
| *PA3521* | -1.23 | 1.01E-10 |
| *PA4895* | -1.23 | 3.06E-05 |
| *PA4882* | -1.24 | 0.016935075 |
| *PA4153* | -1.24 | 0.001139372 |
| *PA0169* | -1.24 | 1.12E-05 |
| *PA0534* | -1.24 | 2.48E-05 |
| *PA0446* | -1.25 | 3.94E-08 |
| *PA1256* | -1.25 | 1.27E-08 |
| *PA4182* | -1.27 | 4.42E-06 |
| *PA0170* | -1.27 | 0.010017114 |
| *PA5507* | -1.29 | 0.000454048 |
| *pchR* | -1.30 | 3.21E-06 |
| *cdhC* | -1.31 | 0.007571777 |
| *PA4122* | -1.32 | 0.028034855 |
| *PA4586* | -1.33 | 0.000449607 |
| *PA4364* | -1.33 | 4.15E-06 |
| *PA0535* | -1.34 | 0.00330001 |
| *cdhA* | -1.34 | 0.000644296 |
| *PA0278* | -1.38 | 2.14E-05 |
| *PA2427* | -1.42 | 8.18E-06 |
| *PA3574a* | -1.47 | 6.36E-08 |
| *PA0683* | -1.52 | 0.017052155 |
| *PA1260* | -1.53 | 6.36E-16 |
| *PA1259* | -1.53 | 6.57E-10 |
| *PA4365* | -1.57 | 2.85E-10 |
| *gcdH* | -1.58 | 3.13E-10 |
| *PA3523* | -1.61 | 2.96E-06 |
| *PA3522* | -1.67 | 1.22E-07 |
| *PA3591* | -1.71 | 0.020807174 |
| *cdhB* | -1.81 | 0.000156778 |
| *PA1168* | -1.85 | 0.001786194 |
| *PA1254* | -1.88 | 1.45E-11 |
| *PA4843* | -1.90 | 2.13E-27 |
| *PA5084* | -1.93 | 4.52E-10 |
| *PA4861* | -2.15 | 0.001139372 |
| *PA0682* | -2.30 | 0.003928941 |
| *PA4140* | -2.40 | 1.83E-26 |
| *PA5082* | -2.62 | 1.67E-18 |
| *PA1137* | -2.77 | 1.26E-07 |
| *PA4625* | -2.79 | 9.01E-30 |
| *PA5083* | -2.87 | 3.45E-16 |
| *PA4218* | -2.97 | 2.73E-07 |
| *PA4222* | -3.09 | 0.003853941 |
| *PA4219* | -3.22 | 5.64E-06 |
| *pchA* | -3.44 | 0.002518733 |
| *pchG* | -3.52 | 0.013577546 |
| *pchF* | -3.55 | 0.007817503 |
| *PA4223* | -3.70 | 0.00734433 |
| *pchB* | -3.76 | 0.002062084 |
| *pchC* | -3.80 | 0.000914848 |
| *pchE* | -3.81 | 0.002430631 |
| *pchD* | -3.82 | 0.001900139 |
| *PA4139* | -3.89 | 3.59E-69 |
| *fptA* | -4.00 | 0.000108819 |
| *PA4220* | -4.37 | 1.40E-05 |


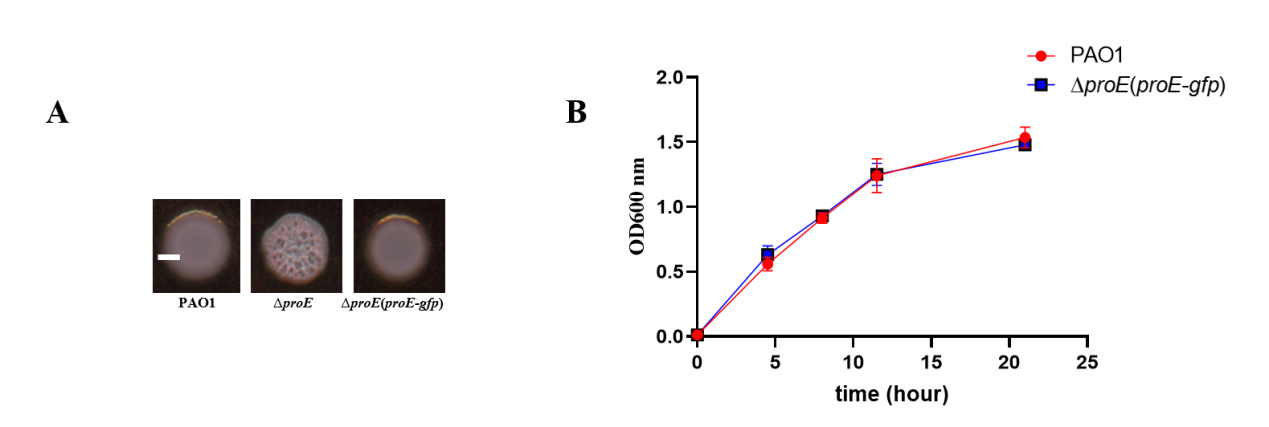


**Supplementary Figure S1. Congo-red colony morphology assay and growth curve.** (A) Colony morphology of *P. aeruginosa* strain PAO1 and its derivatives on Congo-Red plates, Scale bar = 2 mm. (B) Growth curves of PAO1 and ***△****proE(proE-gfp)*. Three independent experiments were performed in triplicate.


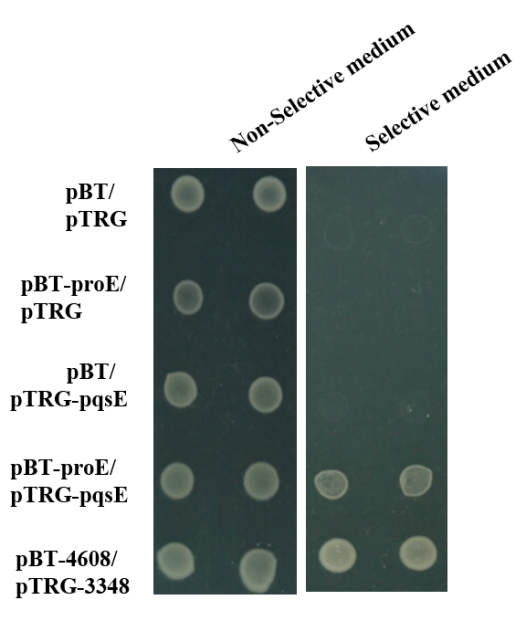


**Supplementary Figure S2. Testing self-activation by recombinant pBT or pTRG.** Bacterial two-hybrid assay suggests direct interaction between ProE and PqsE, and without self-activation. 3348- 4608 pair is the positive control.


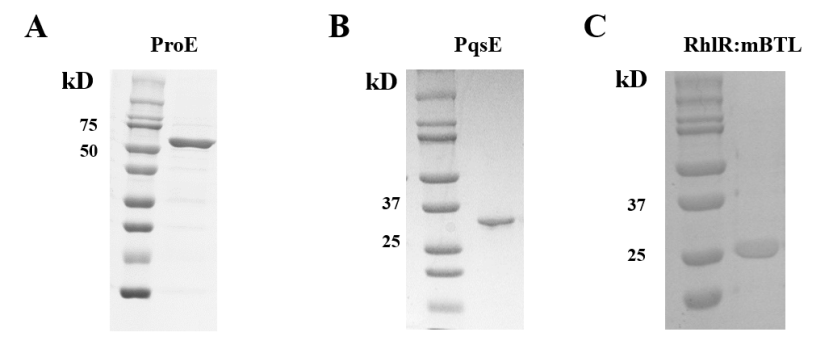


**Supplementary Figure S3. Purification of ProE, PqsE and RhlR:mBTL**. Purified ProE (A), PqsE (B), RhlR:mBTL (C).


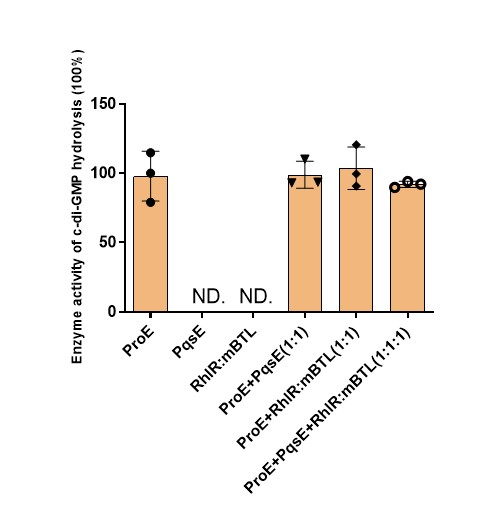


**Supplementary Figure S4.** **Measurement of the c-di-GMP hydrolysis activity in vitro.** The enzymatic activity of ProE, PqsE or RhlR to c-di-GMP was measured, and the influence of PqsE, RhlR to ProE’s activity was also measured. The experiment was performed at least three times in triplicate. The data presented are the means of replicates, and error bars represent the standard deviation.


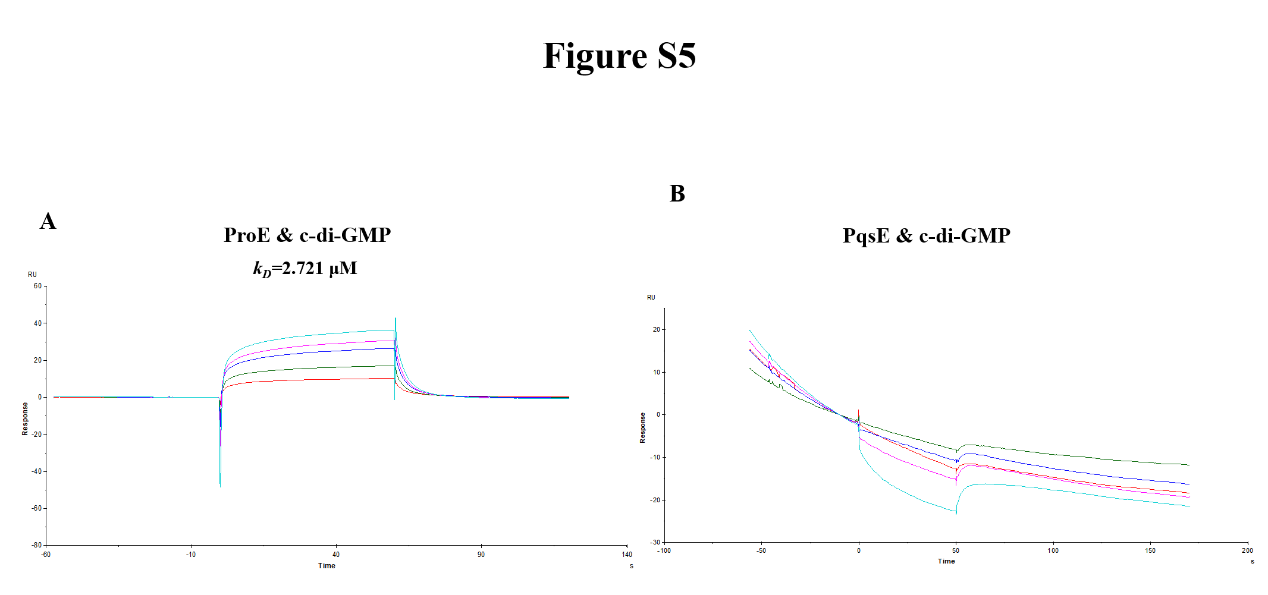


**Supplementary Figure S5.** **Measurement of the c-di-GMP binding by SPR**. The binding of ProE (A) and PqsE (B) to c-di-GMP were measured. Plots are representative of three experiments with similar results. RU, response units; *k_D_*, dissociation constant.


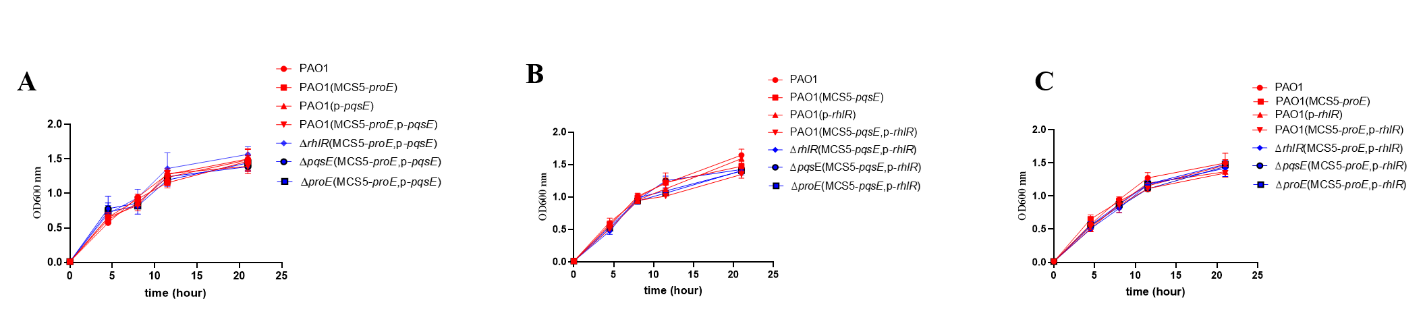


**Supplementary Figure S6.** Growth curves of different strains. The data are means of three replicates and error bars indicate standard deviation.


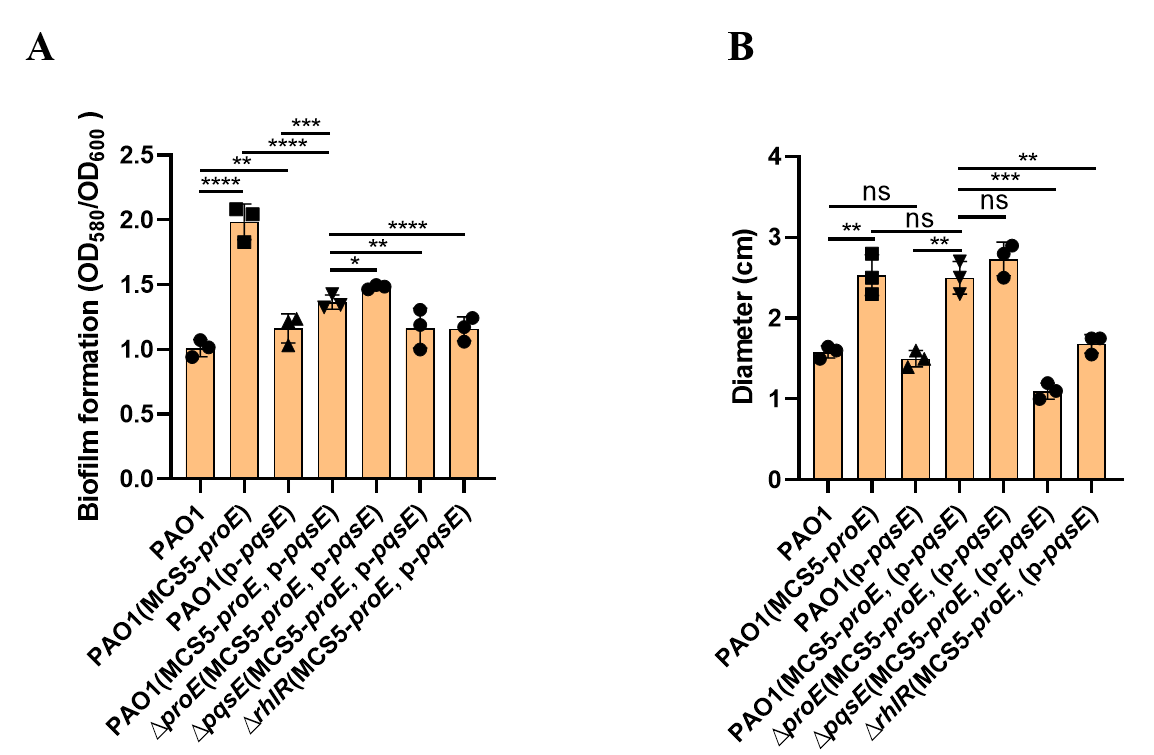


**Supplementary Figure S7. Influence of ProE-PqsE on biofilm formation and swimming motility**. (A) Biofilm formation of various strains were measured. (B) The swimming motility of various strains were measured. Three independent experiments were performed in triplicate. **P* < 0.05, ***P* < 0.01, ****P* < 0.001, *****P* < 0.0001, ns, not significant.

**Reference**

1. Feng Q, Ahator SD, Zhou T, Liu Z, Lin Q, Liu Y, Huang J, Zhou J, Zhang LH. 2020. Regulation of Exopolysaccharide Production by ProE, a Cyclic-Di-GMP Phosphodiesterase in Pseudomonas aeruginosa PAO1. Front Microbiol 11:1226.

2. Lin Chua S, Liu Y, Li Y, Jun Ting H, Kohli GS, Cai Z, Suwanchaikasem P, Kau Kit Goh K, Pin Ng S, Tolker-Nielsen T, Yang L, Givskov M. 2017. Reduced Intracellular c-di-GMP Content Increases Expression of Quorum Sensing-Regulated Genes in Pseudomonas aeruginosa. Front Cell Infect Microbiol 7:451.

3. Yang L, Barken KB, Skindersoe ME, Christensen AB, Givskov M, Tolker-Nielsen T. 2007. Effects of iron on DNA release and biofilm development by Pseudomonas aeruginosa. Microbiology (Reading) 153:1318-1328.

4. Kovach ME, Elzer PH, Hill DS, Robertson GT, Farris MA, Roop RM, 2nd, Peterson KM. 1995. Four new derivatives of the broad-host-range cloning vector pBBR1MCS, carrying different antibiotic-resistance cassettes. Gene 166:175-6.

5. Rybtke MT, Borlee BR, Murakami K, Irie Y, Hentzer M, Nielsen TE, Givskov M, Parsek MR, Tolker-Nielsen T. 2012. Fluorescence-based reporter for gauging cyclic di-GMP levels in Pseudomonas aeruginosa. Appl Environ Microbiol 78:5060-9.

6. Figurski DH, Helinski DR. 1979. Replication of an origin-containing derivative of plasmid RK2 dependent on a plasmid function provided in trans. Proc Natl Acad Sci U S A 76:1648-52.
